# Tuning UV Absorption in Imine-Linked Covalent Organic Frameworks via Methylation

**DOI:** 10.1021/acs.jpcc.2c04586

**Published:** 2022-11-24

**Authors:** Ellen Dautzenberg, Milena Lam, Tatiana Nikolaeva, Wouter M. J. Franssen, Barend van Lagen, Ilse P. A. M. Gerrits-Benneheij, Nikolay Kosinov, Guanna Li, Louis C. P. M. de Smet

**Affiliations:** † Laboratory of Organic Chemistry, Wageningen University, Stippeneng 4, 6708 WEWageningen, The Netherlands; ‡ MAGNEtic Resonance Research FacilitY-MAGNEFY, Wageningen University, Stippeneng 4, 6708 WEWageningen, The Netherlands; § Laboratory of Biophysics, Wageningen University, Stippeneng 4, 6708 WEWageningen, The Netherlands; ∥ Environmental Technology, Wageningen University, Bornse Weilanden 9, 6708 WGWageningen, The Netherlands; ⊥ Laboratory of Inorganic Materials and Catalysis, Department of Chemical Engineering and Chemistry, Eindhoven University of Technology, P.O. Box 513, 5600 MBEindhoven, The Netherlands; # Biobased Chemistry and Technology, Wageningen University, Bornse Weilanden 9, 6708 WGWageningen, The Netherlands

## Abstract

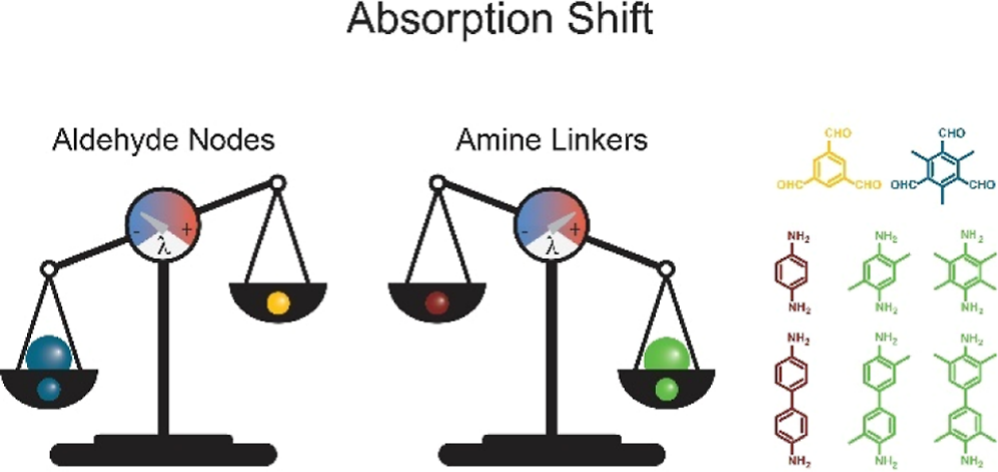

Covalent organic frameworks (COFs) are porous materials with high surface areas, making them interesting for a large variety of applications including energy storage, gas separation, photocatalysis, and chemical sensing. Structural variation plays an important role in tuning COF properties. Next to the type of the building block core, bonding directionality, and linking chemistry, substitution of building blocks provides another level of synthetic control. Thorough characterization and comparison of various substitution patterns is relevant for the molecular engineering of COFs via rational design. To this end, we have systematically synthesized and characterized multiple combinations of several methylated and non-methylated building blocks to obtain a series of imine-based COFs. This includes the experimental assignment of the COF structure by solid-state NMR. By comparing the properties of all COFs, the following trends were found: (1) upon methylation of the aldehyde nodes, COFs show increased Brunauer–Emmett–Teller surface areas, reduced pore collapse, blue-shifted absorbance spectra, and ∼0.2 eV increases in their optical band gaps. (2) COFs with dimethylated amine linkers show a lower porosity. (3) In tetramethylated amine linkers, the COF porosity even further decreases, the absorbance spectra are clearly red-shifted, and smaller optical band gaps are obtained. Our study shows that methyl substitution patterns on COF building blocks are a handle to control the UV absorbance of the resulting frameworks.

## Introduction

Porous materials are a class of solids with pore sizes of a few to several hundred nanometers. These pores can be classified into macro- (>50 nm), meso- (2–50 nm), and micropores (<2 nm).^1^ Besides zeolites and metal organic frameworks, crystalline covalent organic frameworks (COFs)^2^ have gained increasing interest, and a huge variety of different materials and applications have been reported.^3,4^ The permanent porosity and the channel-like structure of COFs lead to high surface areas, which are of interest for, among others, energy storage,^3,5^ gas separation,^3,6^ chemical sensing,^3,7^ and photocatalysis.^3,8,9^ In the last-mentioned example, solar energy was used to split, for instance, water or carbon dioxide into hydrogen and oxygen gas or hydrocarbons, respectively.^8^ These gases can be used as energy sources or starting materials for other chemical processes. COFs have been reported as good photocatalysts for such clean and green transformations.^8^ The first COFs were linked by boronic esters, which were susceptible to hydrolysis. This has been improved by using imine-linked COFs or even more stable β-ketoenamine COFs.^10^ In the latter case, imine bonds are formed first, which then tautomerize irreversibly into the β-ketoenamine linkages. The irreversible tautomerization results in lower crystallinity and surface area compared to their imine equivalents, which is the reason why imine COFs are widely used.^4^ Imine COFs can be synthesized in a condensation reaction from aldehyde and amine building blocks under acidic catalysis, which is also known as Schiff base chemistry.^3,11−13^ Vacuum drying has often been used but was identified to induce partial pore collapse due to capillary forces.^14^ Capillary forces increase with decreasing pore sizes and COFs, which usually have pore sizes around a few nanometers, are therefore subjected to strong capillary forces. To overcome pore collapse, milder activation methods such as supercritical CO_2_ drying^14^ or washing with ultralow surface tension solvents prior to drying^15^ were suggested to keep the framework intact. Feriante et al.^14^ and Zhu and Verduzco^15^ both reported a methoxy-functionalized, imine-based COF, which was less prone to pore collapse compared to the initial unfunctionalized COF. Recently, we have found that COFs consisting of 2,4,6-trimethylbenzene-1,3,5-carbaldehyde (Me_3_TFB) lead to more robust frameworks compared to non-methylated 1,3,5-benzenetricarbaldehyde (TFB).^16^

While COF stability is essential for virtually all applications, other properties, like selectivity and catalytical performance, require tailor-made approaches. Identification of structure–property relationships of COFs would facilitate the process of controlling such properties. Karak et al.^17^ reported on how an acidic catalyst influences the porosity of obtained COF materials. Namely, they studied the effect of amine substitution on β-ketoenamine-linked COF properties. They investigated the porosity based on differences in hydrogen bonding in an intermediate to be able to predict the porosity of the resulting COFs. These β-ketoenamine-linked COFs were synthesized via a mechanochemical synthesis of 1,3,5-triformylphloroglucinol (Tp) with several functionalized phenylenediamine (PA) and benzidine (BD) linkers, catalyzed by different toluenesulfonic acids or hydrogen chloride. During this process, the diamine first forms an acid-diamine salt with the toluenesulfonic acids, which then further reacts with Tp to the final COF. The hydrogen-bond distance of the acid-diamine salt was found to affect the COF porosity. While the hydrogen-bond distance was tuned by the choice of toluenesulfonic acid, no trend between the Brunauer–Emmett–Teller (BET) area and their methylated amine linker was observed. Additionally, the applied mechanochemical synthesis—especially the intermediate acid-diamine salt—differs from COFs synthesized in solution. In another study, Singh et al.^9^ reported on the possibility to fine-tune the optical band gap by changing the number of hydroxyl groups on the TFB aldehyde node by varying the push–pull electronic effect. The authors found that the more pronounced push–pull effect leads to the lower resulting band gap. Combined with the earlier reported increased stability of methyl-substituted COFs,^16^ these two studies motivated us to systematically investigate the effect of methyl groups on the porosity and UV absorbance of imine-based COFs. To this end and given their pore stability and utilization potential of their UV absorbance, we decided to introduce an increasing number of methyl groups, this time also in the amine linkers. Such thorough comparison of similar, yet different structures, enables a fundamental understanding, which can be employed to rationally design COF materials with tailor-made properties. By expanding our methylated imine-based COF library and by thoroughly characterizing, including detailed solid-state NMR (ssNMR) measurements as well as experimental band gap analyses, we investigate the influence of methylation of aldehyde nodes and amine linkers on the resulting COF crystallinity, BET surface areas, the tendency for pore collapse, and the UV absorbance of the COFs.

## Materials and Methods

1,4-Phenylenediamine (>98% (GC) (T)), 2,5-dimethyl-1,4-phenylenediamine (>98%), and o-tolidine (>98%) were purchased from TCI Europe N.V. 2,3,5,6-Tetramethyl-1,4-phenylenediamine (>98% (GC) (T)) was purchased from Sigma-Aldrich, benzidine (98%) was purchased form Abcr, 2,3,5,6-tetramethylbenzidine (99%) was purchased from ChemPUR, and all chemicals were used without further purification. 1,3,5-Benzenetricarboxaldehyde (96%) was purchased from TCI Europe N.V. and Fluorochem. Mesitylene (99%, extra pure) was purchased from Fisher Scientific, and 1,4-dioxane (99%) was purchased from Acros Organics B.V.B.A. 2,5,6-Trimethyl-1,3,5-benzenetricarboxaldehyde was synthesized before.^16^ All solvents and glacial acetic acid (AR) were purchased from commercial sources and used without further purifications.

^1^H and ^13^C NMR spectra were recorded on a Bruker AVANCE III NMR spectrometer at 400 and 100 MHz, respectively. The spectra were referenced with respect to the deuterated solvents (CDCl_3_: 7.26 ppm, 77.16 ppm, DMSO-*d*_6_: 2.5 ppm, and 39.52 ppm). ^1^H and ^13^C{^1^H} cross-polarization magic angle spinning (CPMAS) ssNMR spectra were recorded on a Bruker AVANCE III HD spectrometer at 700.13 MHz (16.4 T) and 176 MHz, respectively. ssNMR samples were packed into 4 mm zirconia rotors and spun at MAS frequencies of 11 and 14 kHz at 298 K. The ^13^C CPMAS spectra were recorded by using a CP pulse sequence. SPINAL64 decoupling was applied on protons with a decoupling strength of 104 kHz. The ^13^C CPMAS spectra were obtained with a recycle delay of 3 s, and the strength of the CP contact pulses of 0.08 kHz related to a contact time of 3 ms, unless stated differently. To obtain the CP build-up curves, the CP contacted pulses were varied between 0.04 and 25 kHz and related to contact times between 6.5 ms and 10 μs. The ^13^C ssNMR spectra were referenced with respect to adamantane (^13^C, 29.456 ppm). The spectra were analyzed using MestReNova (version 14.1.0) and ssNake,^18^ which allows a fitting of the signals, opening up the possibility to integrate the signals. These relative peak integrals can be plotted against the contact time to obtain CP build-up curves.

Fourier-transform infrared (FT-IR) spectra were obtained on a Bruker Tensor 27 spectrometer with an attenuated total reflection accessory called platinum. The samples were applied as powder on top of the crystal. 64 scans were performed with a resolution of 4 cm^–1^.

Powder X-ray diffraction measurements were performed with a Philips X’pert-PRO at 40 kV and 40 mA from 4 to 40° (step size: 0.05°, step time: 90 s, mask in front of entrance: 10 mm and slit 1°, and slit before detector: 1°) and from 1.5 to 10° (step size: 0.05°, step time: 500 s, mask in front of entrance: 5 mm and slit 0.5°, and slit before detector: 0.25°). X-rays were generated by a Cu anode Kα (1.54 λ) radiation. The goniometer radius was 240 mm, the soller slits were 2.3°, and the receiving slit was 0.1 mm in width. Pawley refinement was carried out in Topas (version 5) with a CuKa5_Berger emission profile. The parameters “Zero point error”, “Cry Size L” , and “Strain L” were allowed to refine.

Nitrogen adsorption–desorption measurements were performed on a MicroActive for Tristar II Plus 2.01 at 77.350 K. Before the measurement, the samples were outgassed at 120 °C overnight. Surface areas were calculated from the adsorption data using BET methods and Rouquerol criteria. The pore-size distribution curves were obtained from the adsorption branches using the two different density-functional theory (DFT) methods:

(1) N2—cylindrical pores—oxide surface.

(2) HS-2D-NLDFT, carbon cylindrical pores (ZTC), and N_2_@77K.

The second method is carbon based and therefore chemically closer, but the first method can show the pore size distribution for some COFs down to smaller diameters. An optimum between Goodness of Fit and smoothness of the pore size distribution was aimed for. The average of three different COF batches was used to determine the BET surface areas.

The retained surface area was calculated based on Eq 1
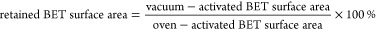
1

Diffuse reflectance spectra were recorded on a Cary4000 spectrometer with an integrating sphere from Agilent. An empty sample holder was used for calibration of 100% light intensity at the detector and a black surface for 0% light intensity.

Thermogravimetric analysis was performed on a PerkinElmer STA 6000. The sample was heated to 30 °C, and this temperature was maintained for 1 min, before the sample was heated with 10 °C/min to 700 °C in a nitrogen atmosphere with a flow rate of 20 ml/min. The thermal stability was determined at the point where 95% of the samples were still retained.

Origin2020b (64 bit) version 9.7.5.184 was used to analyze, plot, and fit all data.

All DFT calculations were performed by using the Vienna ab initio simulation package (VASP, version 5.4.4).^19,20^ The PBE functional based on the generalized gradient approximation was chosen to account for the exchange–correlation energy.^21^ A plane-wave basis set in combination with the projected augmented wave method was used to describe the valence electrons and the valence–core interactions, respectively.^22^ The kinetic energy cut-off of the plane wave basis set was set to 500 eV. Gaussian smearing of the population of partial occupancies with a width of 0.05 eV was used during iterative diagonalization of the Kohn–Sham Hamiltonian. The threshold for energy convergence for each iteration was set to 10–5 eV. The crystal structures were fully relaxed, and geometries were assumed to be converged when forces on each atom were less than 0.05 eV/Å. The Brillouin zone integration and *k*-point sampling were done with a γ centered 1*1*8 and 2*2*4 grid points for the eclipsed and staggered unit cells, respectively. The van der Waals interactions were included by using Grimme’s DFT-D3(BJ) method as implemented in VASP.^23^ Geometries were visualized, and simulated powder-X-ray diffraction (PXRD) patterns were obtained by using VESTA (version 3.4.8).^24^ Coordinates of all crystal structures are provided at the end of this article.

### General Procedure COF Synthesis

The COF synthesis is based on a modified procedure of Smith et al.^25^

The aldehyde (1 equiv) and amine monomers (1.5 equiv) were added to a 50 mL round bottom flask, together with a stirring rod, and were dissolved in 4:1 v/v 1,4-dioxane:mesitylene mixture. The mixture was heated to 70 °C for 5 min to ensure dissolution. After cooling the mixture to approximately 40 °C, water and glacial acetic acid (for exact amounts, see the respective COF) were added. The reaction mixture was stirred at 70 °C for 3 days. Afterward, the reaction was cooled to RT, and the precipitate was collected via Büchner filtration. The solid was dispersed in dimethylformamide (DMF), stirred at 90 °C for 30 min, and collected via Büchner filtration. These steps were repeated with DMF (90 °C, 30 min), ethanol (80 °C, 30 min), acetone (60 °C, 30 min), and hexane (70 °C, 30 min). After the final Büchner filtration, the COFs were divided over two petri dishes and covered with tin foil for drying. The COFs were dried overnight at 120 °C, either in a regular oven or in a vacuum oven. After drying, the COFs were kept in the glovebox for storage.

## Results and Discussion

**Figure 1:**
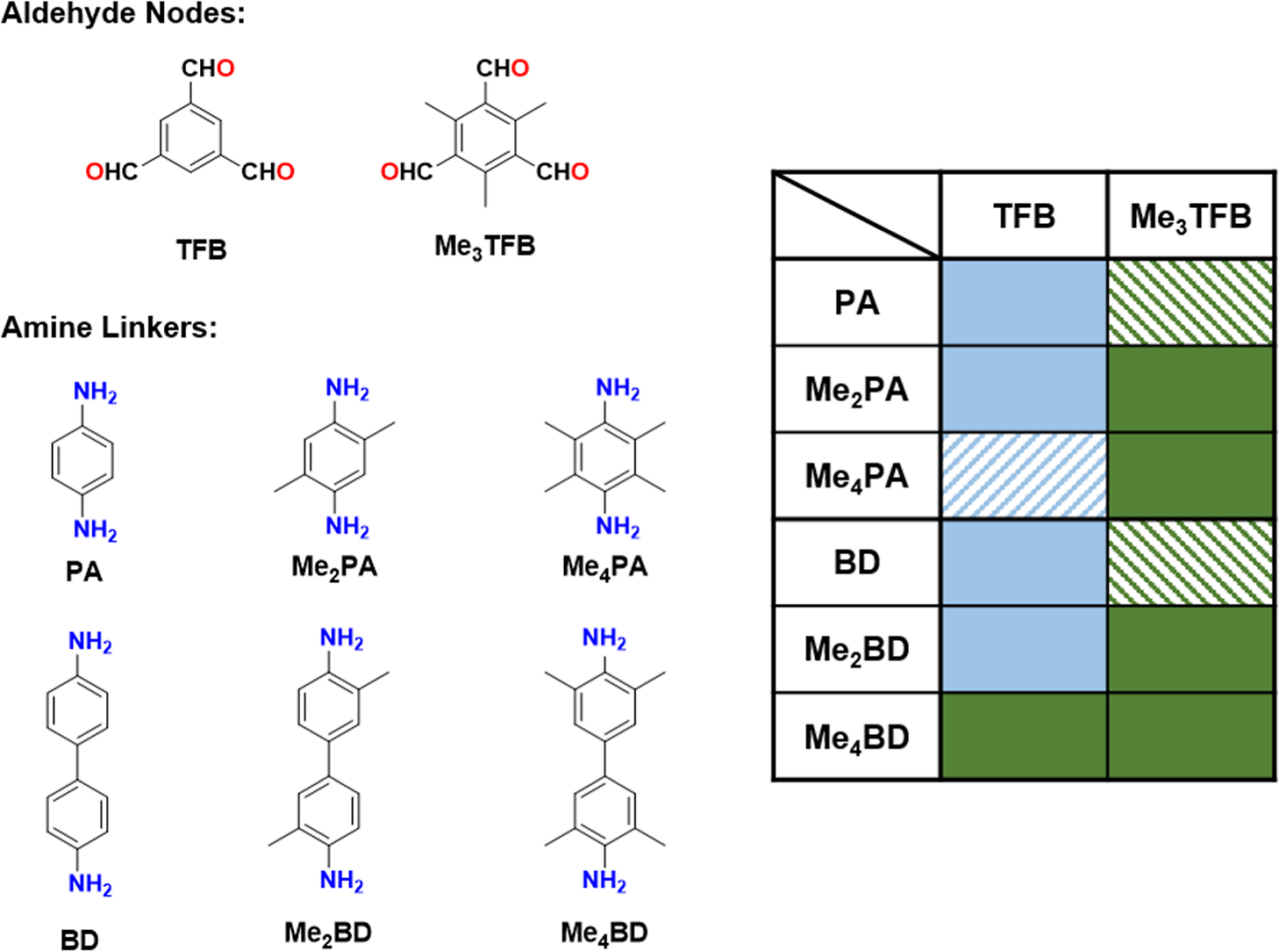
Structures of the aldehyde nodes (top left), amine linkers (bottom left), and matrix of the synthesized COFs (right)—plain blue: published in peer-reviewed literature before,^26,28−30^ blue diagonal stripes upward: synthesis reported in a patent and characterization data not shown,^27^ plain green: novel COFs, green diagonal stripes downward: published by our group.^16^

We synthesized a full range of COFs with (a) methylated (Me_3_TFB) and non-methylated (TFB) aldehyde nodes and (b) varying numbers of methyl groups on two different amine linkers (1,4-phenyleneamine derivatives: PA, Me_2_PA, and Me_4_PA and benzidine derivatives: BD, Me_2_BD, and Me_4_BD) whose structures are presented in Figure 1. Systematic combination of all building blocks leads to a series of 12 different COF materials, of which 5 have not been reported in literature to the best of our knowledge (Figure 1). We successfully synthesized five novel COFs: Me_3_TFB–Me_4_PA, Me_3_TFB–Me_2_BD, Me_3_TFB–Me_4_BD, TFB–Me_2_PA, and TFB–Me_4_BD. Keeping the synthetic and experimental conditions similar for all COFs within this and our previous study—in which we compared TFB–PA and TFB–BD to Me_3_TFB–PA and Me_3_TFB–BD for acid vapor sensing^16^—we also synthesized the COFs that have been published before (TFB–Me_2_PA,^26^ TFB–Me_4_PA,^27^ and TFB–Me_2_BD^28^), completing the matrix in Figure 1 and facilitating a direct comparison (Tables S1–S8).

### Synthesis and Characterization of COFs

In a classical Schiff base condensation reaction, 2 equiv of aldehyde node and 3 equiv of amine linker were reacted at 70 °C for 3 days in 1,4-dioxane:mesitylene 1:4 v/v at atmospheric pressure. While reactions with unmethylated amines were carried out with additional water to enhance the dynamic covalent chemistry (DCC), methyl-containing amines were reacted only with acetic acid acting as catalyst. The additional water can not only enhance error-correction via DCC, but in the case of methylated amines, the equilibrium was also shifted toward the starting materials as indicated by FT-IR spectroscopy, which showed a higher intensity for the C=O stretch of the aldehyde around ∼1690 cm^–1^. Furthermore, the BET surface area was found to be lower for COFs based on methyl-containing amines when additional water was added during the synthesis. After this reaction optimization, all COFs have been synthesized and characterized in triplicate. The COFs were isolated by Büchner filtration and subjected to an extensive washing procedure developed by Dichtel and Vitaku.^12^ The material was then divided into two batches for different COF activations (drying). One batch was dried at 120 °C overnight in an air-ventilated oven, and the other one was dried at 120 °C overnight in a vacuum oven.

First, based on new bands that appeared near 1620 cm^–1^ (Figures S1–S8), FT-IR spectroscopy confirmed the successful formation of imine bonds. All COF spectra show an attenuated C=O stretch of the aldehyde around ∼1690 cm^–1^, which is likely due to unreacted side groups at the outside of the 2D polymeric sheet.^15,31,32^ These relative intensities vary depending on the number of methyl groups attached to the amine linker. The more methyl groups are attached, the stronger is the residual aldehyde vibration in the spectrum. The repeatability for TFB–Me_4_PA is less good compared to the other COFs of this series. To shed light on the full COF series, this sample was still included in the further analysis. The lower repeatability is expressed by the larger error margins.

The crystallinity of the COFs was confirmed by PXRD analysis. The crystallinity and the BET surface areas of COFs are typically closely related, so both measurements were taken into account together for the characterization of the materials. All diffractograms, except for TFB–Me_4_BD, show diffraction peaks (Figures S9–S16). Some diffraction peaks are low in intensity and broad, but together with the BET surface area determination, we concluded that the obtained structures are COFs. However, based on its PXRD and BET characteristics, TFB–Me_4_BD is considered a COF-like porous organic polymer.

Pawley refinement was performed in triplicate, with the PXRD diffractogram using the space group *P*6/*m*, which corresponds to an eclipsed stacking structure. The unit cell dimensions and *R*_wp_ and *R*_p_ values are given in Table 1. For TFB–PA and TFB–BD, Pawley refinement was not performed because the stacking conformations have been published previously.^29,30,33^ For TFB–Me_2_BD, compared to literature, a larger interlayer stacking distance (3.73 ± 0.02 vs 3.4 Å) and lower *R*_wp_ and *R*_p_ values were obtained.^28^ For TFB–Me_4_PA, the refinement matched better to the space group *P*1 with lattice parameters of *a* = 22.80 ± 0.46 Å, *b* = 22.21 ± 0.26 Å, *c* = 6.74 ± 0.24 Å, α = 89.5 ± 1.21°, β = 91.1 ± 3.82°, and γ = 59.1 ± 1.10° with *R*_wp_ = 3.42 ± 1.24% and *R*_p_ = 2.23 ± 0.60%. For TFB–Me_4_BD, no Pawley refinement was conducted, because of the non-existing crystallinity. The Pawley-refined PXRD patterns were compared to the simulated diffraction patterns of optimized crystal structures obtained from DFT calculations (coordinates in Supporting Information). To compare experimental COF with simulated PXRD patterns, Zhang et al. suggested to use simulated patterns of statistical COF models, consisting of more than one single stacking order, to accurately model the PXRD diffractions of COFs.^34^ To obtain insights into the effect of stacking on the characteristics of PXRD patterns, the two extreme models “eclipsed” and “staggered” were simulated. Small local disorders were neglected to reduce computational complexity. Such small deviations in peak position and peak broadening can also be observed in our materials. It was therefore hypothesized that the small deviations in peak position between the experimental and the DFT-computed PXRD patterns are related to stacking disorder. All COFs—except for TFB–Me_4_PA—crystallize predominantly in an eclipsed stacking conformation including some stacking disorder. The deviations between the computed unit cell dimensions and the Pawley-refined values are below 5%, which is within the expected accuracy of the DFT calculations, further supporting the formation of eclipsed COFs. Only the deviation in interlayer stackings of Me_3_TFB–Me_4_BD is slightly higher (6.8%). The diffraction patterns (Figure S14) and unit cell dimensions of TFB–Me_4_PA exhibit a better match with the staggered model as already indicated by the different space group.

**Table 1:** Unit Cell Dimensions for All Uncharacterized COFs within This Article with the Space Group *P*6/*m*, both as Determined by Pawley Refinement and as Computed by DFT Calculations

	Pawley-refined unit cell dimensions				computed unit cell dimensions	
COF	*a* = *b* [Å]	*c* [Å]	*R*_wp_ [%]	*R*_p_ [%]	*a* = *b* [Å]	*c* [Å]
TFB–Me_2_PA	23.54 ± 0.66	3.76 ± 0.06	2.13 ± 0.81	1.48 ± 0.55	22.69	3.78
Me_3_TFB–PA	21.96 ± 0.07	3.77 ± 0.04	5.77 ± 2.57	3.97 ± 1.67	22.52	3.75
Me_3_TFB–Me_2_PA	21.87 ± 0.12	3.73 ± 0.01	4.08 ± 0.81	2.79 ± 0.51	22.56	3.80
Me_3_TFB–Me_4_PA	23.17 ± 0.21	3.73 ± 0.02	1.56 ± 0.61	1.16 ± 0.41	22.69	3.78
TFB–Me_2_BD	30.35 ± 0.29	3.73 ± 0.02	2.92 ± 3.16	2.09 ± 2.13	29.95	3.74
TFB–Me_4_BD	not crystalline				30.24	3.80
Me_3_TFB–BD	29.64 ± 0.11	3.67 ± 0.11	6.79 ± 0.56	4.74 ± 0.46	30.01	3.72
Me_3_TFB–Me_2_BD	29.86 ± 0.55	3.65 ± 0.08	2.04 ± 1.33	1.51 ± 0.90	30.02	3.72
Me_3_TFB–Me_4_BD	28.98 ± 0.74	3.52 ± 0.13	0.70 ± 0.08	0.56 ± 0.06	30.12	3.78

Upon increasing the number of methyl groups in the amine linker, the crystallinity decreases. This can be rationalized by a less reactive tetramethyldiamine due to steric hindrance. The more unreacted aldehyde groups remain at the outside of the 2D polymeric sheets, the higher is the intensity of the C=O stretch in the FT-IR spectra. For aldehyde nodes, the crystallinity is larger when using methylated building blocks.

To study the COF stability in water, 1 M NaOH, 1 M hydrochloric acid, dichloromethane (DCM), and *N*,*N*-dimethylformamid (DMF), COFs were immersed for 5 days into the respective solvents, and upon re-isolation and drying at 120 °C overnight, the PXRD patterns were recorded again. To investigate the chemical stability of the novel COFs, representative samples (methylated aldehyde, fully methylated amine, and different amines) were chosen: Me_3_TFB–PA, TFB–Me_4_PA, Me_3_TFB–Me_4_PA, and Me_3_TFB–BD (Figure S17). Wang et al.^35^ and Daugherty et al.^36^ already studied the chemical stability of TFB–PA and TFB–BD, respectively. Me_3_TFB–PA, TFB–Me_4_PA, Me_3_TFB–Me_4_PA, and Me_3_TFB–BD were found to be stable in 1 M NaOH and DMF. All tested COFs are chemically stable in DCM except for Me_3_TFB–BD, which is only partially stable in DCM. All COFs are stable in water, except for Me_3_TFB–Me_4_PA. The instability of Me_3_TFB–Me_4_PA in water is surprising, because the high number of hydrophobic methyl groups was anticipated to prevent water from a nucleophilic attack.

The thermal stability was determined by thermogravimetric analysis (TGA); Me_3_TFB–Me_4_PA and TFB–Me_4_PA started to decompose at 363 and 412 °C, respectively (Figure S65). This is comparable to the thermal stability of Me_3_TFB–PA and Me_3_TFB–BD.^16^ It is anticipated that the stability of the other COFs in this series will be similar.

### ssNMR Assignment

**Figure 2:**
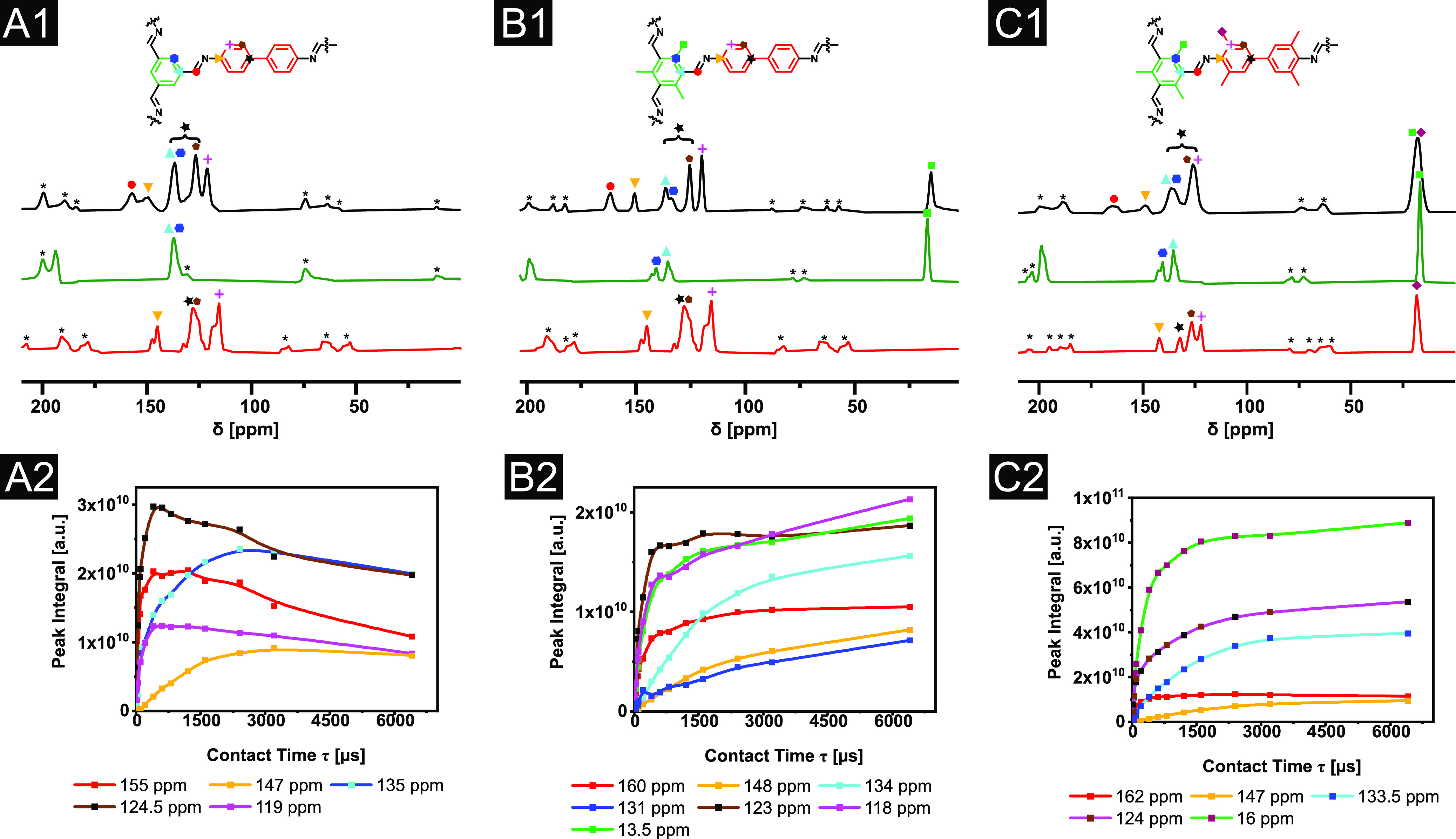
Solid-state ^13^C CPMAS NMR spectra of respective COFs at a spinning frequency of 11 kHz at 3.2 ms CP contact time (black): (A) TFB–BD, (B) Me_3_TFB–BD, and (C) Me_3_TFB–Me_4_BD aldehyde node at 3.0 ms CP contact time (green) and amine linker at 3.0 ms CP contact time (red) in the top (A1–C1) and CP build-up curves in the bottom (A2–C2). Spinning side bands are denoted with an asterisk.

^13^C cross-polarization magic angle spinning ssNMR (^13^C CPMAS ssNMR) spectra were recorded to investigate the structure of the COFs. We used two different spinning frequencies to distinguish COF signals from spinning side bands (Supporting Information, section “ssNMR”). Compared to the spectra of the starting materials, the ^13^C CPMAS NMR spectra of COFs show a strongly attenuated signal around 190–200 ppm, characteristic for aldehyde carbons, and a signal around 160 ppm, which can be assigned to the imine carbons (Figure 2A1–C1). These observations lead to the conclusion that imine-linked COFs have indeed been formed. Based on the ^13^C CPMAS NMR spectra, structural assignments for COFs have been proposed.^29,30^

As a next step, CP contact time-dependent CPMAS NMR analysis provides added value for an evidence-based assignment of such materials.^37^ By varying the CP contact time and recording the respective spectra, the ssNMR signals of differently substituted carbon atoms show a different relative cross-polarization build-up, which enables the assignment of the signals to the carbons present in the COF structure. This is a facile and straightforward strategy for structure elucidation of solid materials in general and has been only recently employed in COF characterization. Acquiring 2D HETCOR ssNMR spectra at two different CP contact times enabled Samori and co-workers to fully assign the ^13^C spectrum of TFB–PA.^37^ To acquire 2D HETCOR spectra, an ssNMR probe with a spinning rate as high as 60 Hz is required and such a special probe is not widely available. Recently, Zhu et al.^38^ have assigned the ^13^C spectrum of a methoxy-functionalized, imine-linked COF based on 15 different CP contact times by comparing the relative CP data, which were obtained at lower spinning rates. We built further on this approach, expanded it to a more complex COF structure, and also analyzed the ssNMR spectra with the open-source software ssNnake^18^ to obtain the CP build-up curves. The advantage of CP build-up curves over measuring at two CP contact times is that they directly visualize the rate of each signal build-up. Based on these curves, the required CP contact times for structure elucidation can be identified and selected to save measurement time for future structural assignments of similar COFs.

The assignment of the NMR spectra of the starting materials in Figure 2A1–C1 has been done by liquid NMR measurements (^13^C and ^135^DEPT), before double-checking whether the signals appear at approximately the same chemical shifts in ssNMR. At first, we applied our CP contact time-dependent analysis to assign TFB–PA with the help of Me_3_TFB–PA and TFB–Me_4_PA (Figure S18). As the methylated COFs are derivatives of the unmethylated COFs, and a methyl substituent typically has only a small effect on arylic chemical shifts, we expect comparable chemical shifts, enabling one to assign all NMR signals by comparing the spectra and CP build-up curves (Figure 2A2–C2). We found for both combinations (TFB–PA/Me_3_TFB–PA and TFB–PA/TFB–Me_4_PA) that the assignment matches the one measured with 2D HETCOR NMR.^37^ We now implemented this method in its full potential to our extended series, including TFB–BD and its methyl derivatives. TFB–BD has also been assigned previously, though no experimental support was shown.^29^ Hence, we measured the CP build-up for TFB–BD (Figure 2A2), Me_3_TFB–BD (Figure 2B2), and Me_3_TFB–Me_4_BD (Figure 2C2). Each COF is symmetrical, and in this sequence, each COF has one quaternary carbon more than the previous one. It is noteworthy that the crystallinity of the COF is also reflected by the width of the signal. The sterically demanding Me_3_TFB–Me_4_BD has a lower crystallinity and thus more signals that overlap in the spectrum. The best resolution was obtained for Me_3_TFB–BD (Figure 2B1), which therefore was taken as a starting point for the assignment. The eight different carbon atoms in Me_3_TFB–BD—as labeled in the chemical structure depicted in Figure 2B1—yield seven separate signals, indicating that two carbons have an identical chemical shift. First, the methyl group could be assigned to the signal at 15 ppm and the imine carbon to the signal at 160 ppm, both based on their chemical shift. For the next step in the assignment, it is our experience that it is best to first look at the spectrum of TFB–BD (Figure 2A1). It shows only one slow CP build-up (Figure 2A2), which means that out of the three quaternary carbons, two are overlapping with a tertiary carbon. Based on the chemical shift, the carbon is deshielded, indicating an electronegative atom in its surrounding. In addition, the quaternary carbon from the amine linker could be assigned to ∼148 ppm in the spectrum of TFB–PA, supporting the same assignment for TFB–BD, Me_3_TFB–BD, and Me_3_TFB–Me_4_BD. The signals at 134 ppm and at 131 ppm (Figure 2B1) can be assigned to the aryl carbons from the aldehyde node based on the chemical shifts of the starting materials. In the spectra of TFB–BD (Figure 2A1) and Me_3_TFB–Me_4_BD (Figure 2C1), these two signals overlap, which is also supported by the relatively fast CP build-up for TFB–BD (Figure 2A2) due to the tertiary aryl carbon and the slow build-up after methylation when both aryl carbons are quaternary. We assume that the signal at 134 ppm belongs to the carbon next to the imine carbon based on the assignment found for TFB–PA. By now, only three carbon atoms are not assigned yet. Based on the starting material, we hypothesize that the signal at 123 ppm belongs to the meta position, and the signal at 118 ppm belongs to the ortho position with respect to the former amine. The carbon atom, which links the two phenyl rings in benzidine, cannot be assigned to a specific signal. Its signal seems to overlap with other signals, and based on the chemical shift in the starting material, it can either overlap with the carbons from the aldehyde node or with the carbons from the benzidine. The same order for the assignment was applied to TFB–BD and Me_3_TFB–Me_4_BD. The ssNMR spectra for different CP contact times and the spectra of the other COFs can be found in the Supporting Information, section “ssNMR”.

### BET Surface Area Dependencies

**Figure 3:**
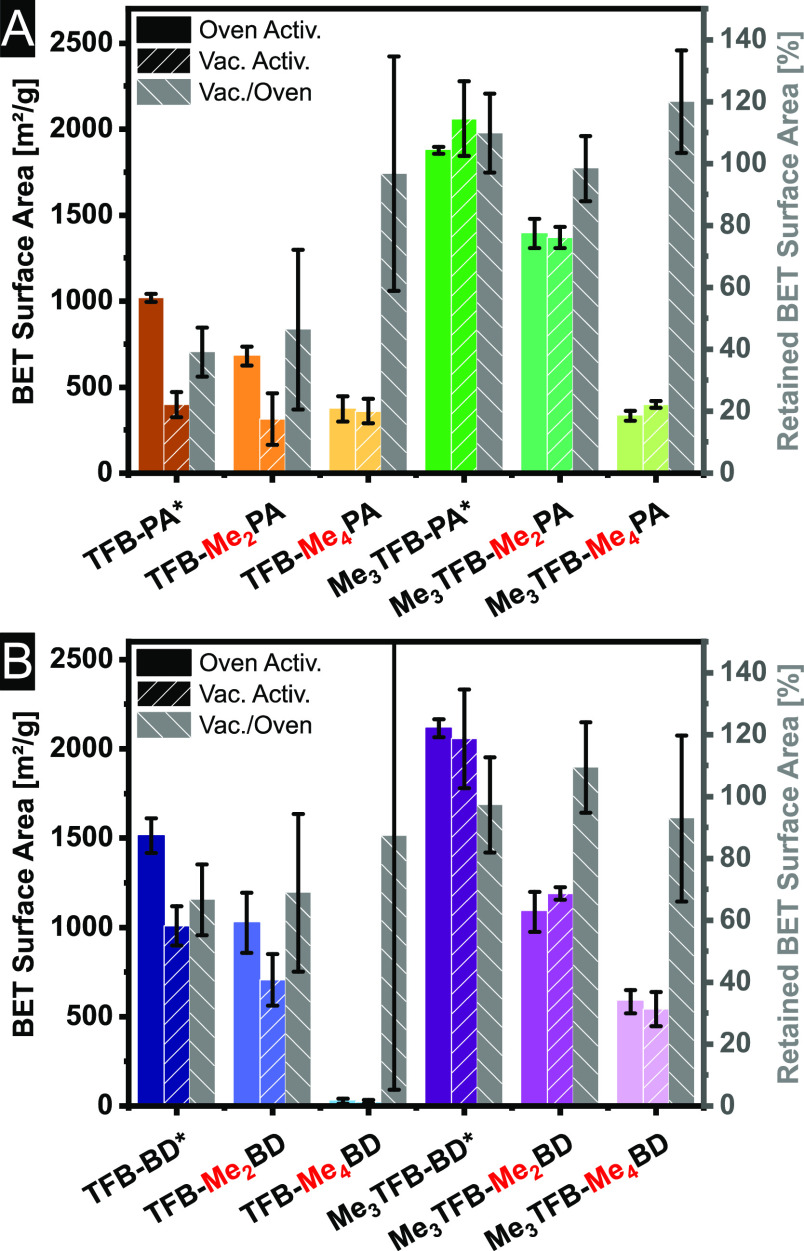
BET surface areas and retained BET surface areas for PA-COFs (A) and BD-COFs (B) after oven and vacuum activation. The retained BET surface area gives the ratio of how much *S*_BET_ has been left after vacuum drying. The errors are based on the analyses of three batches. The asterisk indicates already published data, which is shown to illustrate the trends better.

Nitrogen sorption experiments have been carried out to determine the porosity and BET surface area of all COFs. All samples were measured in triplicate to check the repeatability. The isotherms can be found in Figures S43–S50. The adsorption branches were used to calculate the BET surface area (*S*_BET_). All samples are microporous, which means that the original *p*/*p*^0^ range from 0.05 to 0.3 is not applicable. Therefore, lower relative pressures have been used for the linear fit, and the Rouquerol criteria^39^ were applied. The same pressure range has been used for the linear fit of the same COF. The BET surface areas for all COFs and both activation methods are displayed in Figure 3A,B. The BET surface areas vary over a very broad range from 28 to 2215 m^2^/g. For TFB–PA,^36^ TFB–Me_2_PA,^26^ TFB–BD,^40^ and TFB–Me_2_BD,^28^*S*_BET_ has been reported before, and the highest reported values are 1571, 144, 1948, and 821 m^2^/g for these COFs, respectively. For TFB–Me_4_PA, which is reported in a patent, we could not find any *S*_BET_ value. However, TFB–Me_2_PA was measured to have a *S*_BET_ of 680 ± 55 m^2^/g and TFB–Me_2_BD of 1026 ± 168 m^2^/g, which are, to the best of our knowledge, the highest reported values so far for these specific COFs. For TFB–BD, the BET surface area was found to be 1514 ± 97 m^2^/g. The reported value in the literature is approximately 29% higher, which is likely due to the supercritical CO_2_ drying. This is a mild activation method that prevents pore collapse. The exact values of all BET surface areas can be found in Figures S43–S50.

We have recently reported that the BET surface area increases due to methylation of the aldehyde for Me_3_TFB–PA and Me_3_TFB–BD.^16^Figure 3A,B shows that methylation of the aldehyde leads to an increase in the surface area for five out of the six pairs synthesized in this study. The only exception is TFB–Me_4_PA/Me_3_TFB–Me_4_PA, for which the BET surface areas are approximately the same. It must be noted that the synthesis of TFB–Me_4_PA is challenging and shows a low repeatability. The increase in *S*_BET_ for aldehyde-methylated COFs is in line with the crystallinity of the COFs. The magnitude of this increase varies for the different COFs. The expansion of this previous results implies an overall trend of increasing BET surface area by employing a methylated aldehyde node. It is, however, important which building block is methylated, because *S*_BET_ decreases when the amine linker is methylated. In Figure 3A,B the BET surface area continuously decreases upon attaching more methyl groups ortho to the amino group. This trend is visible for all four COF series (TFB–PA, Me_3_TFB–PA, TFB–BD, and Me_3_TFB–BD), in which we added increasingly more methyl groups. These findings are in line with a higher C=O aldehyde to C=N imine stretch ratio in FT-IR spectra for non-methylated COFs compared to their methylated equivalents. This leads us to the conclusion that the position of the methyl group is crucial for the porosity of the COFs. We hypothesize that the steric demand of the methyl group plays a role in the COF formation and that this affects the COF formation in two ways. As reported previously,^16^ the methyl groups reduce the conjugation of the carbonyl groups with the π-system of the aromatic Me_3_TFB ring, leading to a smaller rotational barrier after methylation. The smaller barrier enhances the reaction rate of the conformational change by several magnitudes, and the preferred orientation of the aldehyde node for the framework formation is achieved faster, which results in a more regular framework. On the other hand, when the steric demand is present on the amine linker, the nucleophilic attack of the amine group at the carbonyl group can be already more difficult and is less likely to happen, which leads to the observed decrease in the BET surface area. This line of thought fits with the restricted synthesis based on steric hindrance as discussed based on the FT-IR and PXRD patterns.

Not only the structure but also the activation method plays a crucial role in the porosity of COFs. To investigate the stability of the COFs in this study, all COFs were activated at 120 °C in the two different ways that we mentioned before: vacuum versus ambient pressure. Afterward, the BET surface area was measured for all samples, and to correct for the difference in BET surface area due to different pore sizes, the retained BET surface area has been calculated (Eq 1).

Again, the methylated aldehyde nodes lead to an increase in retained *S*_BET_, meaning that the frameworks are less prone to pore collapse. The magnitude of this effect varies from COF to COF, being the smallest for TFB–Me_4_BD/Me_3_TFB–Me_4_BD with only 5% difference and the largest with 70% difference for TFB–PA/Me_3_TFB–PA. For most methylated COFs also the standard deviations are lower than the ones for the non-methylated aldehyde COFs with the exception of TFB–PA/Me_3_TFB–PA and TFB–BD/Me_3_TFB–BD which have roughly the same standard deviations. Based on these findings, Me_3_TFB can reduce pore collapse and lead to robust COFs.

### UV Absorbance

**Figure 4:**
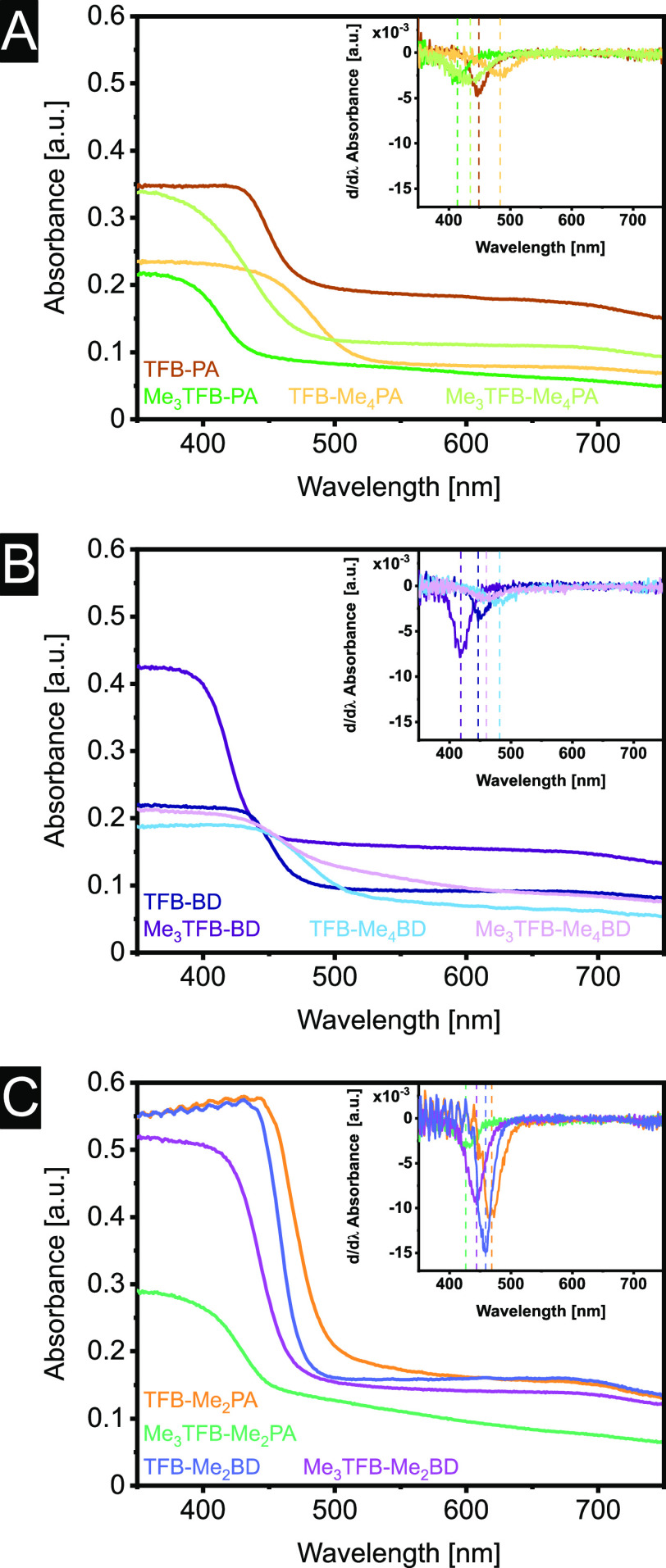
Absorbance spectra of (A) TFB–PA, TFB–Me_4_PA, Me_3_TFB–PA, and Me_3_TFB–Me_4_PA; (B) TFB–BD, TFB–Me_4_BD, Me_3_TFB–BD, and Me_3_TFB–Me_4_BD; and (C) TFB–Me_2_PA, Me_3_TFB–Me_2_PA, TFB–Me_2_BD, and Me_3_TFB–Me_2_BD and their first derivatives as insets for a better comparison.

The UV absorbance of the COFs was determined with diffuse reflectance spectroscopy. In Figure 4, the absorbance spectra are displayed for the different COFs, and the first derivatives of these plots are given in the insets. The absorption edges of all COFs are within 370–450 nm (Table 2), thus in the purple and blue region of the visible spectrum, explaining the yellow color of all samples. The intensity depends on the thickness of the sample film, but the shape of the curve does not change. However, it is observed that sometimes the plateau is reached relatively fast, whereas for other samples, the curve changes only slowly before reaching the plateau. As the shape of the spectra does not depend on the amount of sample, we believe that the slope of the curve is also characteristic of a specific material, supporting the use of the inflection point as a new data analyses approach.

**Table 2:** Overview of Absorption Edges, Inflection Points, and the Calculated Band Gaps for all COFs

COF	estimated absorption edge [nm]	derived inflection point [nm]	derived optical band gap^a^[eV]
TFB–PA	429	449	2.70
TFB–Me_2_PA	448	469	2.64
TFB–Me_4_PA	438	484	2.52
Me_3_TFB–PA	377	414	2.94
Me_3_TFB–Me_2_PA	380	427	2.83
Me_3_TFB–Me_4_PA	370	435	2.85
TFB–BD	435	447	2.70
TFB–Me_2_BD	441	459	2.72
TFB–Me_4_BD	432	482	2.54
Me_3_TFB–BD	390	419	2.93
Me_3_TFB–Me_2_BD	410	444	2.80
Me_3_TFB–Me_4_BD	435	460	2.56

a Based on Tauc plot analysis.

Hence, we used Origin to calculate the first derivative and used the inflection point for additional information. Data analysis with this approach shows that for COFs with a methylated amine linker, the absorbance spectra red-shifts, whereas a blue shift is observed for methylated aldehyde nodes (Figure 4A–C). Methylation therefore gives a handle to tune the UV absorbance of COFs. Methylation of both aldehyde node and amine linker results in an inflection point in between the COFs, with only one methylated building block. By increasing the number of methyl groups on the amine linker, the bathochromic shift of the absorbance spectrum gradually becomes more pronounced (Figure S51).

Traditional analysis of the absorption edge leads to the same findings with one exception: the trend in which the spectra red shifts based on the number of methyl groups does then not apply anymore. Additionally, from Me_3_TFB–PA to Me_3_TFB–Me_4_PA, the absorption edge does not increase, but for the other three COF series, this trend still applies. These findings further strengthen our mathematical approach to analyze diffuse reflectance spectra by comparing their inflection points.

All changes in absorbance are also visible by eye, because the isolated COF powders have different shades of yellow (Figure S52). All the trends we found lead to the possibility of pre-designing the UV absorbance based on the choice of building blocks.

The bathochromic shift upon methylation of the amine linker can be explained by the inductive, electron-donating effect of methyl groups. The hypsochromic shift for methylated aldehydes can, however, not be explained by this effect. The DFT structure optimization of the COF sheets (Figures S66–S68) shows that the Me_3_TFB–COF structures are bended, while the TFB–COF structures are flat. Such a difference was not found upon the methylation of the amine linker. We hypothesize that the observed bending from the methylated aldehyde node decreases the delocalization of the entire conjugated system, which would result in an increased excitation energy, leading to a blue shift.

Fundamental knowledge on how the UV absorbance and the optical band gap are influenced by its COF structure is of importance to further develop photocatalytic applications. To study the effect of methyl groups on the optical band gaps, we converted our absorbance spectra into the Kubelka–Munk function,^41^ assuming a direct allowed transition. Plotting these data in an adapted Tauc plot^42^ enables one to obtain the optical band gap (Figures S53–S64). The optical band gaps for TFB–COFs are in the range of 2.72–2.52 eV, and methylated aldehyde nodes result in slightly higher band gaps of 2.94–2.56 eV. This result matches with the observed blue shift upon methylation of the aldehyde node. Upon tetramethylation of the amine linker, the optical band gap decreases. The energies of the band gaps as well as the observed trends show a better match with the analysis of the UV spectra via their inflection points compared to the traditional analysis via the absorption edge. This supports again that diffuse reflectance spectra with S-shaped curves can be analyzed by their inflection point. Overall, the band gaps do not change considerably, which means that methylation can be used to fine-tune the UV absorbance toward the desired application while getting a predictable porosity.

## Conclusions

Multiple combinations of non-methylated (TFB) and methylated (Me_3_TFB) aldehyde nodes with either methylated or non-methylated *p*-phenylenediamine or benzidine linkers have been synthesized, and imine COF formation was proved by FT-IR spectroscopy, BET surface area analysis, and PXRD measurements. Pawley refinement yielded the unit cell dimensions, which were compared to the computationally modeled structures. For the first time, the signals of the ssNMR spectra of TFB–BD, Me_3_TFB–BD, and Me_3_TFB–Me_4_BD could experimentally be assigned using contact time-dependent CPMAS NMR and comparing the different COF derivatives. An in-depth study of the COF series revealed several trends in their properties based on the methyl substitution pattern of their corresponding building blocks. First, COFs with Me_3_TFB show larger crystallinity and therefore increased BET surface areas up to approximately 2100 m^2^/g. In addition, the COFs are less prone to pore collapse, which was proven by comparing vacuum activation with drying in a regular oven. Second, it is of importance which building block is methylated (aldehyde vs amine), since—in contrast to their aldehyde counterparts—COFs with methylated amine linkers show a decrease in crystallinity and BET surface area. This is likely attributed to the higher steric demand of the methyl groups, which is supported by the third trend: the more methyl groups are attached to the amine linker, the stronger are the decreases in crystallinity and BET surface area. In addition to the steric influence on the conjugation of the carbonyl groups with the aromatic ring, we hypothesize that the methyl groups on the amine linker sterically hinder the COF formation. An amine methylation does not show a significant effect on the robustness of the COF against vacuum activation, which is again in contrast to their aldehyde counterparts. Therefore, the position of functionalization within the COF building blocks is crucial.

Fourth, the absorbance spectra blue-shifts if Me_3_TFB is used as a building block, which leads to a small increase in the optical band gap compared to TFB–COFs. We hypothesize that this is caused by the decreased conjugation caused by the methyl groups. COFs with methyl groups on the amine linkers show a red shift of the absorbance and smaller optical band gaps, which is in line with the bathochromic effect expected from methyl groups.

To conclude, the effect of methyl substitution patterns on high surface area COF properties can serve for an enhanced rational design of COFs. Furthermore, it allows one to fine-tune the optical band gaps, which can be relevant for photocatalytic applications of COFs.

## Supporting Information Available

Synthetic procedures, FT-IR spectra, PXRD diffractograms (experimental, Pawley refined, modelled, and chemical stability), ^13^C CPMAS NMR spectra, nitrogen sorption isotherms and pore size distributions, optical properties, Tauc plots, thermogravimetric analysis (TGA), modeled COF structures (by DFT) (ZIP)
Synthetic procedures, FT-IR spectra, powder X-ray diffraction analysis, ^13^C CPMAS NMR spectra, nitrogen sorption analysis, optical properties, Tauc plots, thermogravimetric analysis, and DFT calculations (PDF)
