# Supplementary material for: Tuning UV Absorption in Imine-Linked Covalent Organic Frameworks via Methylation
Source: J Phys Chem C Nanomater Interfaces. 2022 Nov 24;126(50):21338–47. doi: 10.1021/acs.jpcc.2c04586 (PMC9791660; doi:10.1021/acs.jpcc.2c04586)
Supplement: Supplementary file 2 — jp2c04586_si_002.pdf [file jp2c04586_si_002.pdf]

SUPPORTING INFORMATION

Tuning UV Absorption in Imine-Linked Covalent Organic Frameworks (COFs) via Methylation

*Ellen Dautzenberg[1], Milena Lam[1], Tatiana Nikolaeva[2], Wouter M. J. Franssen[3], Barend van Lagen[1], Ilse P.A.M. Gerrits-Benneheij,[4] Nikolay A. Kosinov,[5] Guanna Li[1,6], Louis C. P. M. de Smet[1]**

[1] Laboratory of Organic Chemistry, Wageningen University, Stippeneng 4, 6708WE Wageningen, The Netherlands

[2] MAGNEtic resonance research FacilitY-MAGNEFY, Wageningen University, Stippeneng 4, 6708WE, Wageningen, The Netherlands

[3] Laboratory of Biophysics, Wageningen University, Stippeneng 4, 6708WE Wageningen, The Netherlands

[4] Environmental Technology, Wageningen University, Bornse Weilanden 9, 6708WG Wageningen, The Netherlands

[5] Laboratory of Inorganic Materials and Catalysis, Department of Chemical Engineering and Chemistry, Eindhoven University of Technology, P.O. Box 513, 5600 MB Eindhoven, The Netherlands

[6] Biobased Chemistry and Technology, Wageningen University, Bornse Weilanden 9, 6708WG Wageningen, The Netherlands

Contact details: louis.desmet@wur.nl (Louis C.P.M. de Smet*, +31-317481268).

Author Contributions: E.D and L.C.P.M.d.S. conceived the idea and designed the experiments. E.D. and M.L performed the experiments. B.v.L. performed the PXRD measurements. E.D., T.N. and W.M.J.F. performed the ssNMR measurements. G.L. performed the modelling. E.D, M.L., I.P.A.M.G.-B., N.K. and L.C.P.M.d.S. analyzed the data. L.C.P.M.d.S. secured funding and guided the project. E.D. prepared a first draft of the manuscript and all authors gave input.

Contents

A. Synthetic Procedures 4

B. FT-IR Spectra 12

C. Powder X-Ray Diffraction Analysis 20

D. ^{13}C CPMAS NMR Spectra 27

E. Nitrogen Sorption Analysis 41

F. Optical Properties 49

G. Tauc plots 51

H. Thermogravimetric analysis (TGA) 54

I. DFT calculations 55

J. References 57

A. Synthetic Procedures

Synthesis of Me_3TFB-Me_2PA

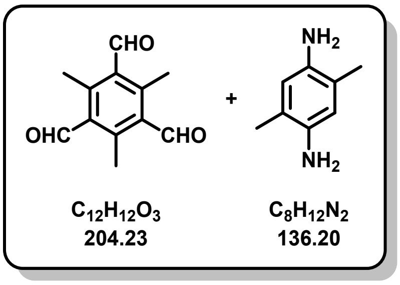

Table S1. The yield and the amount of monomers, solvent, water and acetic acid used for the syntheses of Me_3TFB Me_2PA, as well as the average BET surface areas of air and vacuum activated COFs.

	**Scale 1**
**Monomer mass aldehyde**	135 mg, 0.661 mmol (2 equiv.)
**Equivalent amine**	3
**1,4-Dioxane:mesitylene 4:1 *v/v* [mL]**	5
**H_2O**	/
**Glacial acetic acid**	1.9 mL<br>50 equiv.
**Yield [%]**	87
**Average air-activated BET surface area [m²/g]**	1393±86
**Average vacuum-activated BET surface area [m²/g]**	1371±62

Synthesis of Me_3TFB-Me_4PA

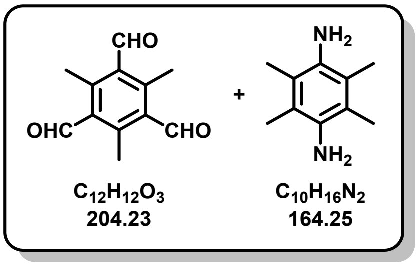

Table S2. The yield and the amount of monomers, solvent, water and acetic acid used for the syntheses of Me_3TFB Me_4PA, as well as the average BET surface areas of air and vacuum activated COFs.

	**Scale 1**	**Scale 2**
**Monomer mass aldehyde**	135 mg, 0.661 mmol (2 equiv.)	270 mg, 1.322 mmol (2 equiv.)
**Equivalent amine**	3	3
**1,4-Dioxane:mesitylene 4:1 *v/v* [mL]**	5	10
**H_2O**	/	/
**Glacial acetic acid**	1.6 mL<br>42 equiv.	3.2 mL<br>42 equiv.
**Yield [%]**	79	42
**Average air-activated BET surface area [m²/g]**	333±29	
**Average vacuum-activated BET surface area [m²/g]**	400±20	

Synthesis of Me_3TFB-Me_2BD

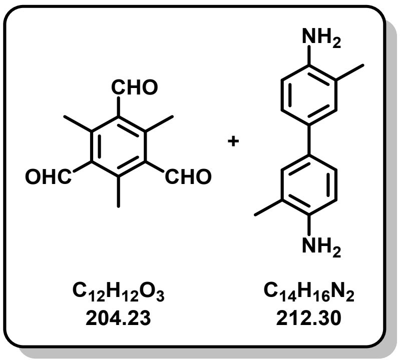

Table S3. The yield and the amount of monomers, solvent, water and acetic acid used for the syntheses of Me_3TFB Me_2BD, as well as the average BET surface areas of air and vacuum activated COFs.

	**Scale 1**
**Monomer mass aldehyde**	135 mg, 0.661 mmol (2 equiv.)
**Equivalent amine**	3
**1,4-Dioxane:mesitylene 4:1 *v/v* [mL]**	5
**H_2O**	/
**Glacial acetic acid**	1.9 mL<br>50 equiv.
**Yield [%]**	85
**Average air-activated BET surface area [m²/g]**	1088±112
**Average vacuum-activated BET surface area [m²/g]**	1190±36

Synthesis of Me_3TFB-Me_4BD

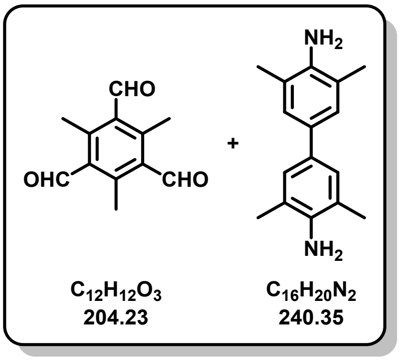

Table S4. The yield and the amount of monomers, solvent, water and acetic acid used for the syntheses of Me_3TFB Me_4BD, as well as the average BET surface areas of air and vacuum activated COFs.

	**Scale 1**
**Monomer mass aldehyde**	135 mg, 0.661 mmol (2 equiv.)
**Equivalent amine**	3
**1,4-Dioxane:mesitylene 4:1 *v/v* [mL]**	5
**H_2O**	/
**Glacial acetic acid**	1.6 mL<br>42 equiv.
**Yield [%]**	80
**Average air-activated BET surface area [m²/g]**	584±66
**Average vacuum-activated BET surface area [m²/g]**	543±95

TFB-Me$_2$PA

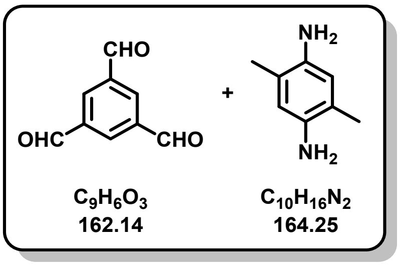

Table S5. The yield and the amount of monomers, solvent, water and acetic acid used for the syntheses of TFB Me$_2$PA, as well as the average BET surface areas of air and vacuum activated COFs.

	**Scale 1**	**Scale 2**
**Monomer mass aldehyde**	125 mg, 0.771 mmol (2 equiv.)	250 mg, 1.542 mmol, (2 equiv.)
**Equivalent amine**	3	3
**1,4-Dioxane:mesitylene 4:1 *v/v* [mL]**	5	10
**H_2O**	/	/
**Glacial acetic acid**	1.8 mL<br>41 equiv.	3.6 mL<br>41 equiv.
**Yield [%]**	65	92
**Average air-activated BET surface area [m²/g]**	680±55	
**Average vacuum-activated BET surface area [m²/g]**	315±150	

TFB-Me$_4$PA

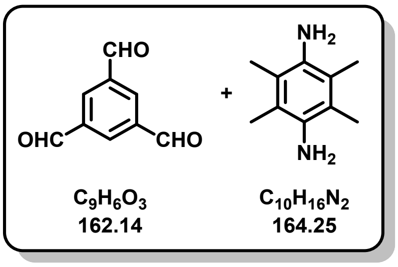

Table S6. The yield and the amount of monomers, solvent, water and acetic acid used for the syntheses of TFB Me$_4$PA, as well as the average BET surface areas of air and vacuum activated COFs.

	**Scale 1**
**Monomer mass aldehyde**	125 mg, 0.771 mmol (2 equiv.)
**Equivalent amine**	3
**1,4-Dioxane:mesitylene 4:1 *v/v* [mL]**	5
**H_2O**	1.2 mL<br>87 equiv.
**Glacial acetic acid**	1.6 mL<br>36 equiv.
**Yield [%]**	65
**Average air-activated BET surface area [m²/g]**	373±74
**Average vacuum-activated BET surface area [m²/g]**	361±70

** Instead of 70 ˚C as described in the general procedure, a temperature of 120 ˚C was used during the 3-day reaction step.*

TFB-Me_2BD

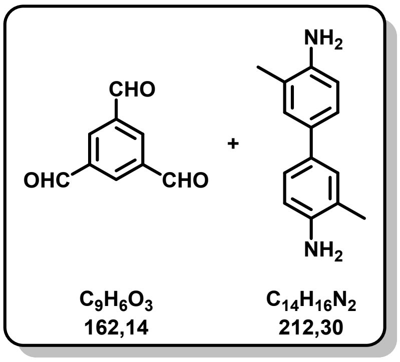

Table S7. The yield and the amount of monomers, solvent, water and acetic acid used for the syntheses of TFB Me_2BD, as well as the average BET surface areas of air and vacuum activated COFs.

	**Scale 1**	**Scale 2**
**Monomer mass aldehyde**	125 mg, 0.771 mmol (2 equiv.)	250 mg, 1.542 mmol, (2 equiv.)
**Equivalent amine**	3	3
**1,4-Dioxane:mesitylene 4:1 *v/v* [mL]**	5	10
**H_2O**	/	/
**Glacial acetic acid**	1.8 mL<br>41 equiv.	3.6 mL<br>41 equiv.
**Yield [%]**	74	87
**Average air-activated BET surface area [m²/g]**	1026±168	
**Average vacuum-activated BET surface area [m²/g]**	707±144	

TFB-Me$_4$BD

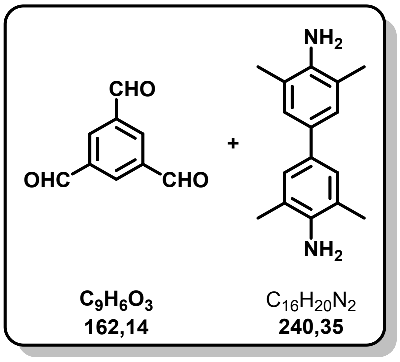

Table S8. The yield and the amount of monomers, solvent, water and acetic acid used for the syntheses of TFB Me$_4$BD, as well as the average BET surface areas of air and vacuum activated COFs.

	**Scale 1**	**Scale 2**
**Monomer mass aldehyde**	125 mg, 0.771 mmol (2 equiv.)	250 mg, 1.542 mmol, (2 equiv.)
**Equivalent amine**	3	3
**1,4-Dioxane:mesitylene 4:1 *v/v* [mL]**	5	10
**H_2O**	/	/
**Glacial acetic acid**	1.8 mL<br>41 equiv.	3.6 mL<br>41 equiv.
**Yield [%]**	70	79
**Average air-activated BET surface area [m²/g]**	26.6±13.6	
**Average vacuum-activated BET surface area [m²/g]**	23.1±10.0	

B. FT-IR Spectra

Me_3TFB-Me_2PA

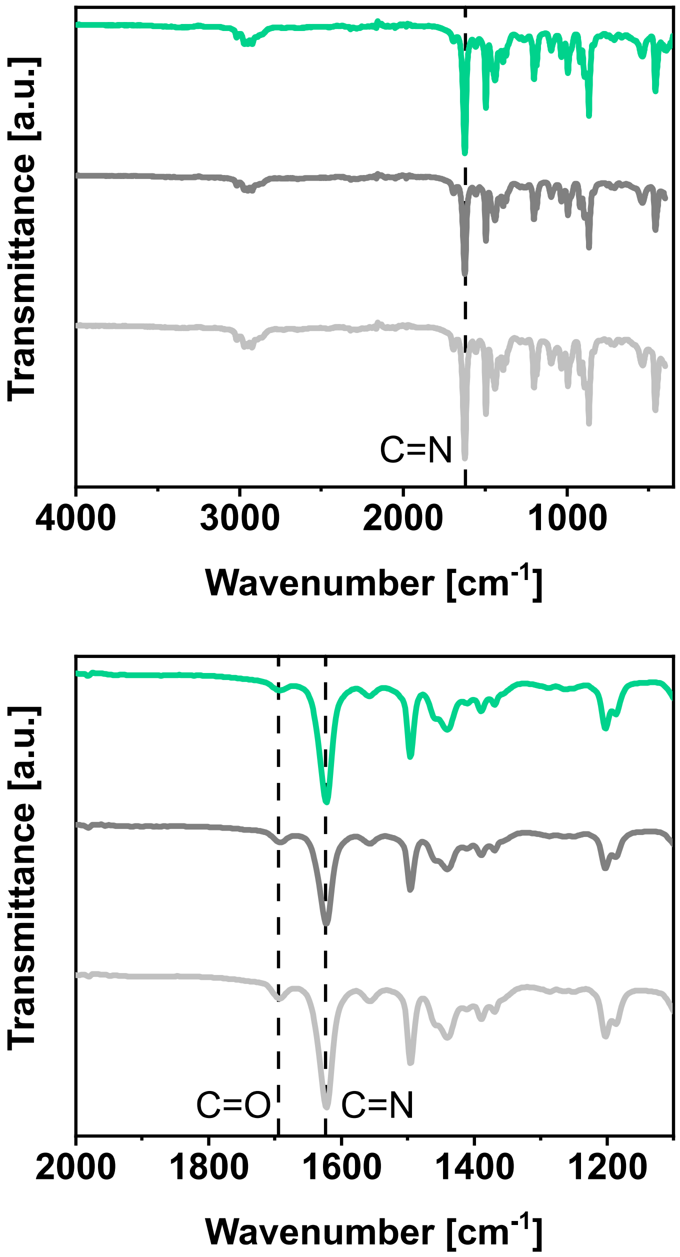

Figure S1. FT-IR spectra of triplicate Me_3TFB-Me_2PA COFs to show the repeatability of the synthesis. The C=N imine stretch is at 1623 cm^{-1}.

Me_3TFB-Me_4PA

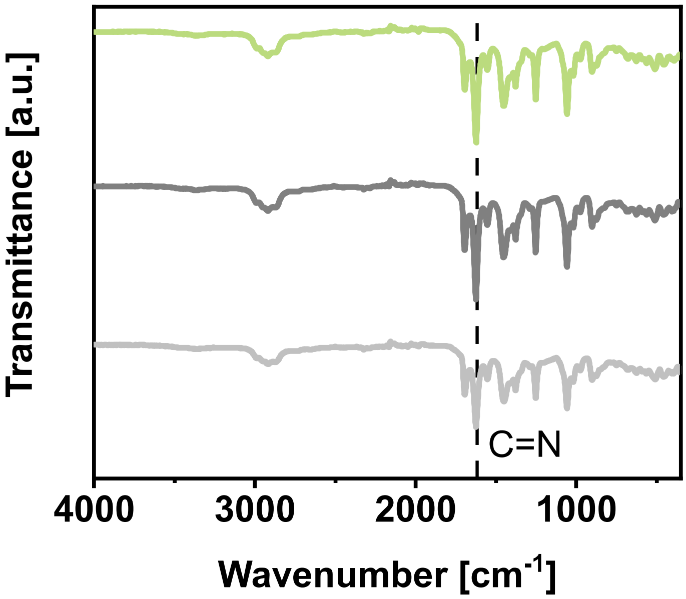

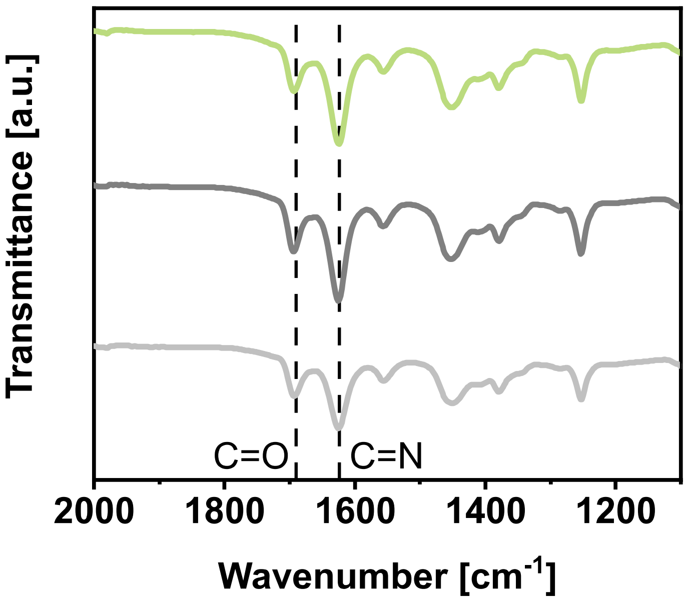

Figure S2. FT-IR spectra of triplicate Me_3TFB-Me_4PA COFs to show the repeatability of the synthesis. The C=N imine stretch is at 1625 cm^{-1}.

Me_3TFB-Me_2BD

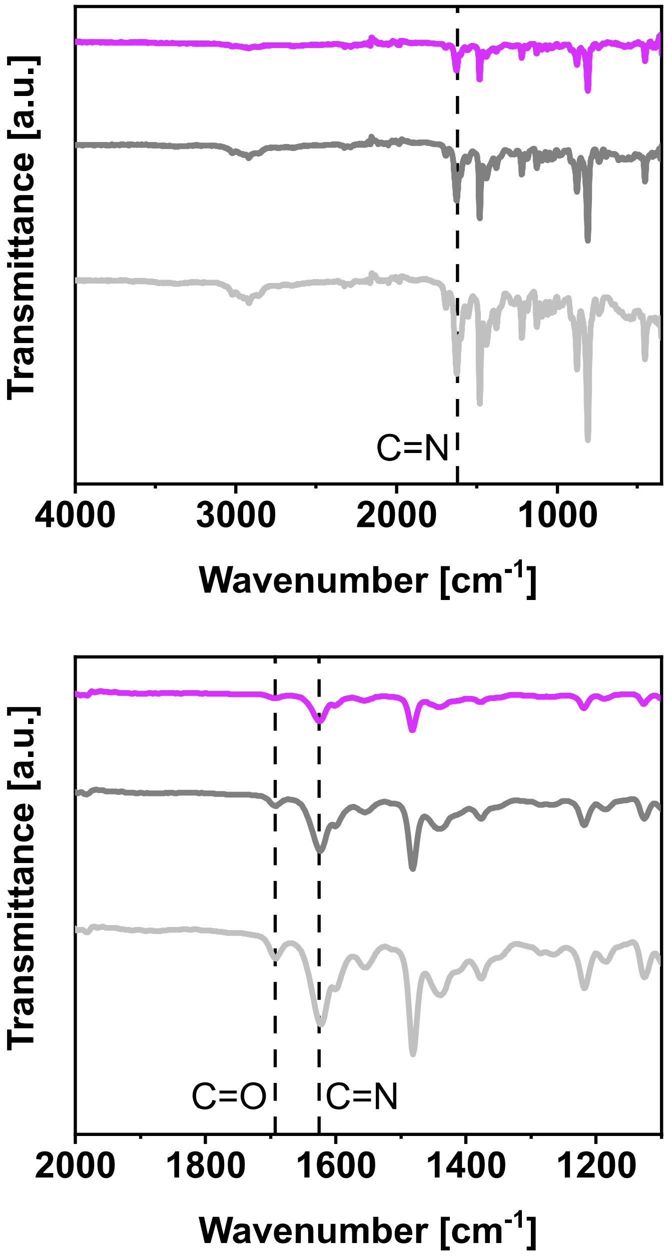

Figure S3. FT-IR spectra of triplicate Me_3TFB-Me_2BD COFs to show the repeatability of the synthesis. The C=N imine stretch is at 1624 cm^{-1}.

Me_3TFB-Me_4BD

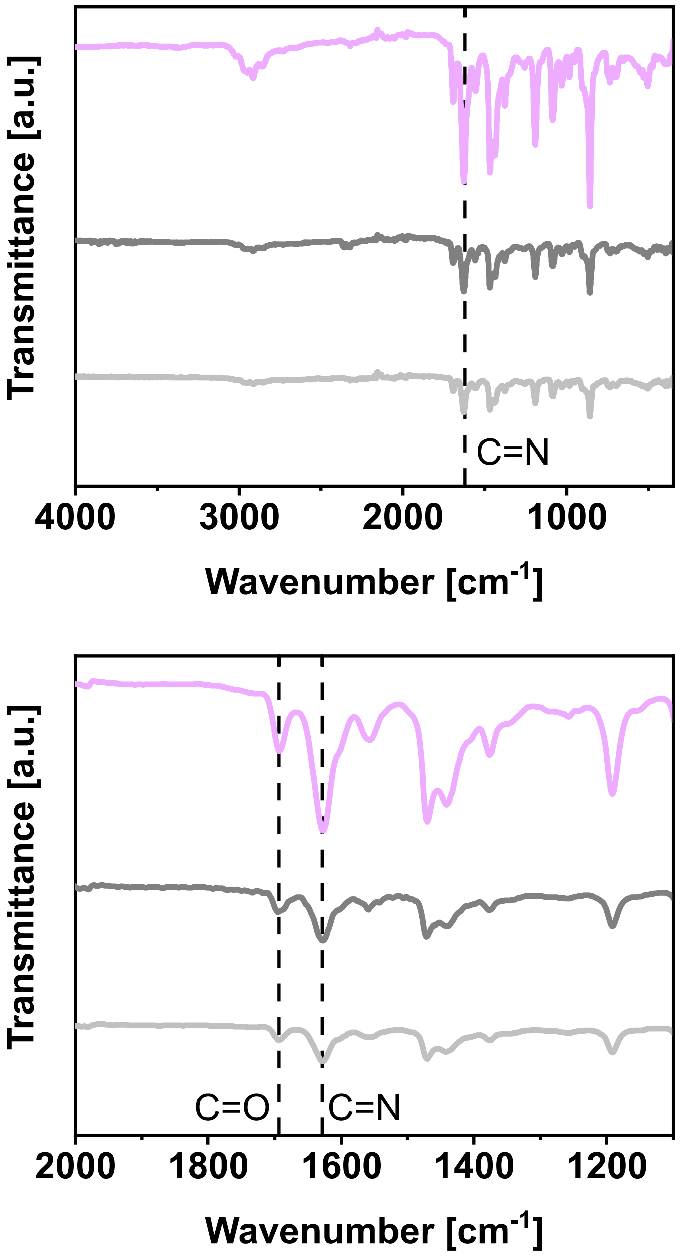

Figure S4. FT-IR spectra of triplicate Me_3TFB-Me_4BD COFs to show the repeatability of the synthesis. The C=N imine stretch is at 1627 cm^{-1}.

TFB-Me$_2$PA

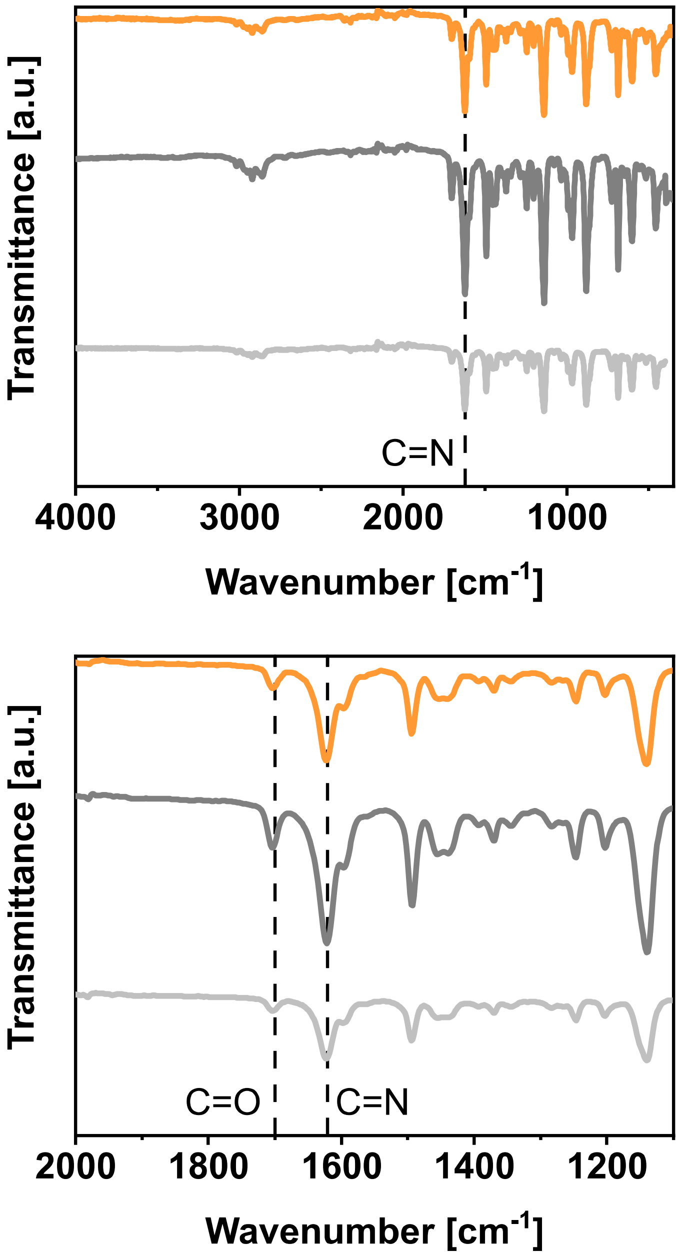

Figure S5. FT-IR spectra of triplicate TFB-Me$_2$PA COFs to show the repeatability of the synthesis. The C=N imine stretch is at 1623 cm^{-1}.

TFB-Me$_4$PA

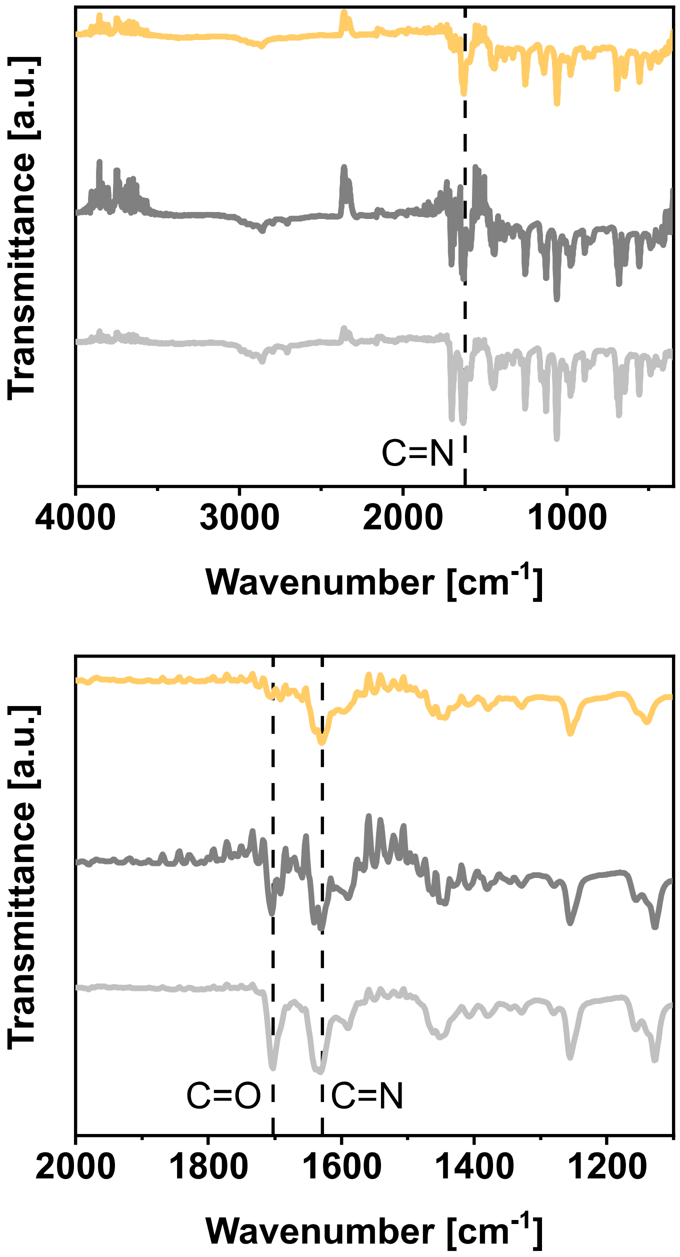

Figure S6. FT-IR spectra of triplicate TFB-Me$_4$PA COFs to show the repeatability of the synthesis. The C=N imine stretch is at 1632 cm^{-1}.

TFB-Me_2BD

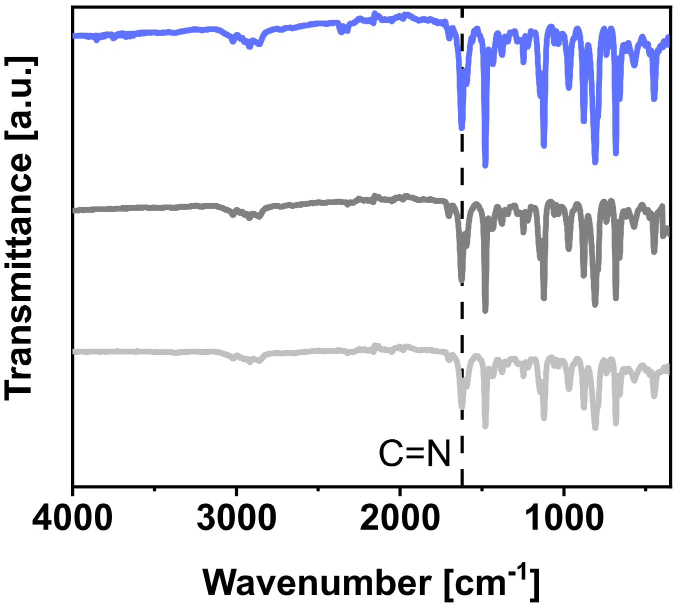

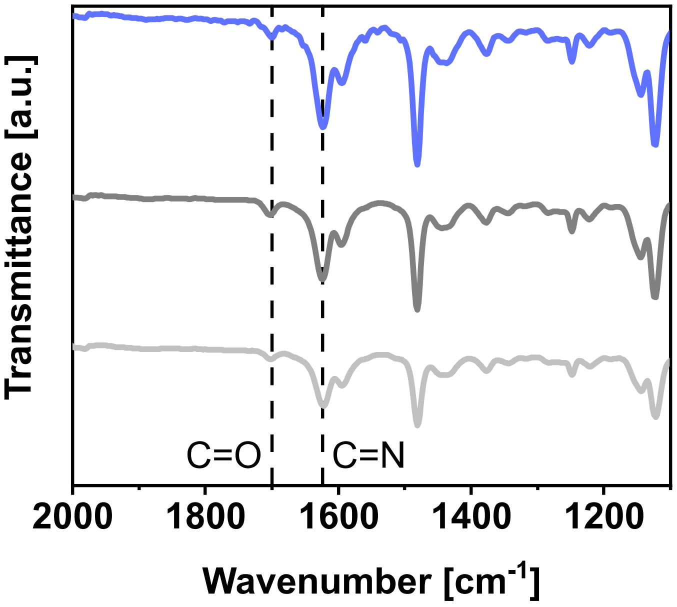

Figure S7. FT-IR spectra of triplicate TFB-Me_2BD COFs to show the repeatability of the synthesis. The C=N imine stretch is at 1623 cm^{-1}.

TFB-Me_4BD

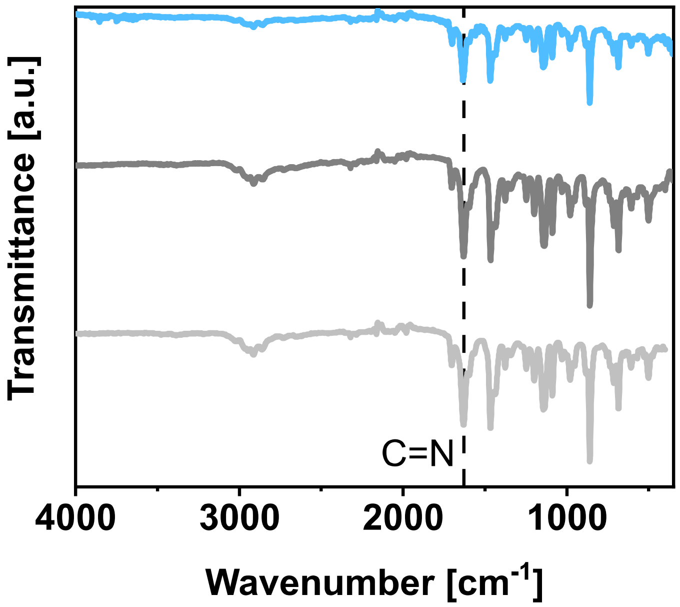

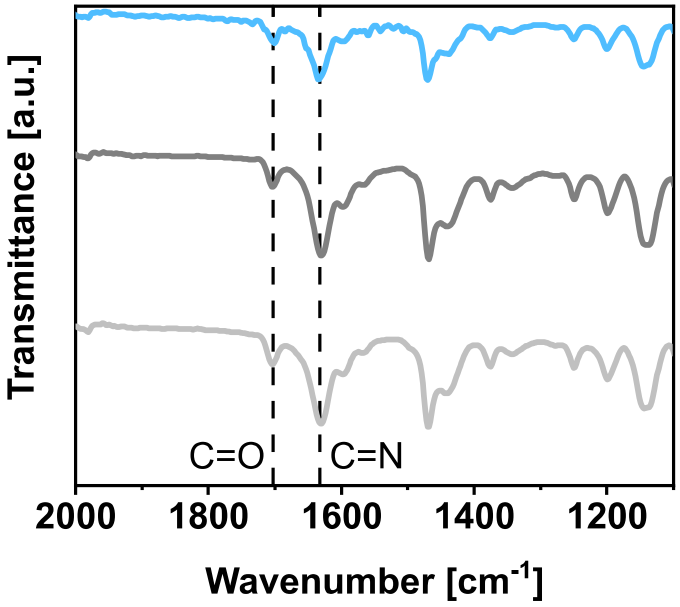

Figure S8. FT-IR spectra of triplicate TFB-Me_4BD COFs to show the repeatability of the synthesis. The C=N imine stretch is at 1631 cm^{-1}.

C. Powder X-Ray Diffraction Analysis

All FT-IR spectra indicate that the synthesis was repeatable which is also confirmed by nitrogen sorption measurements. Therefore, only one representative PXRD and ssNMR spectra of the triplicates will be displayed.

PXRD pattern were simulated in VESTA after optimizing the structures. Two different structures (flat and non-planar) have been computed and the structure with the lowest energy has been chosen as comparison with the experimental PXRD. All COFs containing Me_3TFB are lowest in energy when the COF structure is non-planar. Those COFs have eclipsed crystal structures. The only exception is TFB-Me_4PA whose crystal structure has more overlap with the staggered model.

Me_3TFB-Me_2PA

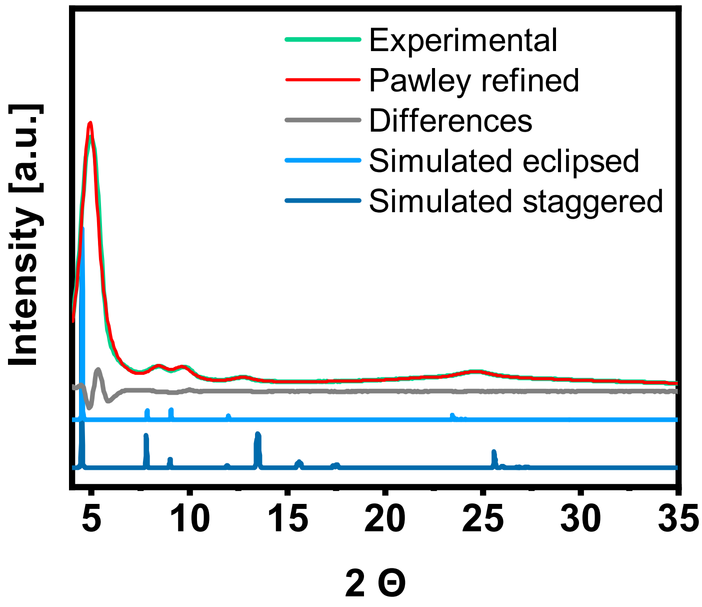

Figure S9. PXRD pattern of Me_3TFB-Me_2PA, including Pawley refinement, and modelled PXRD diffractions for eclipsed and staggered stacking.

Me_3TFB-Me_4PA

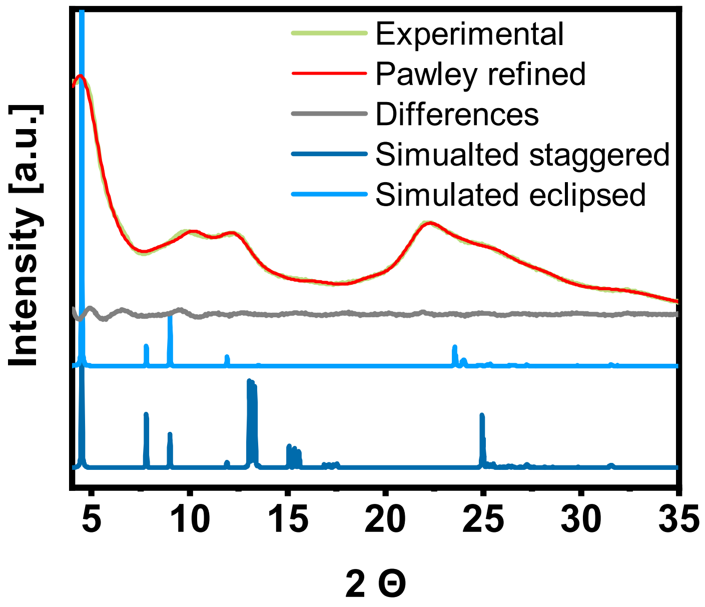

Figure S10. PXRD pattern of Me_3TFB-Me_4PA, including Pawley refinement, and modelled PXRD diffractions for eclipsed and staggered stacking.

Me_3TFB-Me_2BD

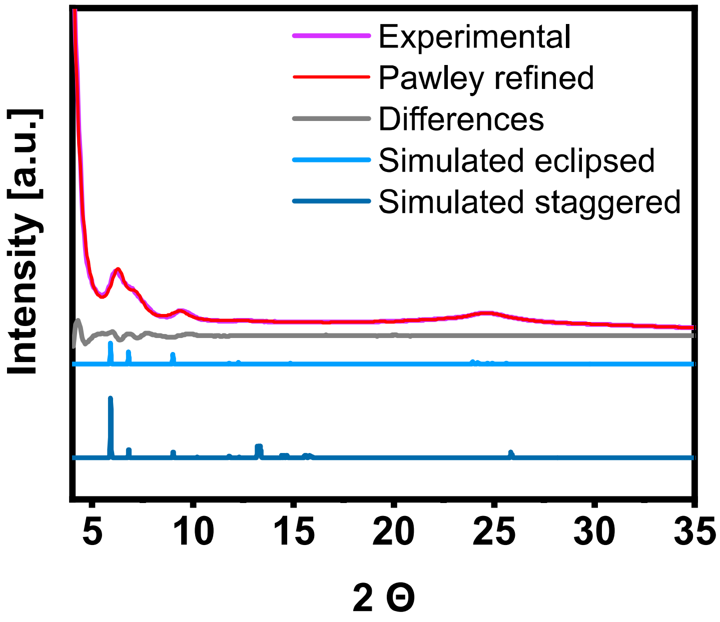

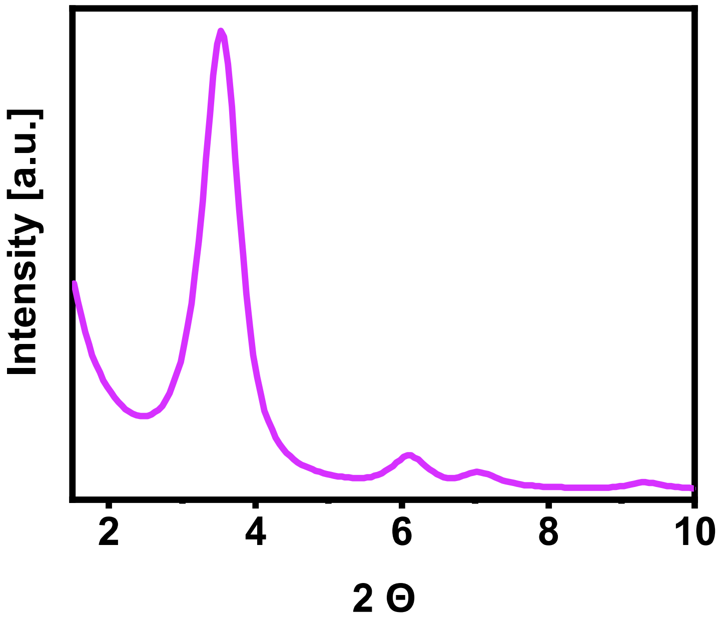

Figure S11. PXRD pattern of Me_3TFB-Me_2BD, including Pawley refinement, and modelled PXRD diffractions for eclipsed and staggered stacking (top). To resolve the peak at 3.5° better, a low angle PXRD pattern was recorded from 1-10° (bottom).

Me_3TFB-Me_4BD

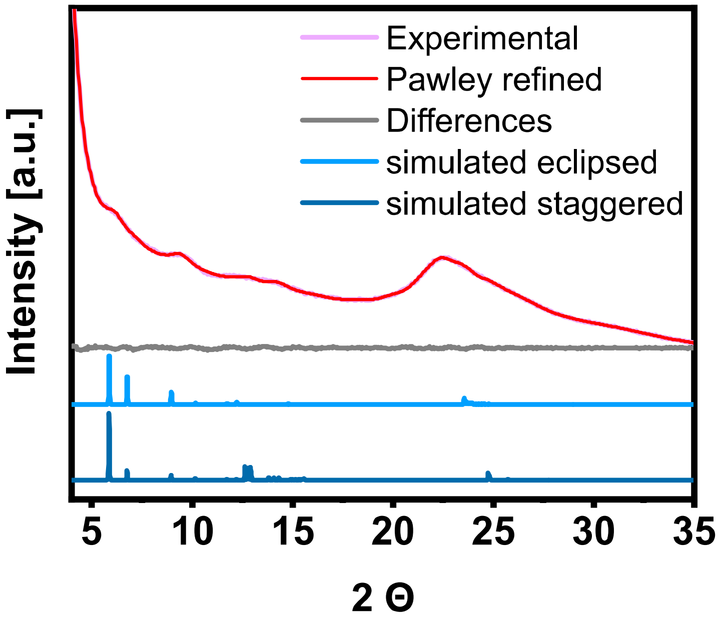

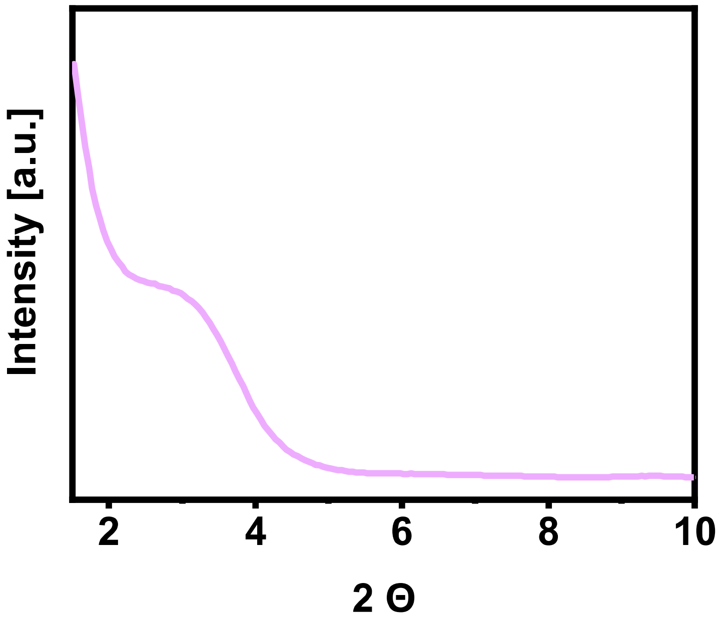

Figure S12. PXRD pattern of Me_3TFB-Me_4BD, including Pawley refinement, and modelled PXRD diffractions for eclipsed and staggered stacking. (top) To resolve the peak at 3.0° better, a low angle PXRD pattern was recorded from 1-10° (bottom).

TFB-Me_2PA

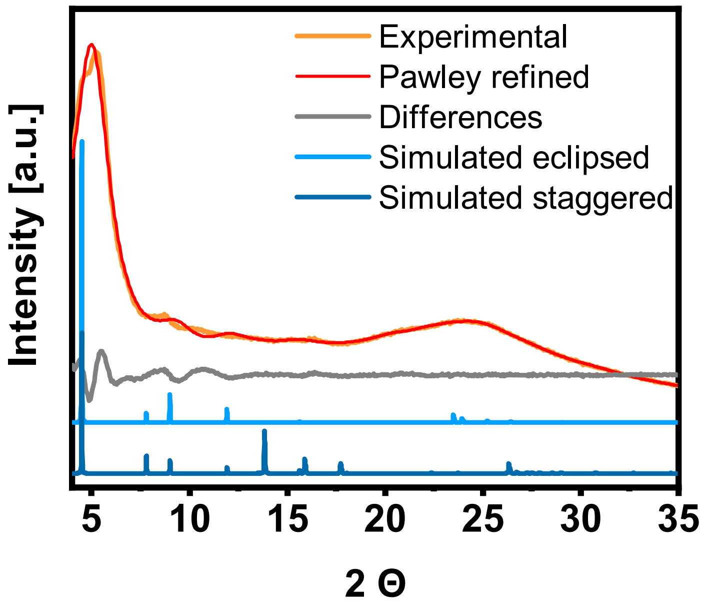

Figure S13. PXRD pattern of TFB-Me_2PA, including Pawley refinement, and modelled PXRD diffractions for eclipsed and staggered stacking.

TFB-Me_4PA

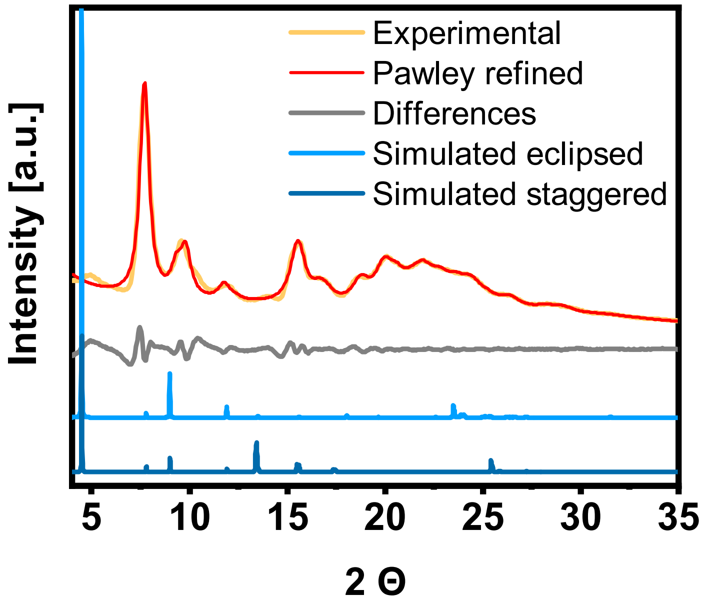

Figure S14. PXRD pattern of TFB-Me_4PA, including Pawley refinement, and modelled PXRD diffractions for eclipsed and staggered stacking.

TFB-Me$_2$BD

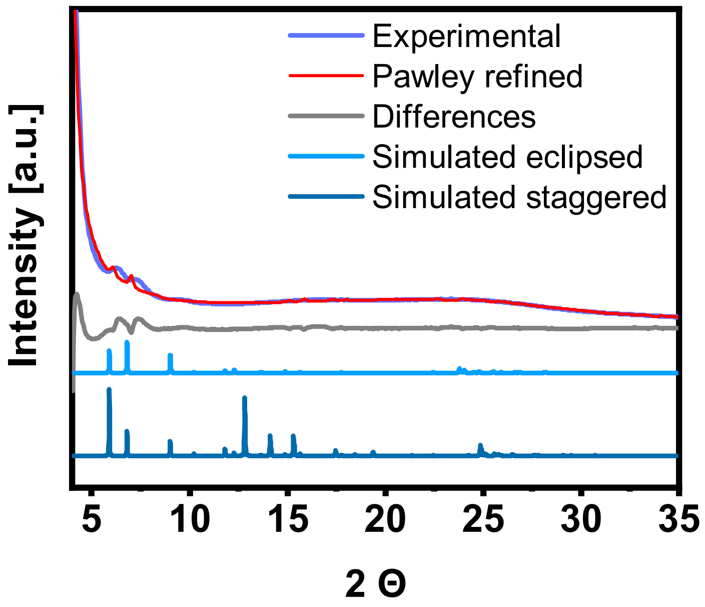

Figure S15. PXRD pattern of TFB-Me$_2$BD, including Pawley refinement, and modelled PXRD diffractions for eclipsed and staggered stacking.

TFB-Me$_4$BD

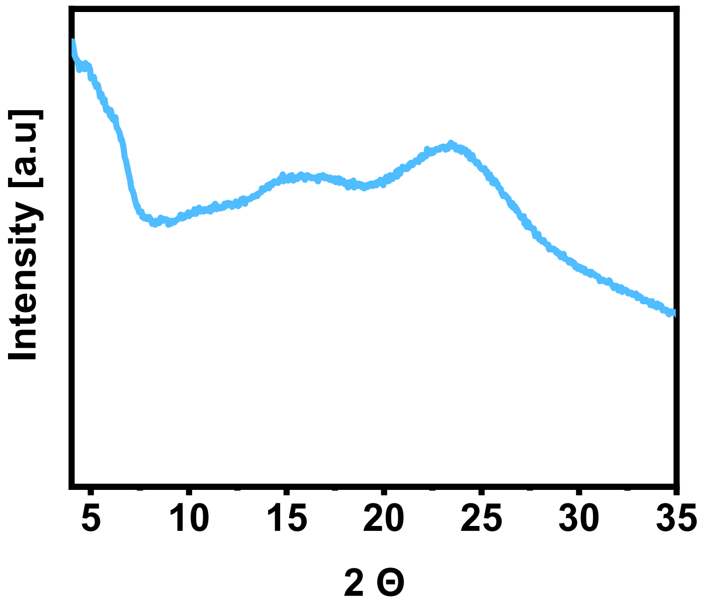

Figure S16. PXRD pattern of TFB-Me$_4$BD. The sample is amorphous.

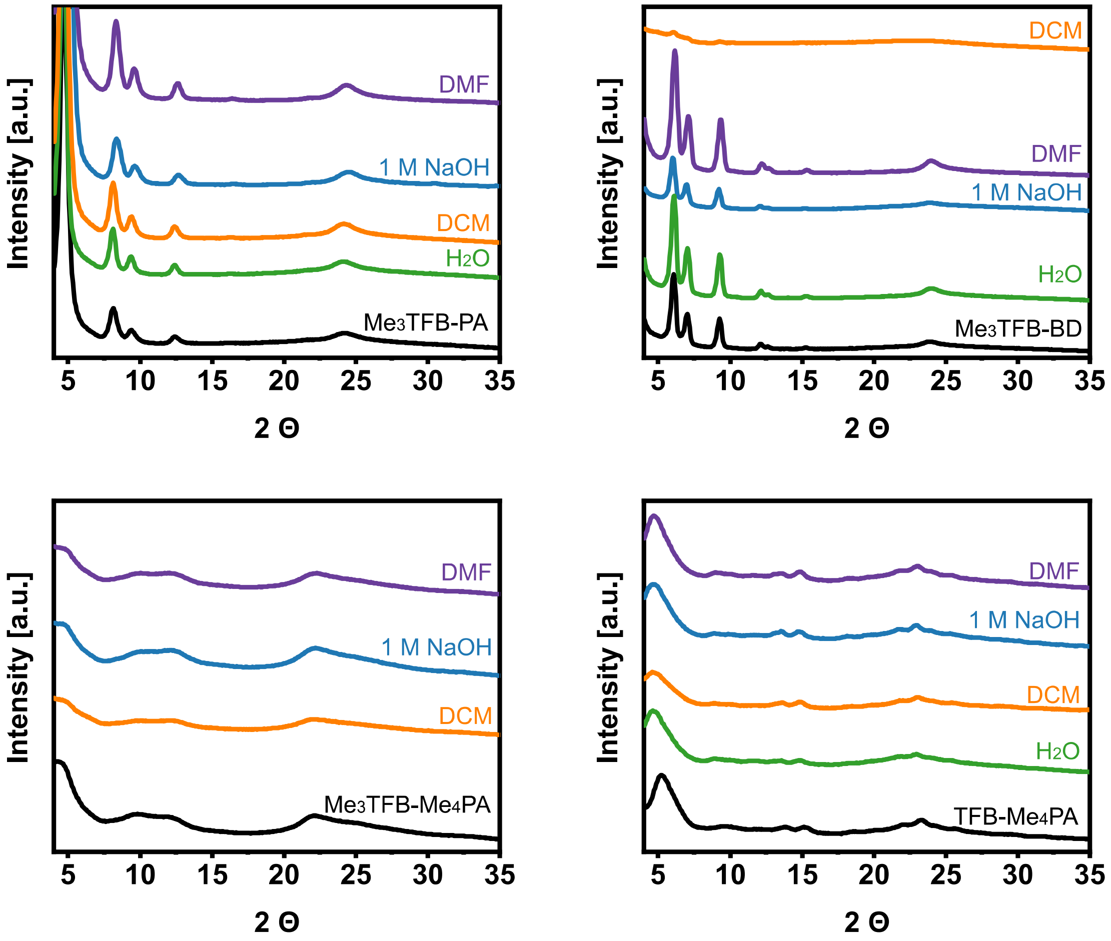

Figure S17. Different COFs immersed in several solvents for 5 days to check their chemical stability. The PXRD diffractograms were measured after re-isolation and drying at 120 °C overnight.

D. ^{13}C CPMAS NMR Spectra

This chapter is organized showing all ssNMR spectra for each COF.

If one is interested in ssNMR spectra at different contact times, the following spectra are relevant: SI Figure 18, 21, 22, 24, 27, 29, 30, 33, 35, 36, 39, 40. The ssNMR spectra for different contact times are displayed in SI Figure 19, 23, 25, 31, 37 and 41 and the spectra for the other COFs can be found in SI Figure 20, 26, 28, 32, 34 and 38.

Assignment TFB-PA / Me_3TFB-PA / TFB-Me_4PA

The ssNMR spectrum of TFB-PA has been assigned by derivatizing the COF in turning tertiary carbons into quaternary carbons and measuring the cross-polarization (CP) built-up for the signals. During the pulse-sequence, the polarization is transferred from a proton to a nearby carbon atom which is then detected. The contact time is the experimental parameter, which sets the length of the contact between proton and carbon spins. Compared to tertiary carbons, quaternary carbons – which do not have a proton in their proximity – need longer contact times until the signals are build-up. Therefore, tertiary signals are already visible in the spectrum at shorter contact times, whereas quaternary carbon signals become visible with increasing contact times.

In comparing the spectra to each other, the change in CP built-up indicates which carbon atom gives the respective signal. Based on the chemical shift, the signal around 160 ppm could be assigned to the imine carbon, leaving four other signals left which needs to be assigned. The methyl group carbon signal is around 15 ppm in the respective derivatives. The CP built-up curves indicate two tertiary and two quaternary signals as expected. The signal of the quaternary carbon around 145 ppm is shifted more downfield compared to the quaternary carbon around 135 ppm. Based on the chemical shift, the signal around 145 ppm can be assigned to the carbon atom next to the nitrogen atom (C-N) and the left quaternary carbon signal belongs to the quaternary carbon in the aldehyde node. Both Me3TFB-PA and TFB-Me4PA transform one of the tertiary signals into a quaternary carbon leading to a slower CP built-up which is confirmed by our measurements. Therefore, the signal around 131 ppm can be assigned to the other carbon atom of the aldehyde node and the signal around 120 ppm belongs to the other carbon of the amine linker. The grey signal in **Figure S18**B2 comes from fitting the shoulder of the signal at 120 ppm. This shoulder might belong to unreacted groups within the polymer.

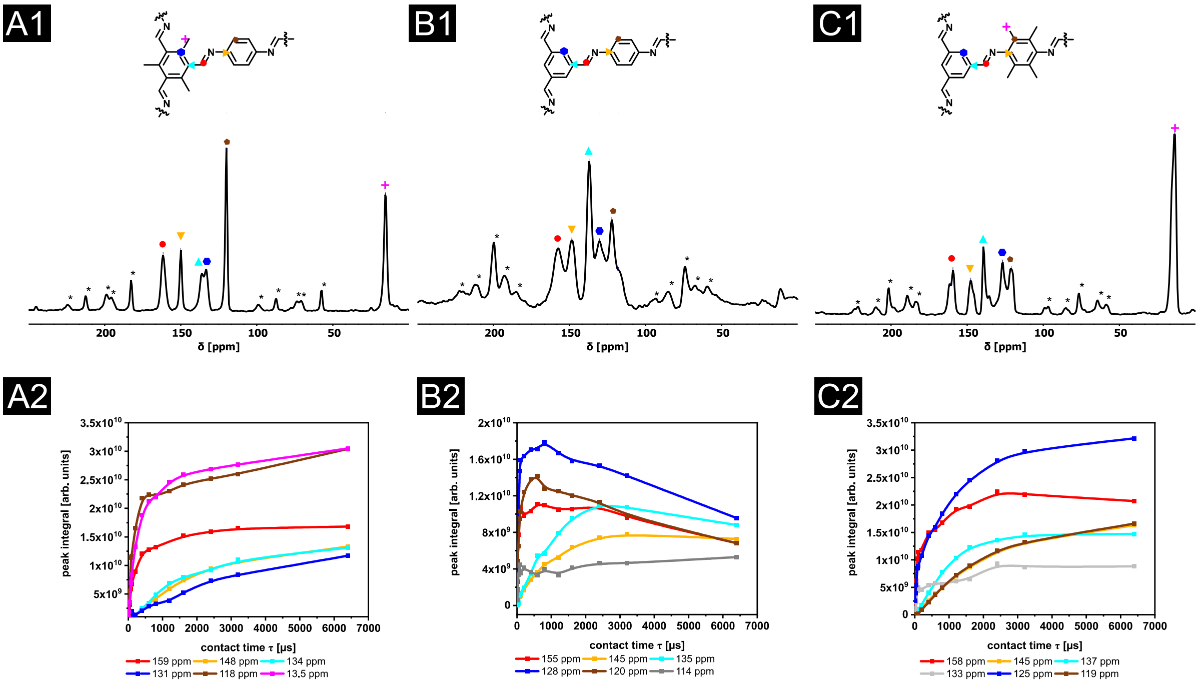

Figure S18. ^{13}C CPMAS solid state NMR spectra of respective COF (black): (A) Me_3TFB-PA, (B) TFB-PA and (C) TFB-Me_4PA in the top (A1-C1) and CP build-up curves in the bottom (A2-C2). Spinning side bands are denoted with an asterisk.

TFB-PA

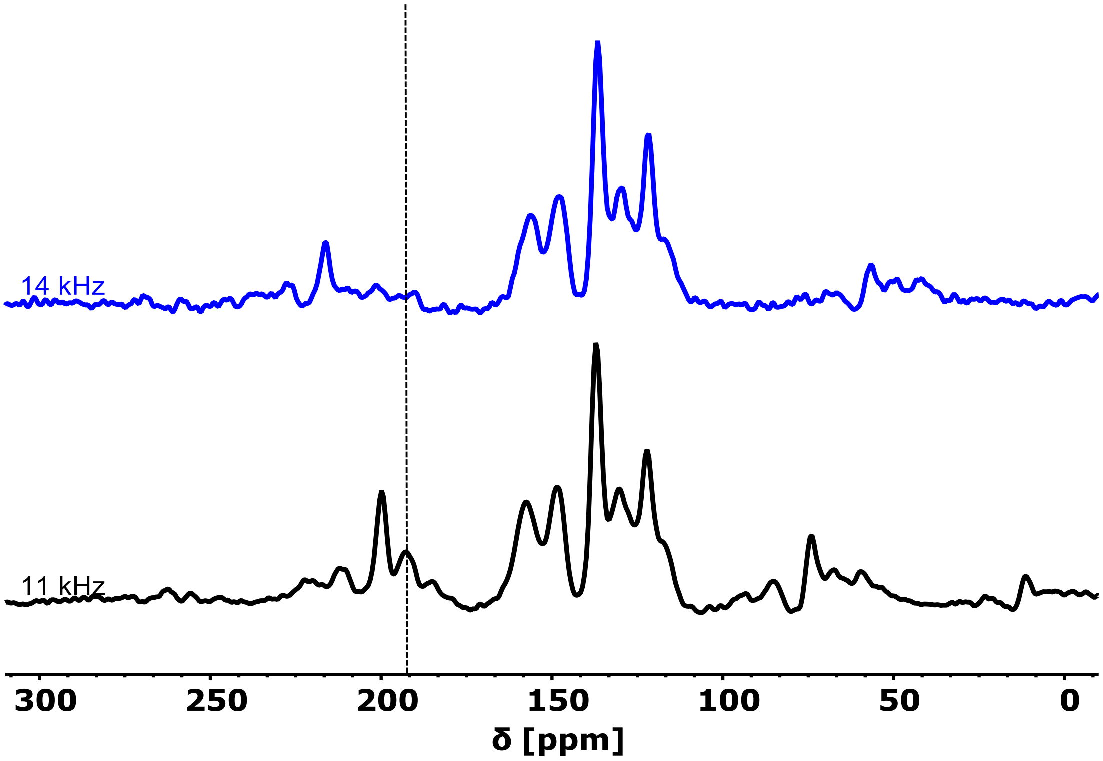

Figure S19. ^{13}C CPMAS solid state NMR spectra at 14 kHz and 11 kHz to determine spinning side bands. The vertical, dashed line indicates the chemical shift of residual aldehyde groups.

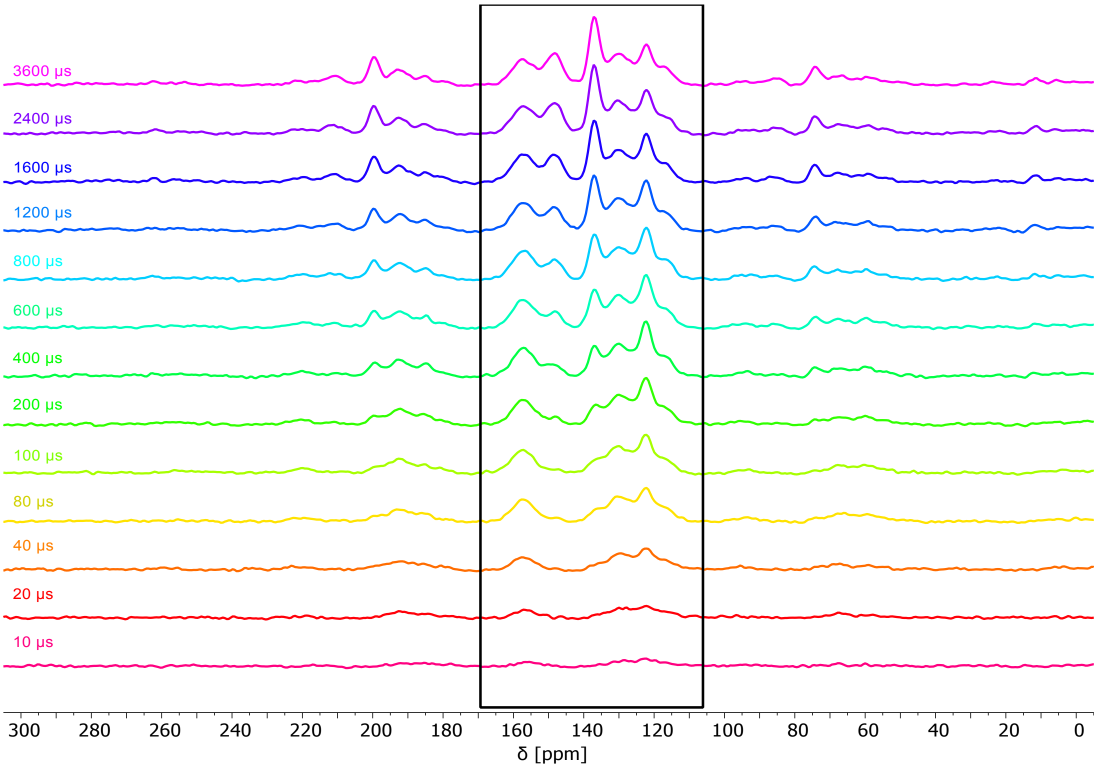

Figure S20. ^{13}C CPMAS solid state NMR spectra at 11 kHz and different contact times.

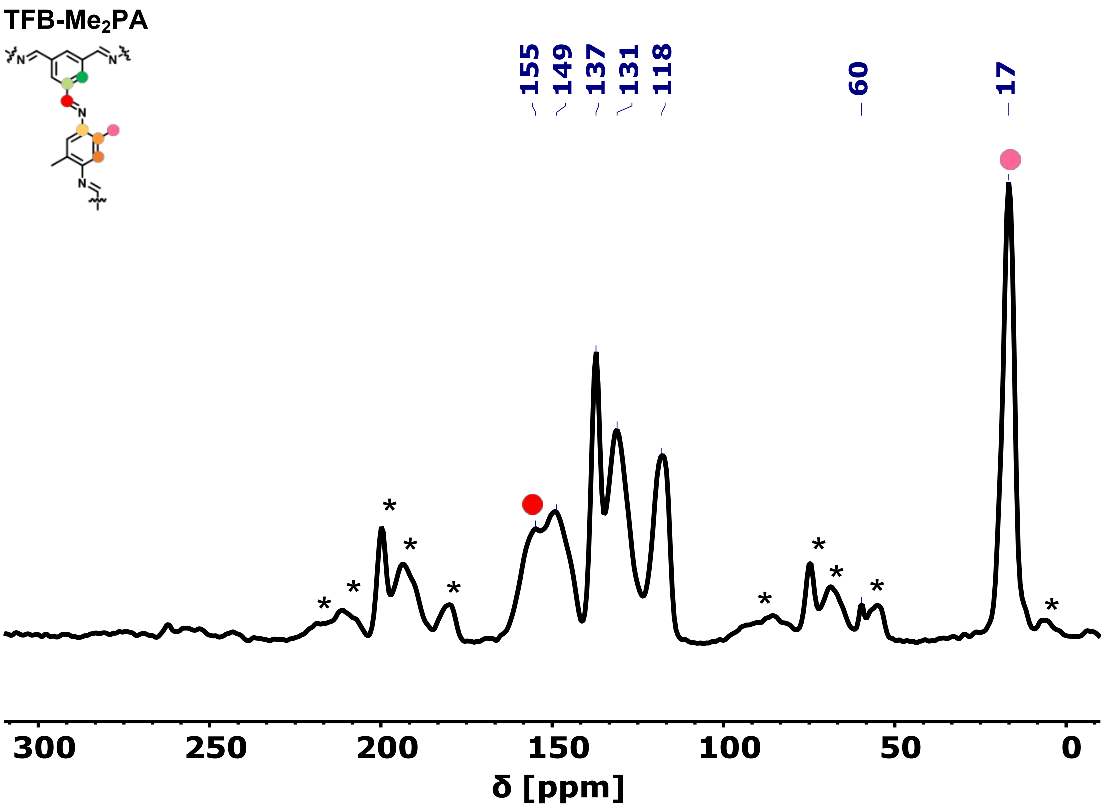

Figure S21. ^{13}C CPMAS solid state NMR spectrum at 11 kHz. Spinning side bands, here indicated with an asterisk were determined by comparing different MAS frequencies. The signal at 155 ppm can be assigned to the carbon of an imine-bound nitrogen, indicating the formation of imine bonds.

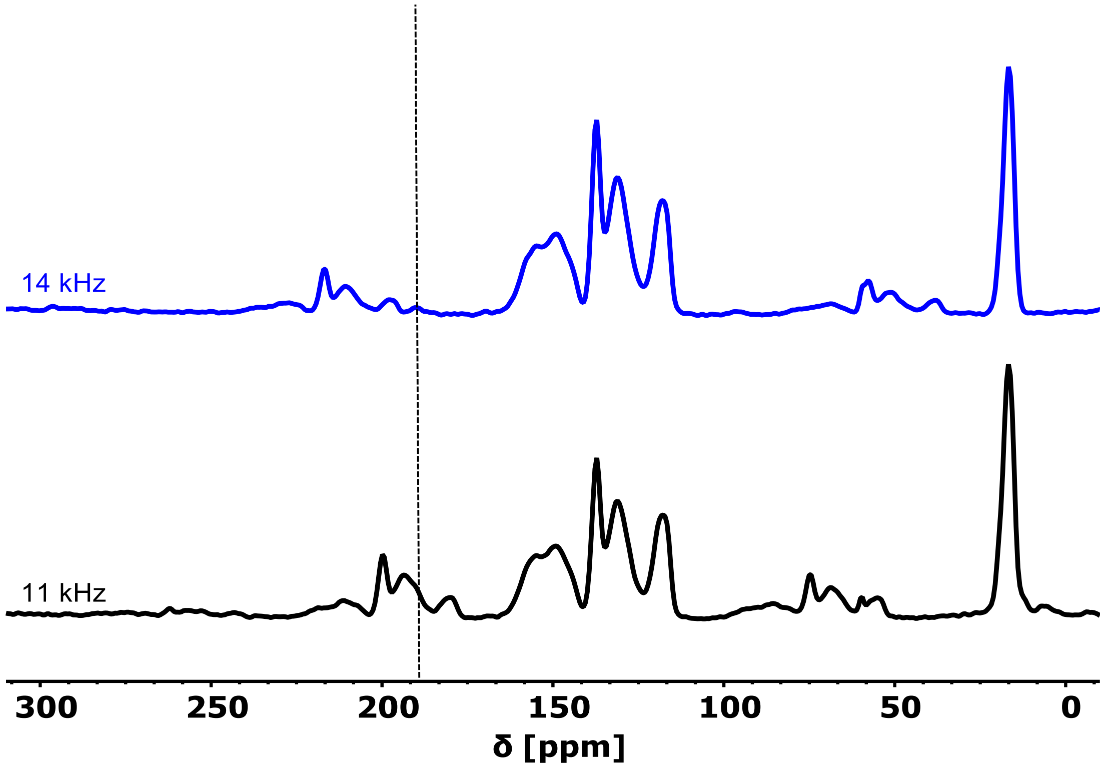

Figure S22. ^{13}C CPMAS solid state NMR spectra at 14 kHz and 11 kHz to determine spinning side bands. Vertical line indicates the chemical shift of residual aldehyde groups.

TFB-Me$_4$PA

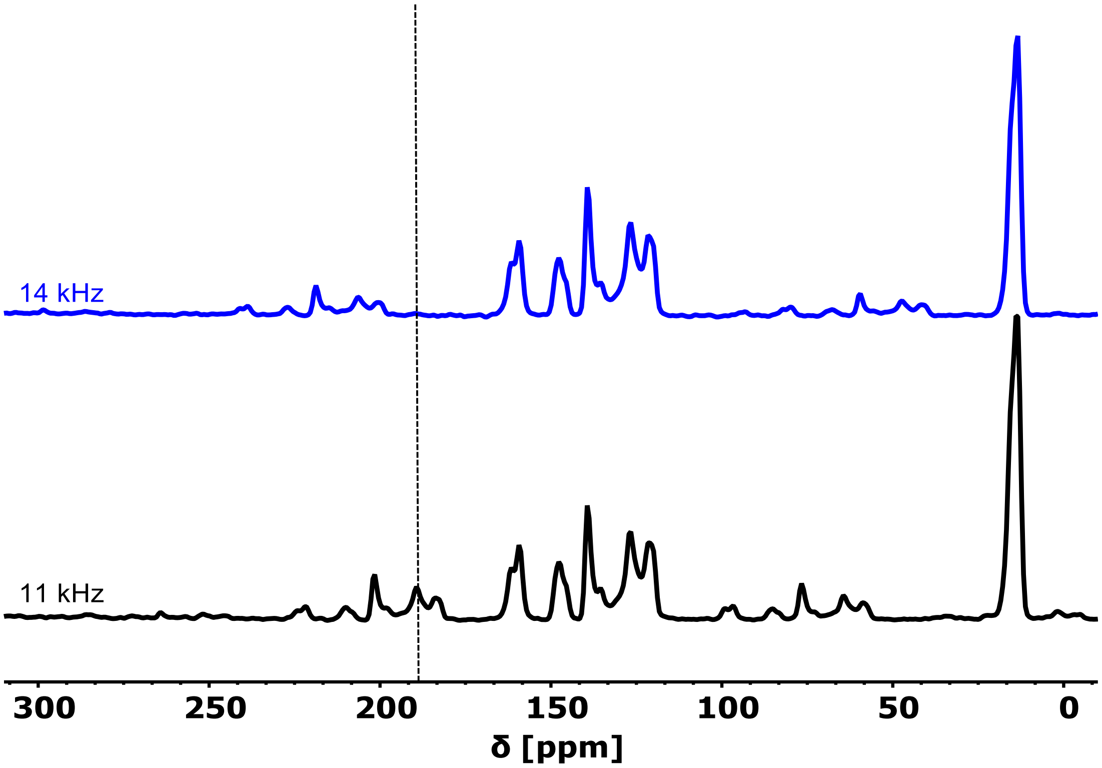

Figure S23. ^{13}C CPMAS solid state NMR spectra at 14 kHz and 11 kHz to determine spinning side bands. The vertical, dashed line indicates the chemical shift of residual aldehyde groups.

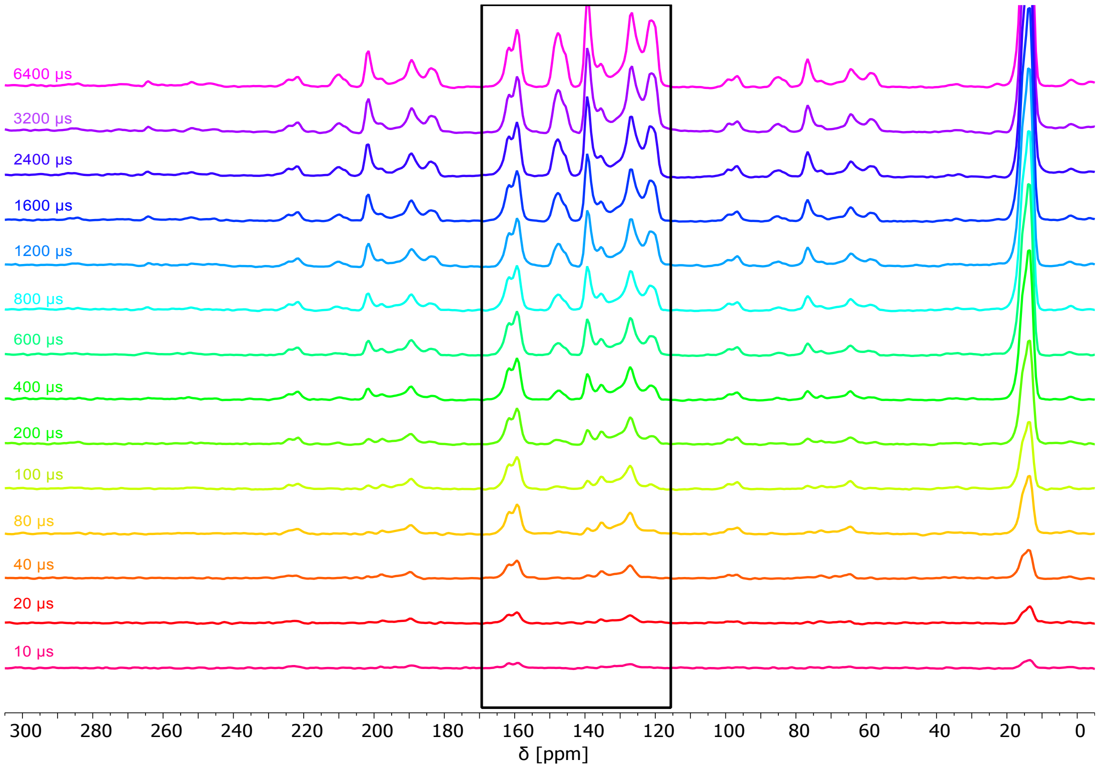

Figure S24. ^{13}C CPMAS solid state NMR spectra at 11 kHz and different contact times.

TFB-BD

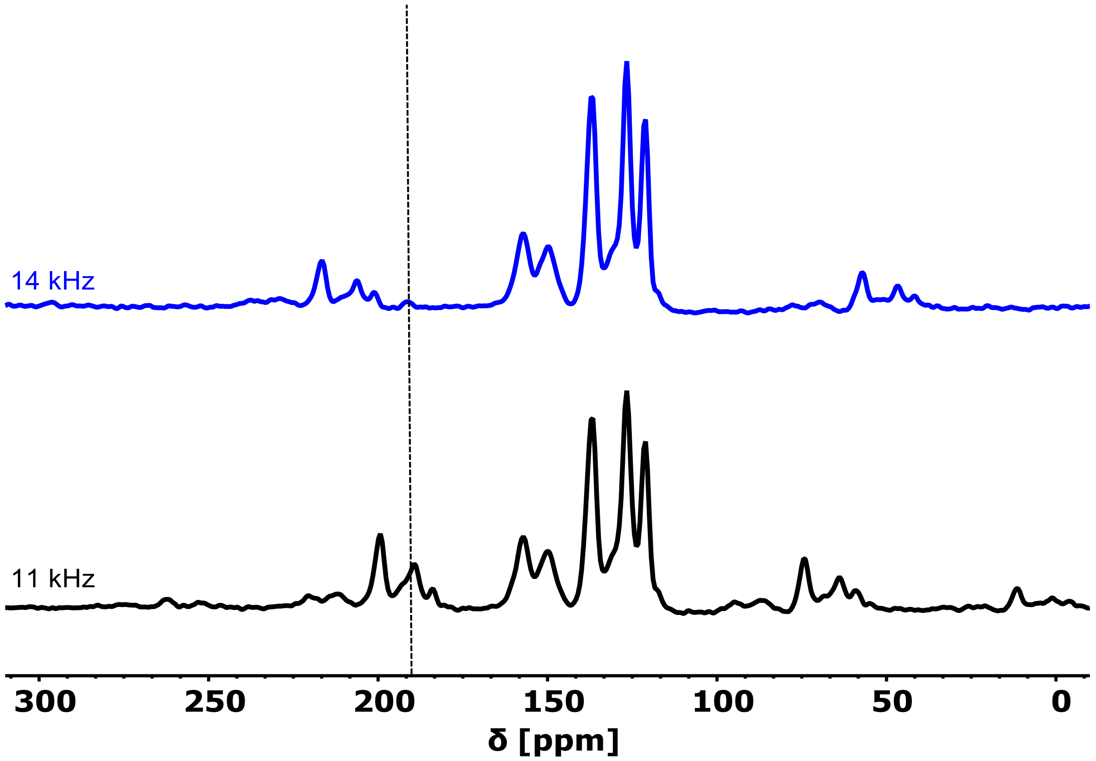

Figure S25. ^{13}C CPMAS solid state NMR spectra at 14 kHz and 11 kHz to determine spinning side bands. The vertical, dashed line indicates the chemical shift of residual aldehyde groups.

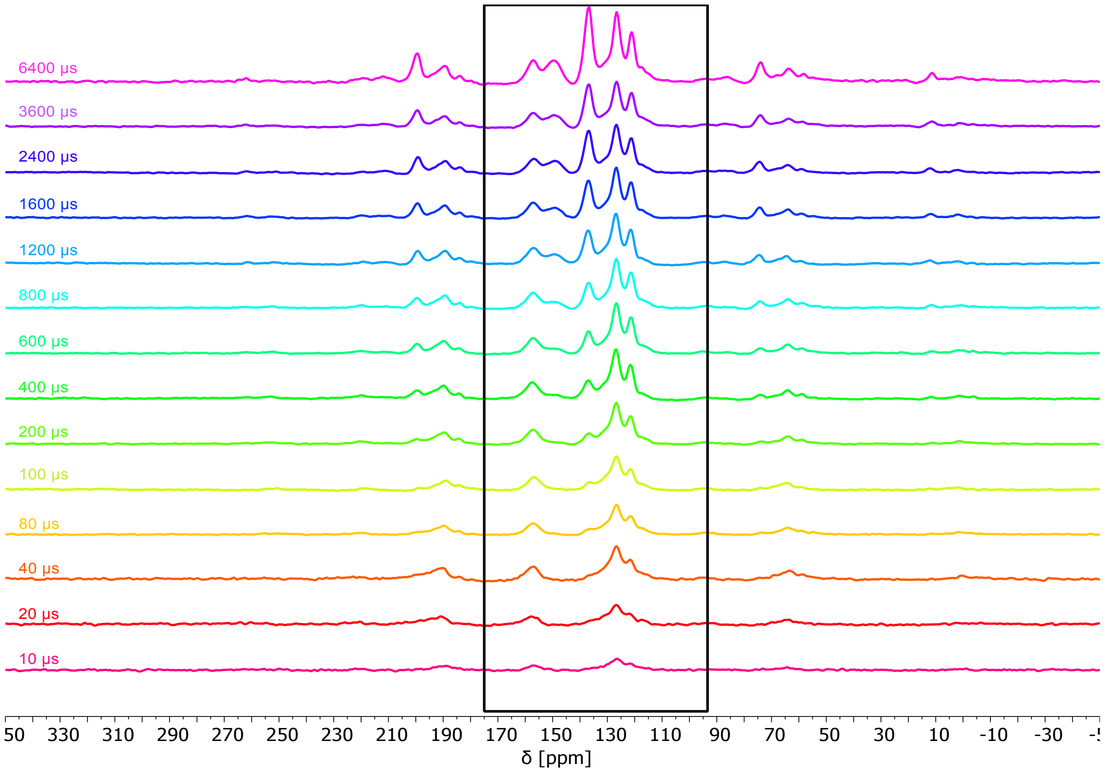

Figure S26. ^{13}C CPMAS solid state NMR spectra at 11 kHz and different contact times.

TFB-Me_2BD

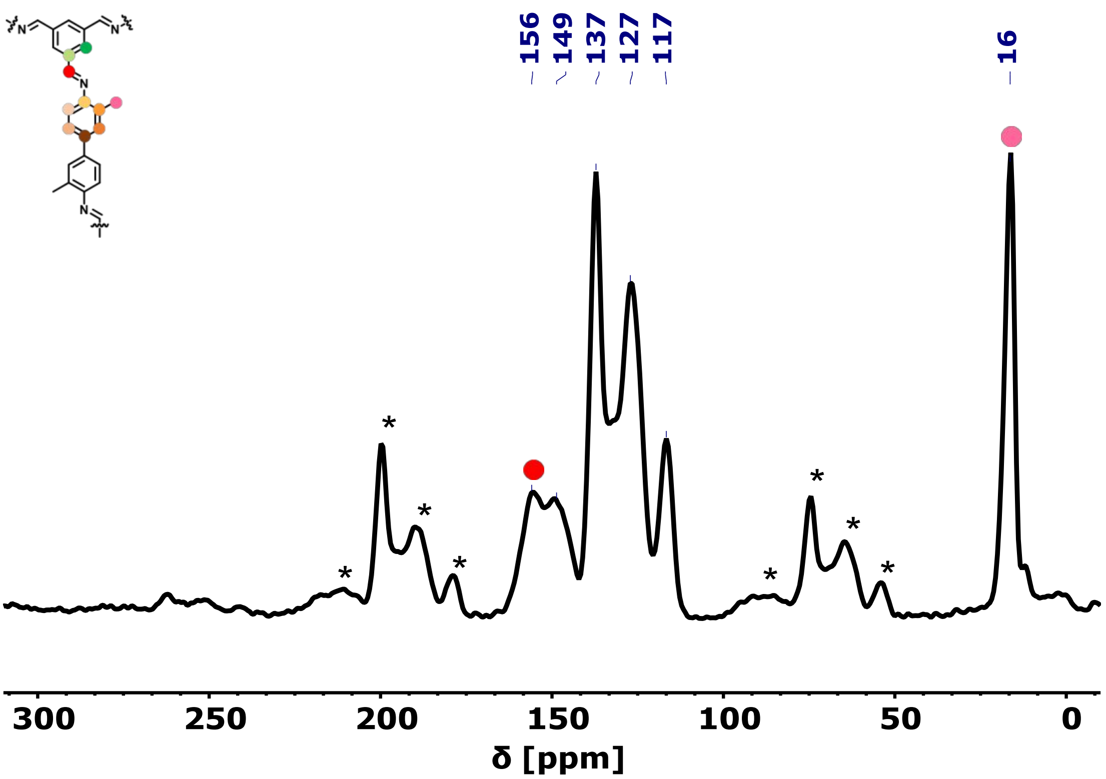

Figure S27. ^{13}C CPMAS solid state NMR spectrum at 11 kHz. Spinning side bands, here indicated with an asterisk were determined by comparing different MAS frequencies. The signal at 156 ppm can be assigned to the carbon of an imine-bound nitrogen, indicating the formation of imine bonds.

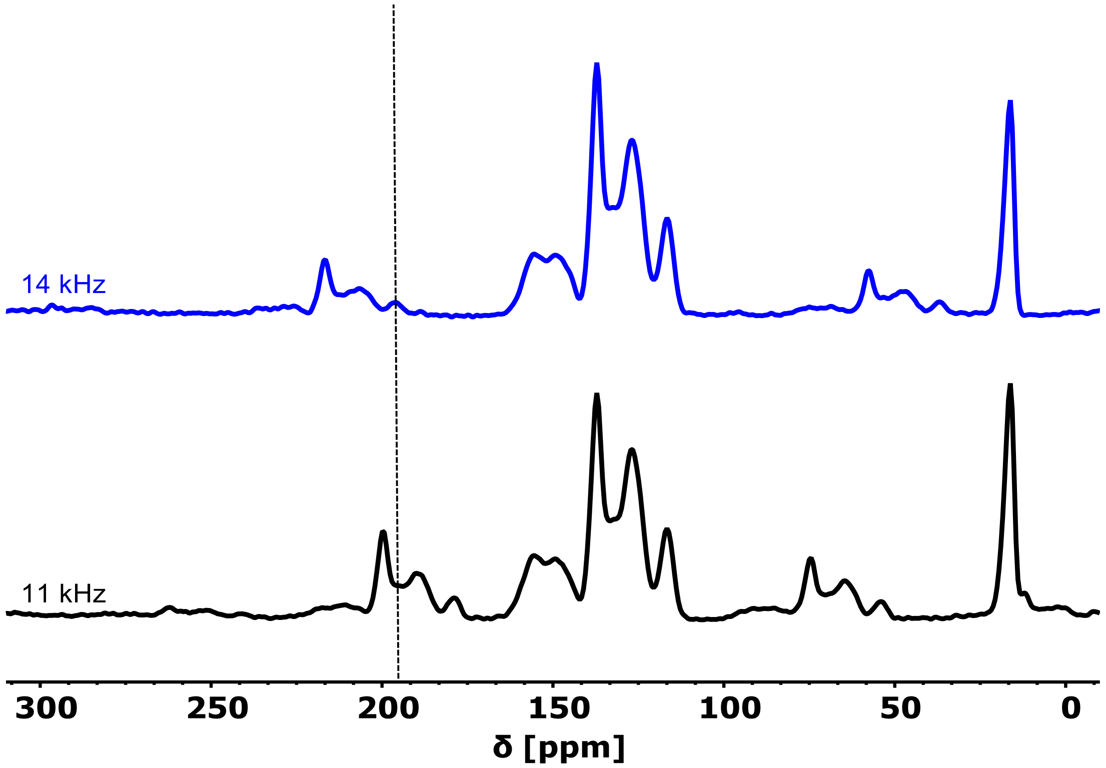

Figure S28. ^{13}C CPMAS solid state NMR spectra at 14 kHz and 11 kHz to determine spinning side bands. The vertical, dashed line indicates the chemical shift of residual aldehyde groups.

TFB-Me$_4$BD

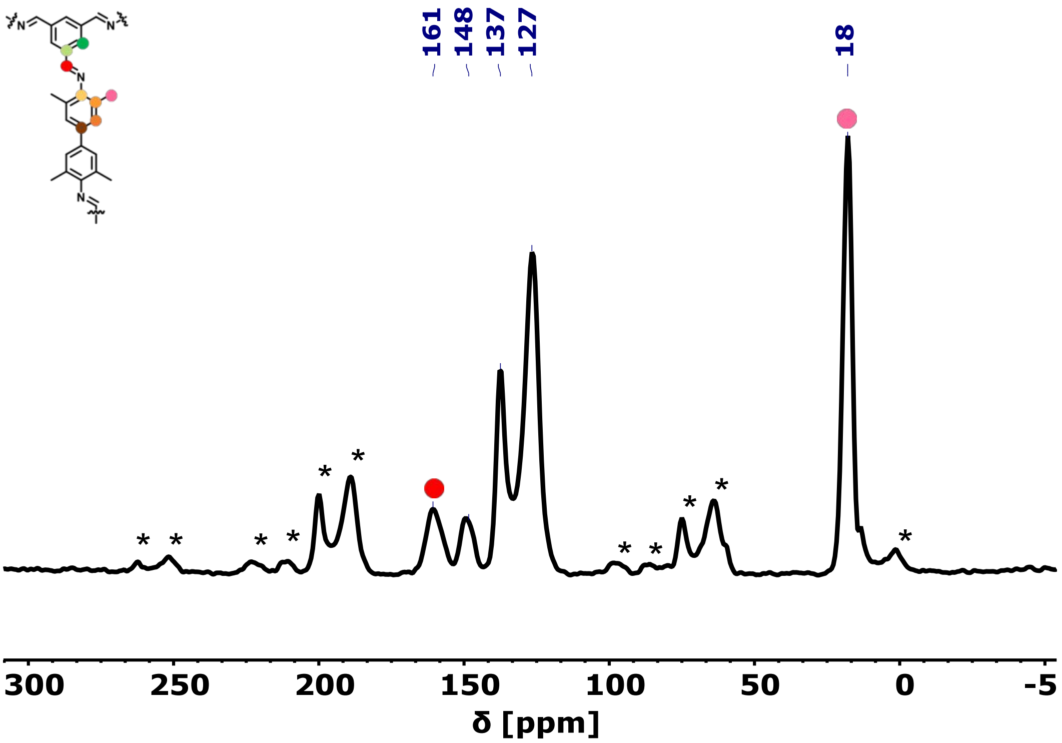

Figure S29. ^{13}C CPMAS solid state NMR spectrum at 11 kHz. Spinning side bands, here indicated with an asterisk were determined by comparing different MAS frequencies. The signal at 161 ppm can be assigned to the carbon of an imine-bound nitrogen, indicating the formation of imine bonds.

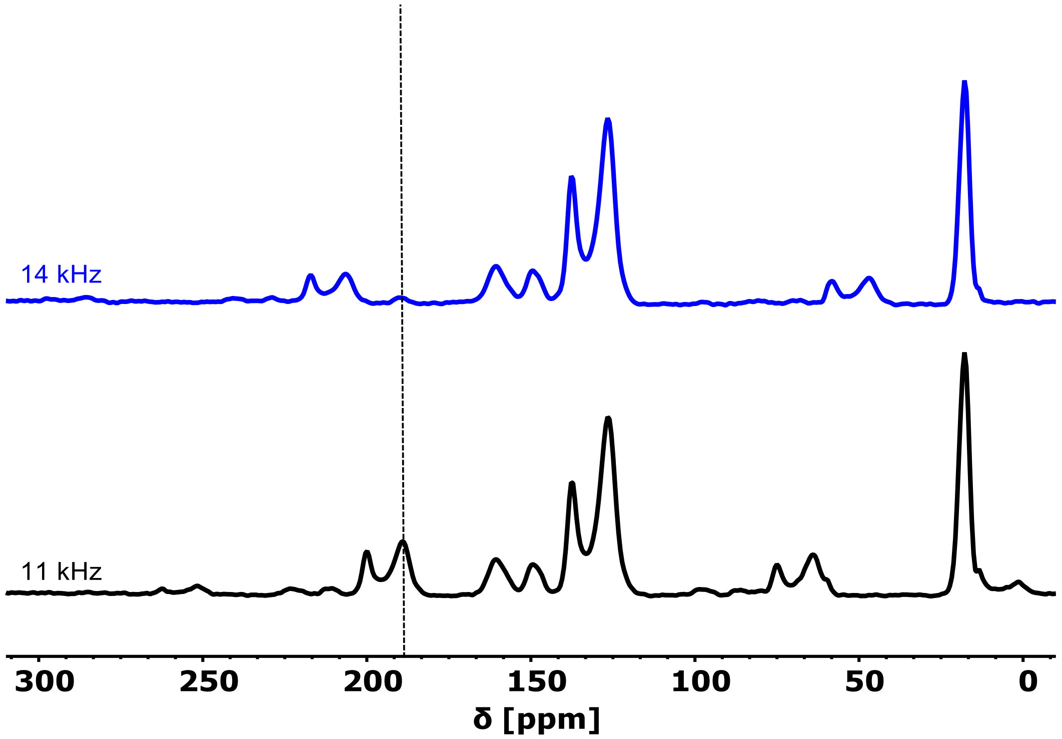

Figure S30. ^{13}C CPMAS solid state NMR spectra at 14 kHz and 11 kHz to determine spinning side bands. The vertical, dashed line indicates the chemical shift of residual aldehyde groups.

Me_3TFB-PA

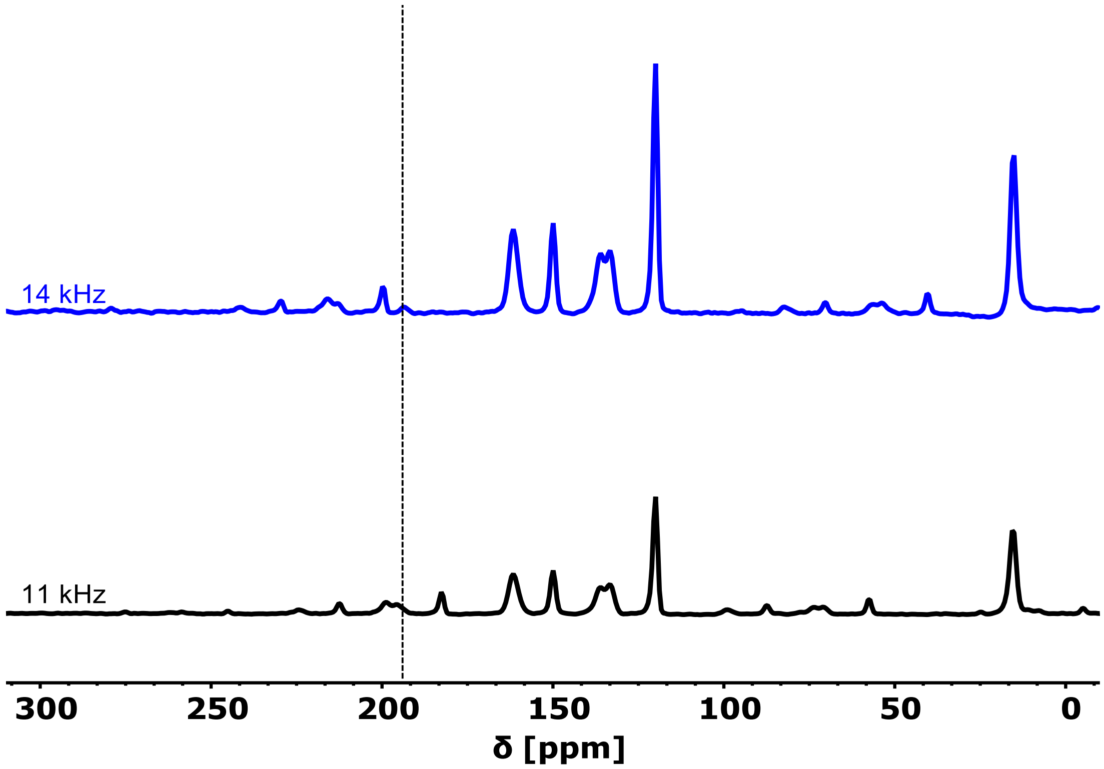

Figure S31. ^{13}C CPMAS solid state NMR spectra at 14 kHz and 11 kHz to determine spinning side bands. The vertical, dashed line indicates the chemical shift of residual aldehyde groups.

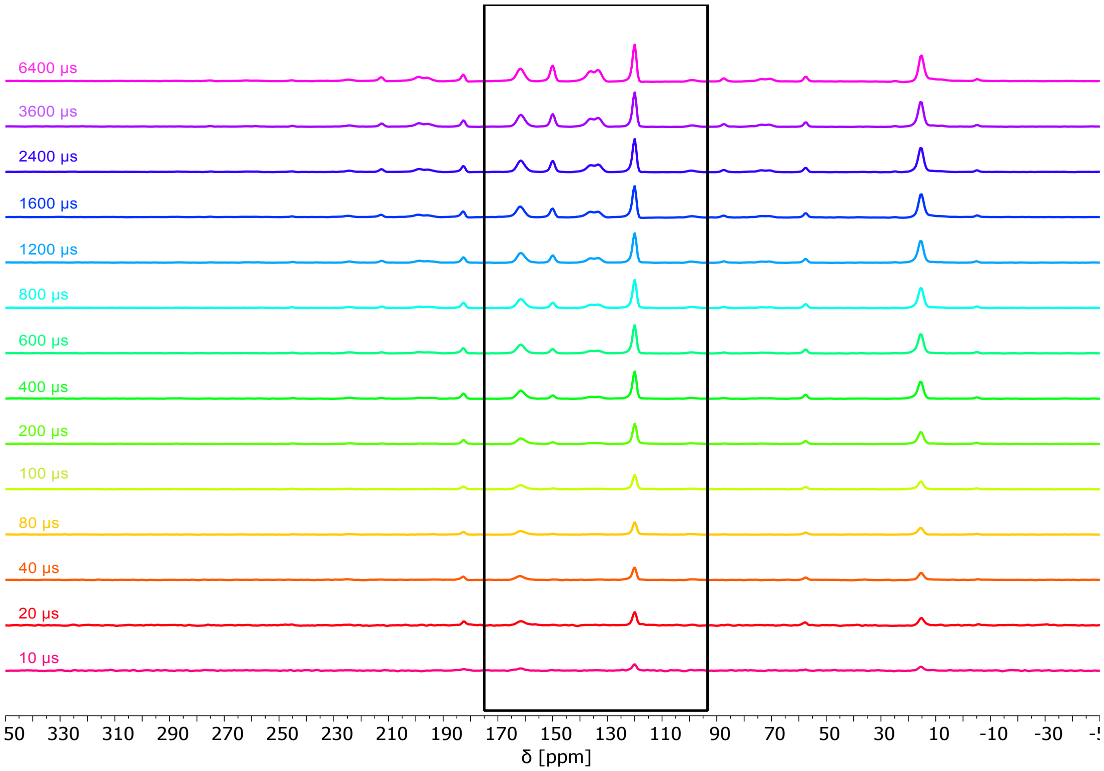

Figure S32. ^{13}C CPMAS solid state NMR spectra at 11 kHz and different contact times.

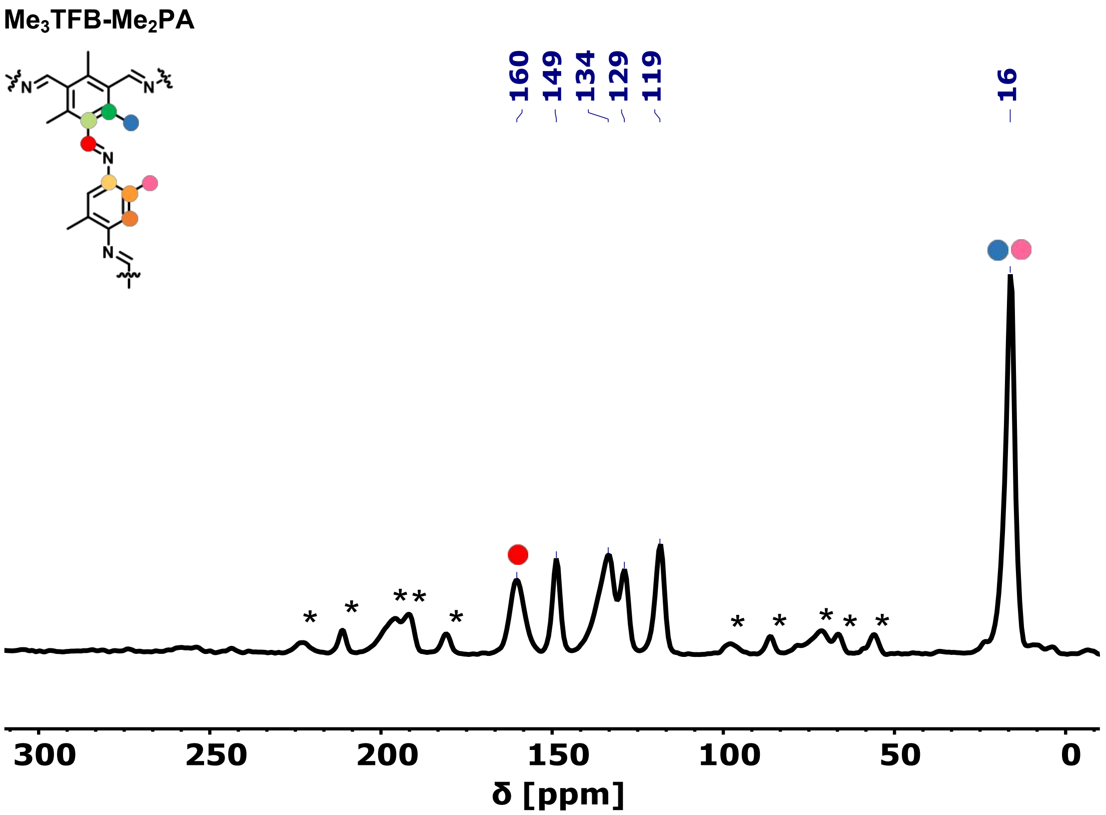

Figure S33. ^{13}C CPMAS solid state NMR spectrum at 11 kHz. Spinning side bands, here indicated with an asterisk were determined by comparing different MAS frequencies. The signal at 160 ppm can be assigned to the carbon of an imine-bound nitrogen, indicating the formation of imine bonds.

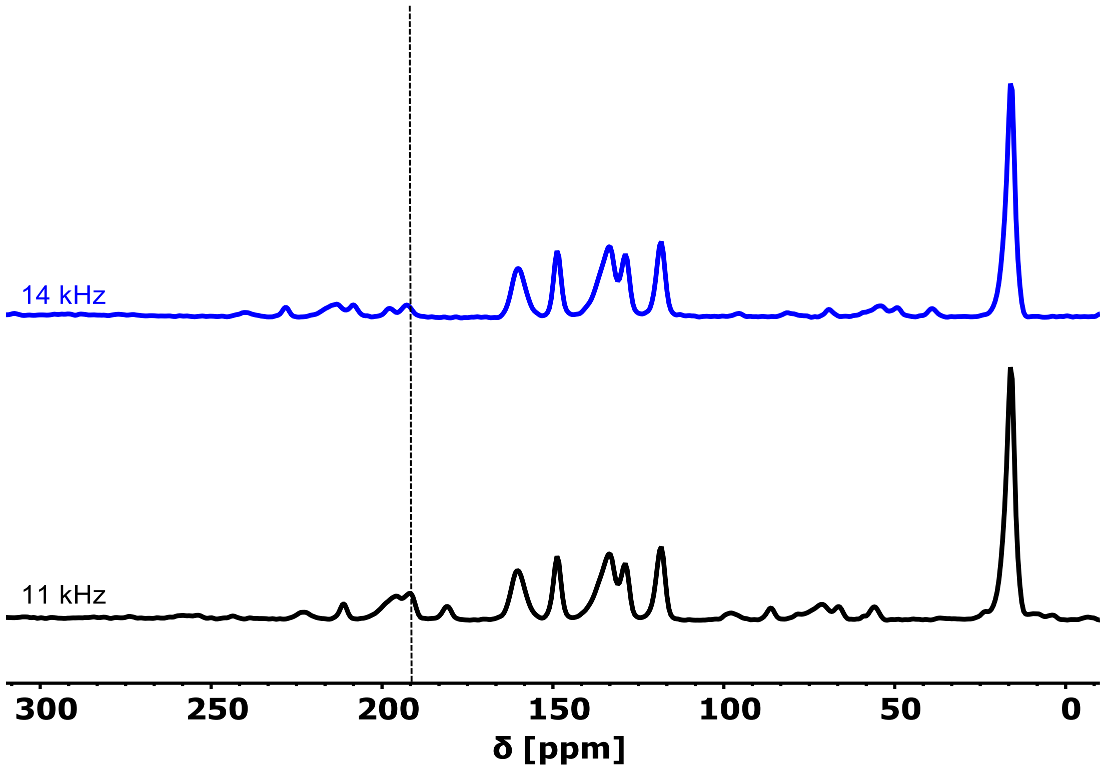

Figure S34. ^{13}C CPMAS solid state NMR spectra at 14 kHz and 11 kHz to determine spinning side bands. The vertical, dashed line indicates the chemical shift of residual aldehyde groups.

Me3TFB-Me4PA

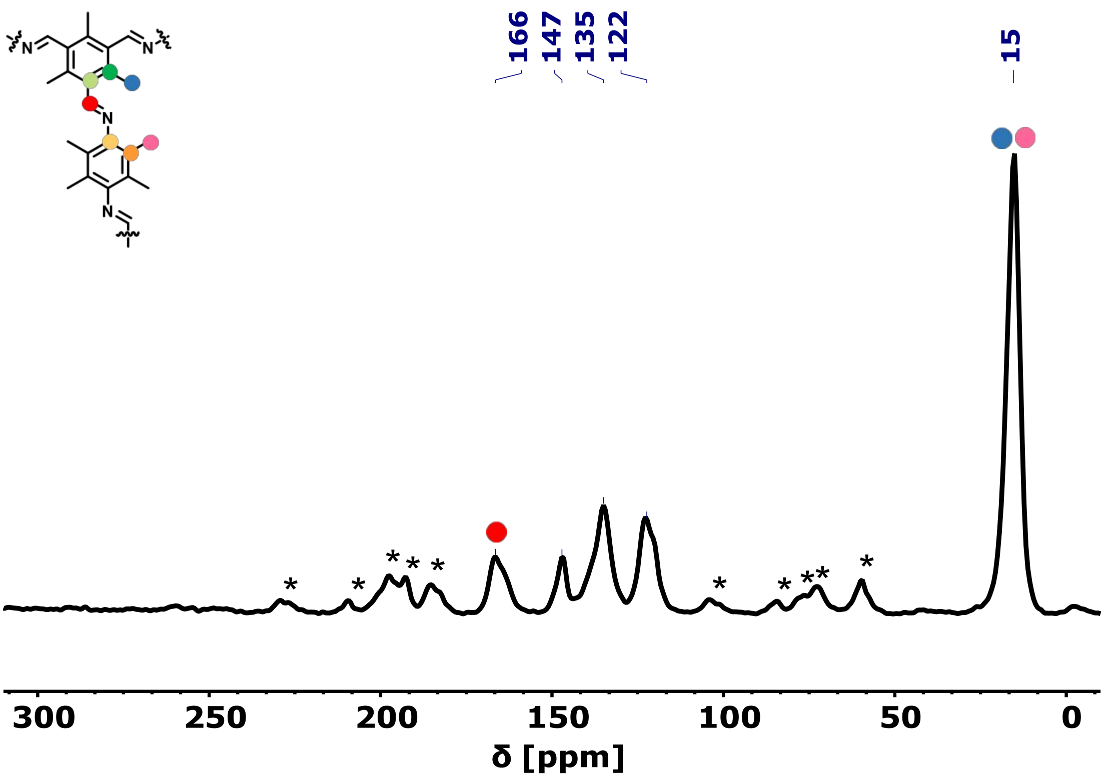

Figure S35. ^{13}C CPMAS solid state NMR spectrum at 11 kHz. Spinning side bands, here indicated with an asterisk were determined by comparing different MAS frequencies. The signal at 166 ppm can be assigned to the carbon of an imine-bound nitrogen, indicating the formation of imine bonds.

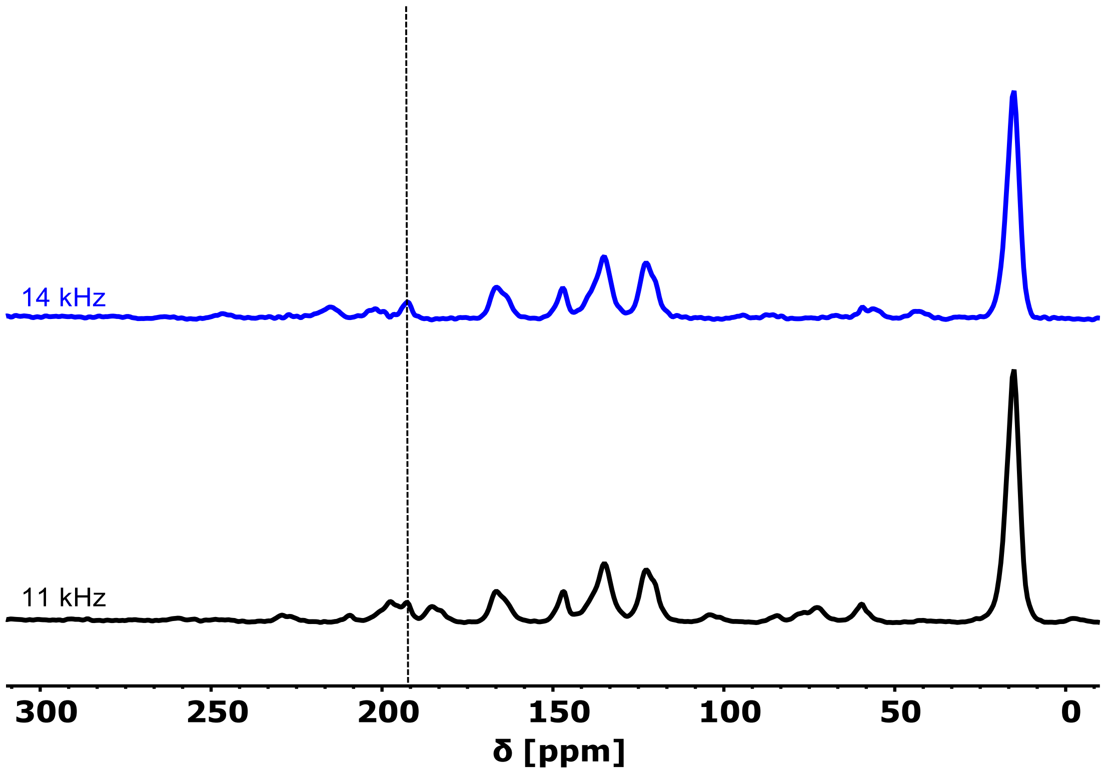

Figure S36. ^{13}C CPMAS solid state NMR spectra at 14 kHz and 11 kHz to determine spinning side bands. The vertical, dashed line indicates the chemical shift of residual aldehyde groups.

Me_3TFB-BD

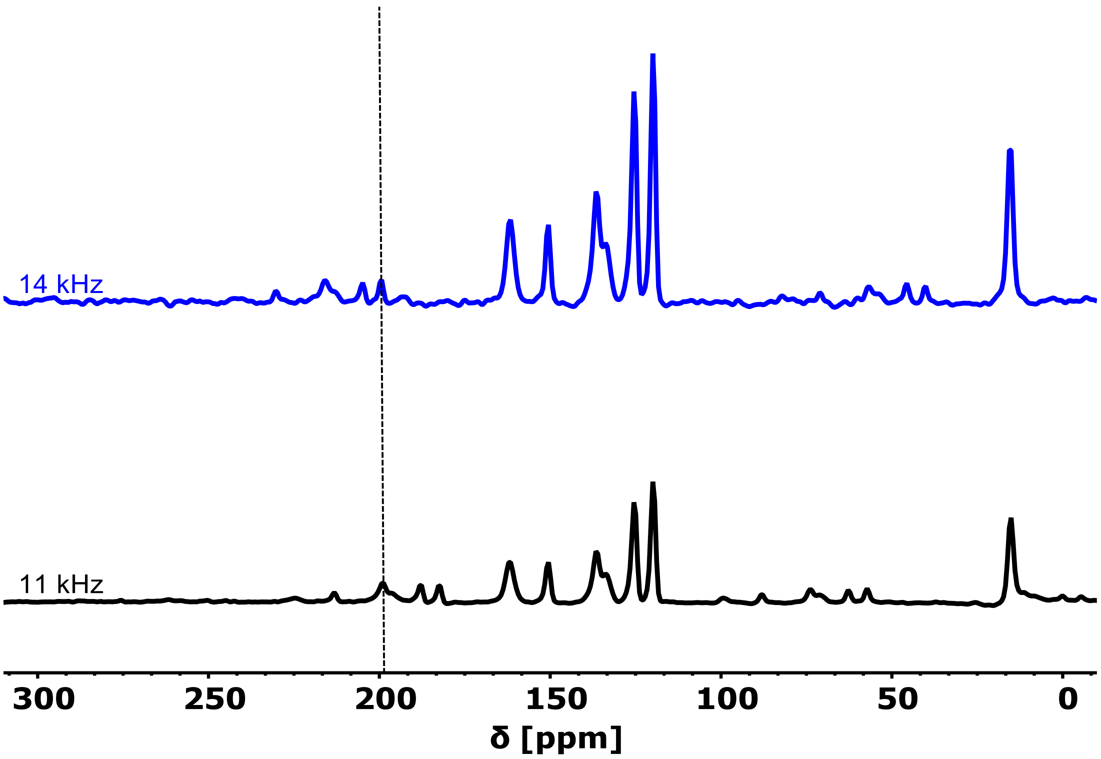

Figure S37. ^{13}C CPMAS solid state NMR spectra at 14 kHz and 11 kHz to determine spinning side bands. The vertical, dashed line indicates the chemical shift of residual aldehyde groups.

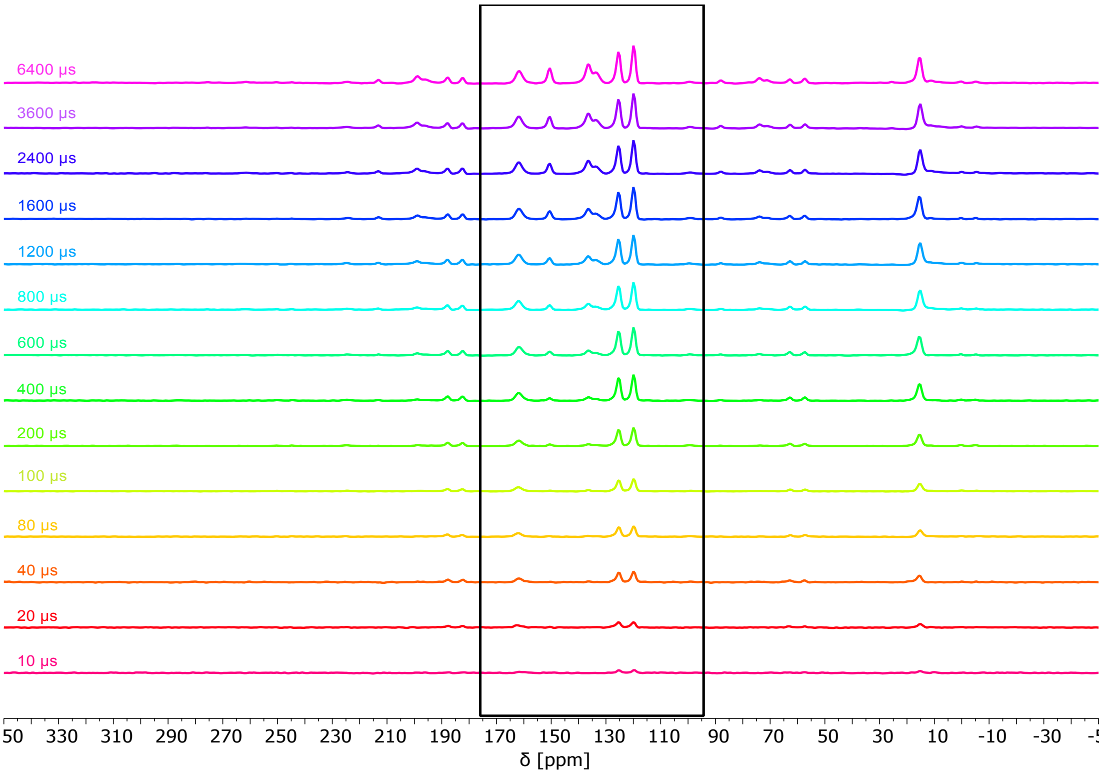

Figure S38. ^{13}C CPMAS solid state NMR spectra at 11 kHz and different contact times.

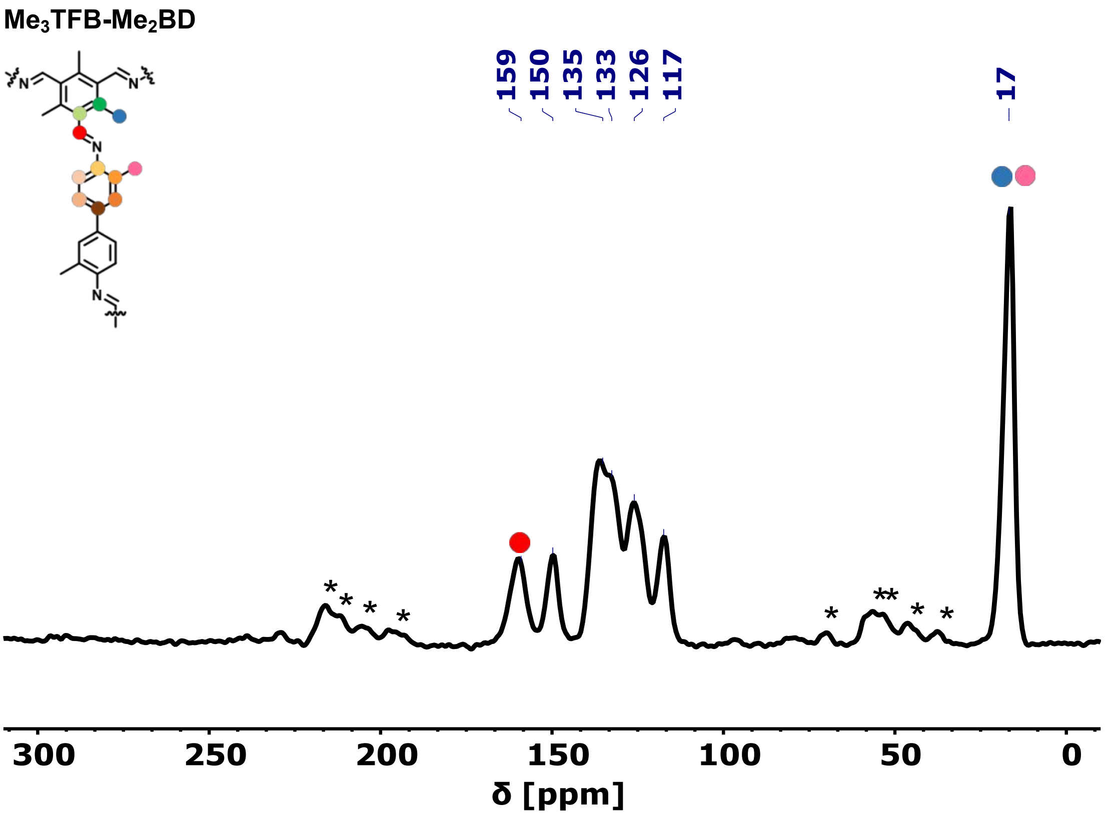

Figure S39. ^{13}C CPMAS solid state NMR spectrum at 11 kHz. Spinning side bands, here indicated with an asterisk were determined by comparing different MAS frequencies. The signal at 159 ppm can be assigned to the carbon of an imine-bound nitrogen, indicating the formation of imine bonds.

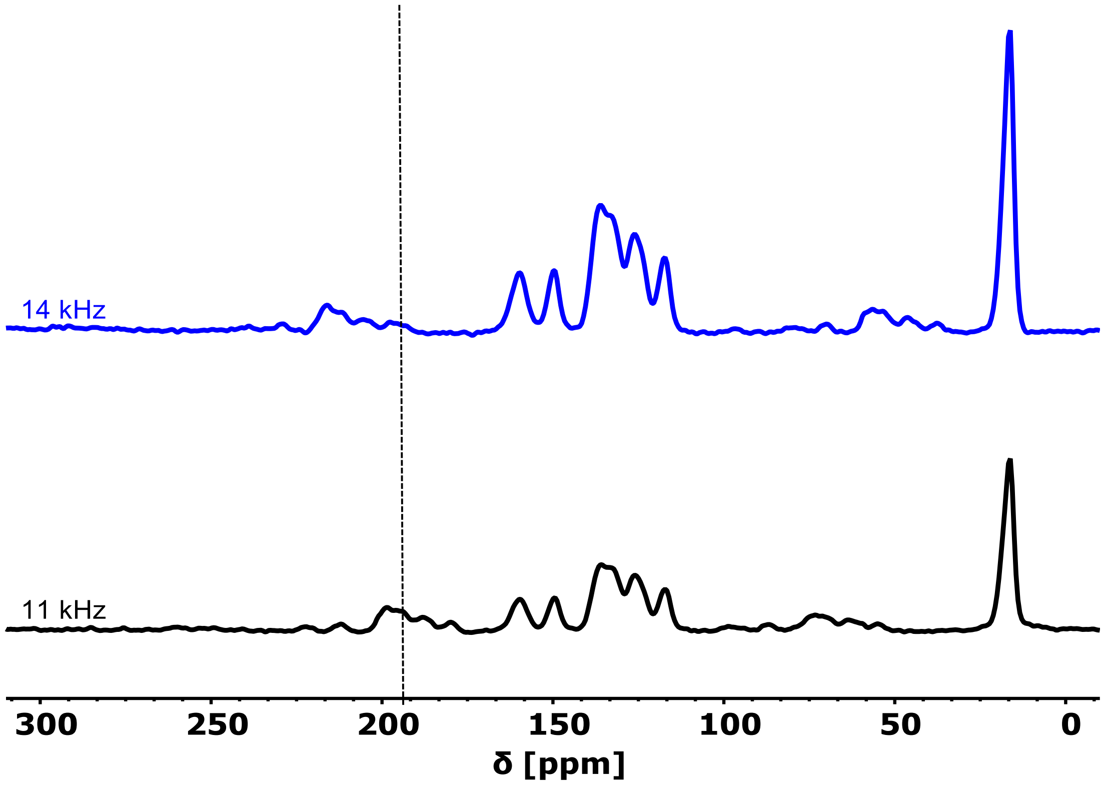

Figure S40. ^{13}C CPMAS solid state NMR spectra at 14 kHz and 11 kHz to determine spinning side bands. The vertical, dashed line indicates the chemical shift of residual aldehyde groups.

Me_3TFB-Me_4BD

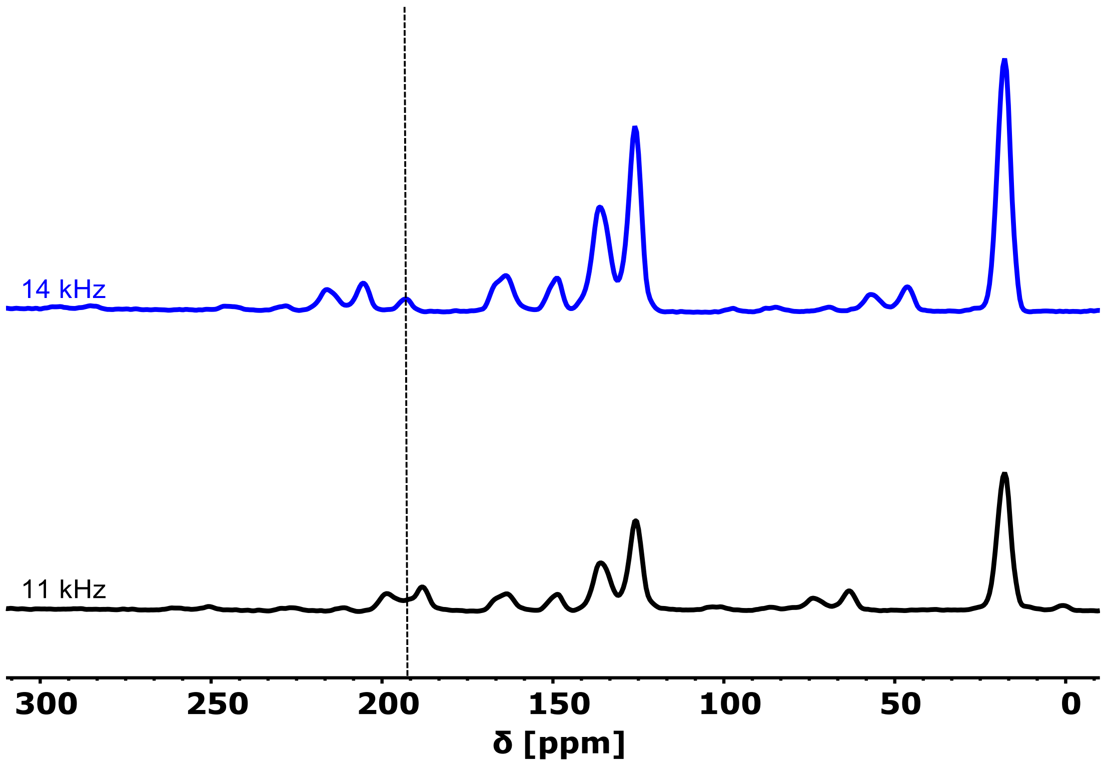

Figure S41. ^{13}C CPMAS solid state NMR spectra at 14 kHz and 11 kHz to determine spinning side bands. The vertical, dashed line indicates the chemical shift of residual aldehyde groups.

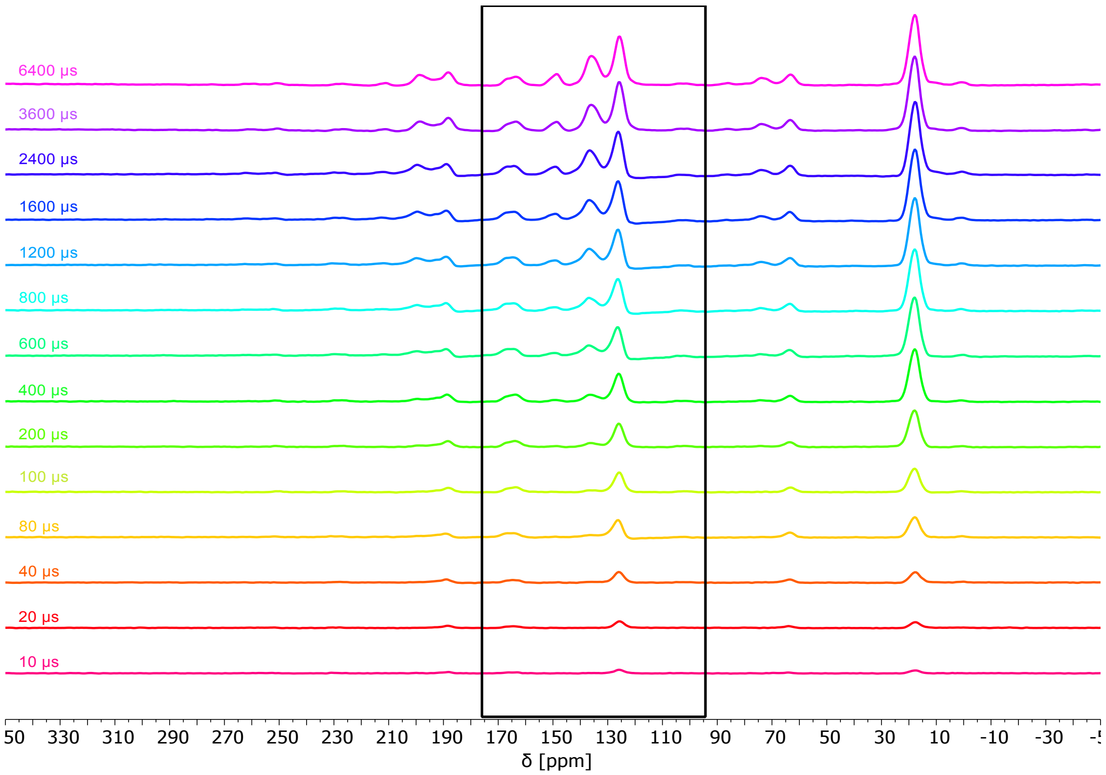

Figure S42. ^{13}C CPMAS solid state NMR spectra at 11 kHz and different contact times.

E. Nitrogen Sorption Analysis

All COFs have been synthesized three times and were then divided in two batches for different activation methods:

1. 120 °C, oven-dried, overnight
2. 120 °C, vacuum-oven-dried, overnight

For all samples nitrogen sorption analysis was carried out and the BET surface area has been calculated. The range for the linear regression has been the same for all repetitions of the same COF and for both activation methods. The same DFT model has been used for one COF structure to determine the pore size distribution. All graphs displayed are representative for the respective COF.

Me_3TFB-Me_2PA

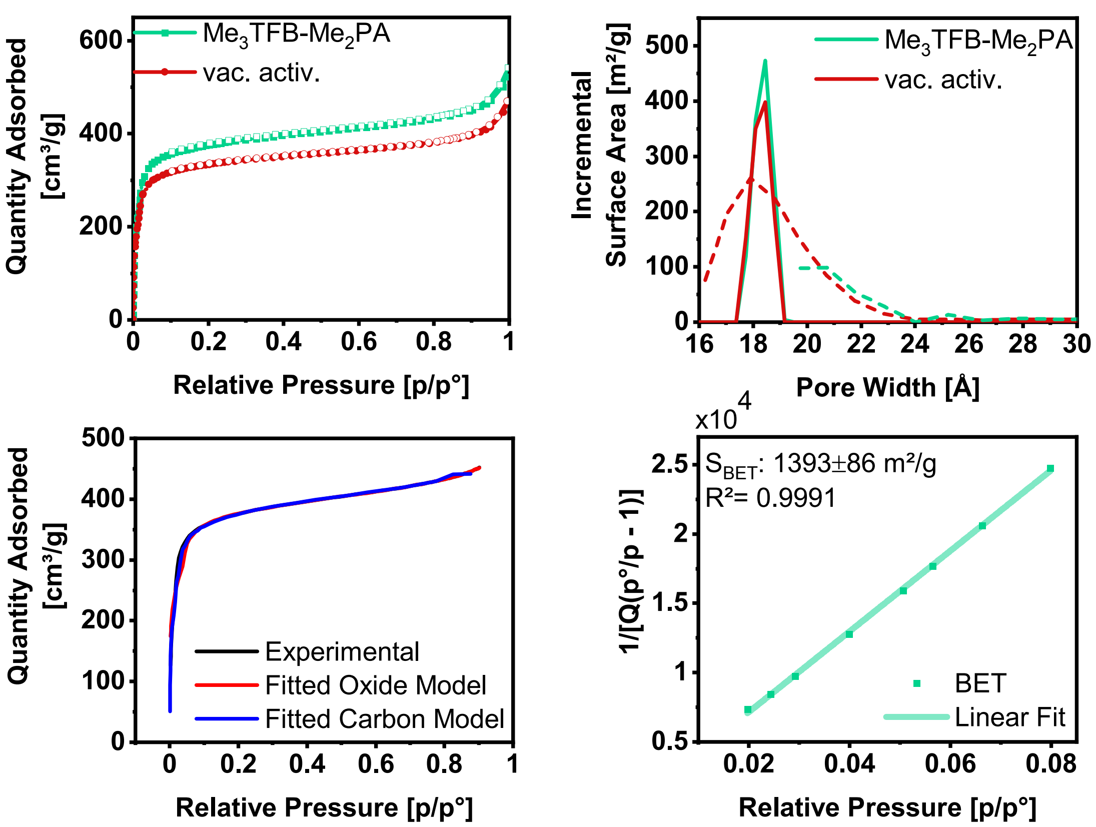

Figure S43. Top left: representative adsorption (solid symbols)-desorption (open symbols) isotherms for oven- and vacuum-oven activated COFs including their pore size distributions (top right). An NLDFT oxide surface model was used for the solid lines and a NLDFT carbon cylindrical pore model for the dashed lines. Bottom left: comparison of experimental adsorption isotherm with the theoretically modelled isotherms; bottom right: linear fit to calculate the BET surface area, including R^2.

Me_3TFB-Me_4PA

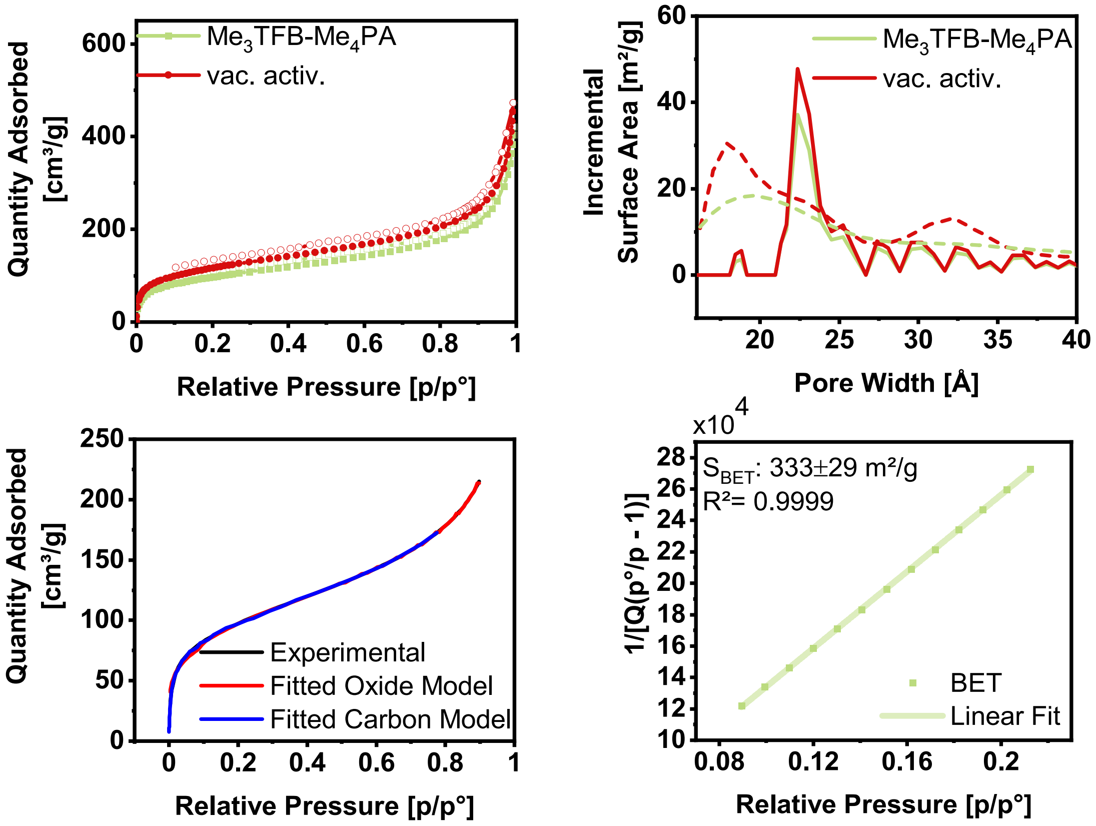

Figure S44. Top left: representative adsorption (solid symbols)-desorption (open symbols) isotherms for oven- and vacuum-oven activated COFs including their pore size distributions (top right). An NLDFT oxide surface model was used for the solid lines and a NLDFT carbon cylindrical pore model for the dashed lines. Bottom left: comparison of experimental adsorption isotherm with the theoretically modelled isotherms; bottom right: linear fit to calculate the BET surface area, including R^2.

Me_3TFB-Me_2BD

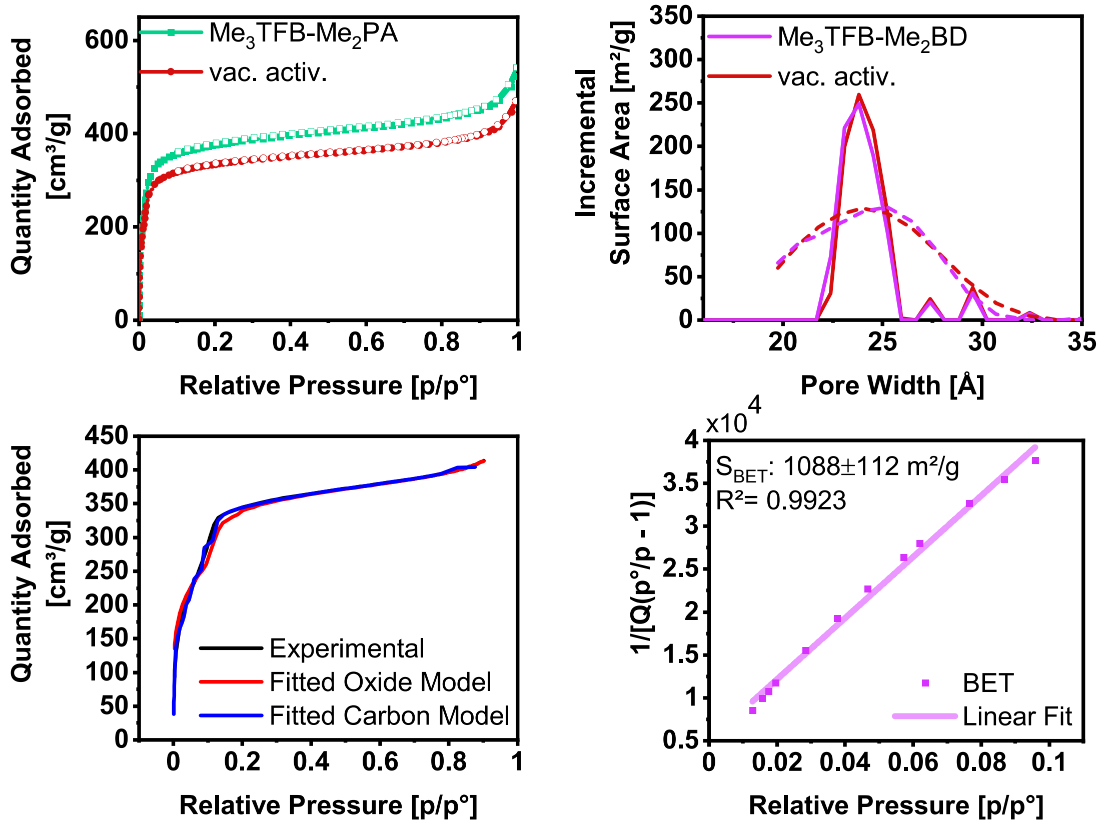

Figure S45. Top left: representative adsorption (solid symbols)-desorption (open symbols) isotherms for oven- and vacuum-oven activated COFs including their pore size distributions (top right). An NLDFT oxide surface model was used for the solid lines and a NLDFT carbon cylindrical pore model for the dashed lines. Bottom left: comparison of experimental adsorption isotherm with the theoretically modelled isotherms; bottom right: linear fit to calculate the BET surface area, including R^2.

Me_3TFB-Me_4BD

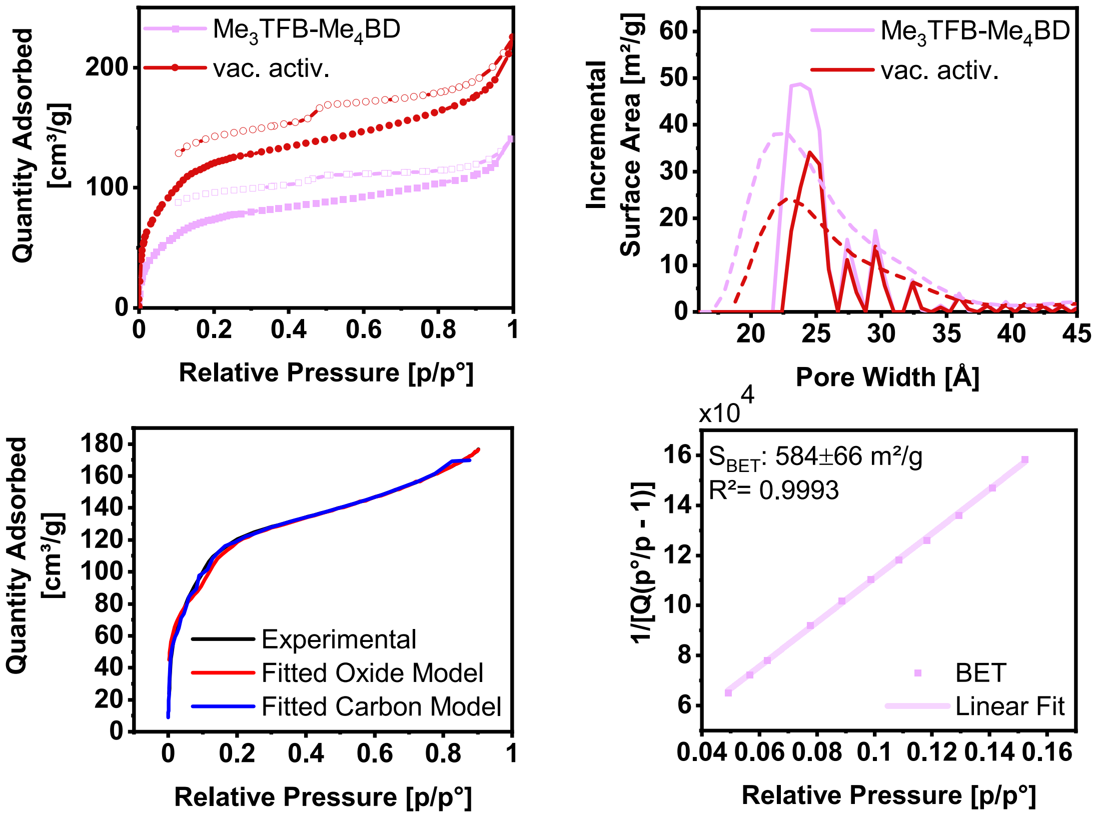

Figure S46. Top left: representative adsorption (solid symbols)-desorption (open symbols) isotherms for oven- and vacuum-oven activated COFs including their pore size distributions (top right). An NLDFT oxide surface model was used for the solid lines and a NLDFT carbon cylindrical pore model for the dashed lines. Bottom left: comparison of experimental adsorption isotherm with the theoretically modelled isotherms; bottom right: linear fit to calculate the BET surface area, including R^2.

TFB-Me$_2$PA

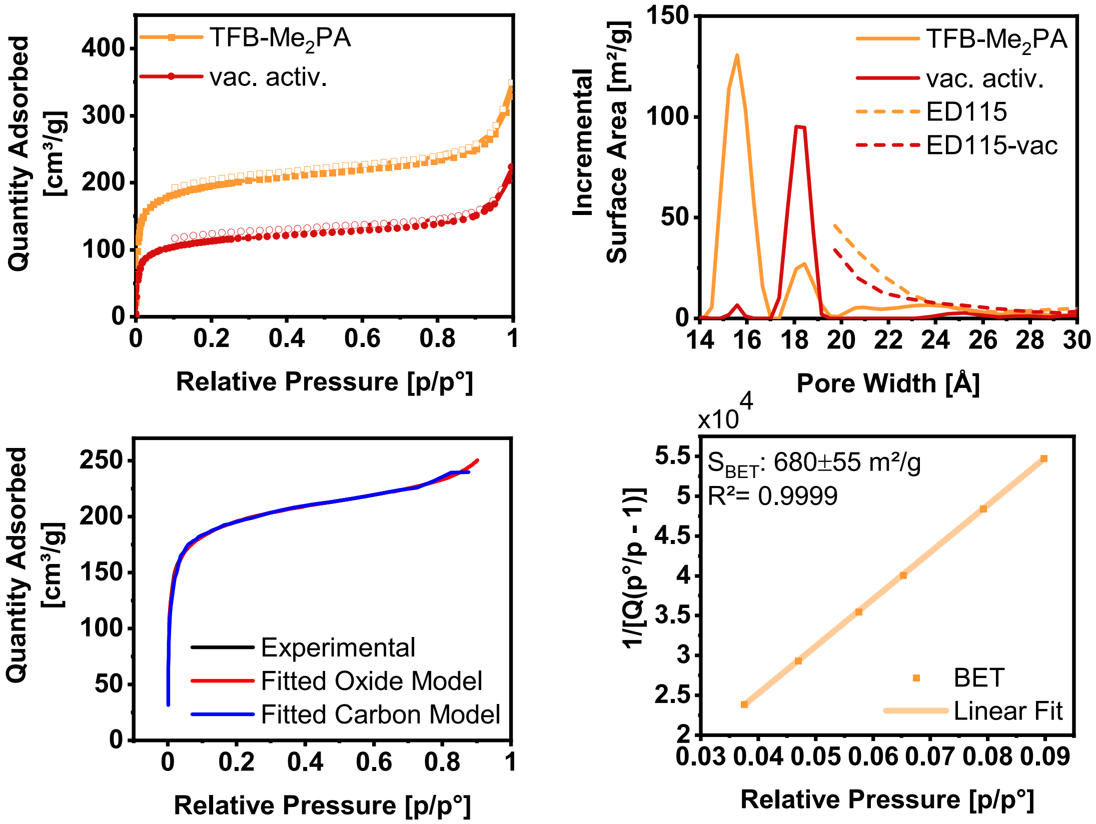

Figure S47. Top left: representative adsorption (solid symbols)-desorption (open symbols) isotherms for oven- and vacuum-oven activated COFs including their pore size distributions (top right). An NLDFT oxide surface model was used for the solid lines and a NLDFT carbon cylindrical pore model for the dashed lines. Bottom left: comparison of experimental adsorption isotherm with the theoretically modelled isotherms; bottom right: linear fit to calculate the BET surface area, including R^2.

TFB-Me$_4$PA

This COF has been tried to synthesize at least several times of which three samples were repeatable to each other, but in general this COF is not really repeatable in porosity. This is most likely due to the steric hinderance in combination with TFB which forms less stable COFs compared to Me$_3$TFB.

The repetition of this COFs have a small decrease in the adsorption isotherm between 0.37-0.93 and 0.25-0.89 *p/p^0* while the isotherm in Figure S48 does not show this decrease but a plateau. The surface area calculation is not affected by this decrease, because the used range for fitting is at lower relative pressures.

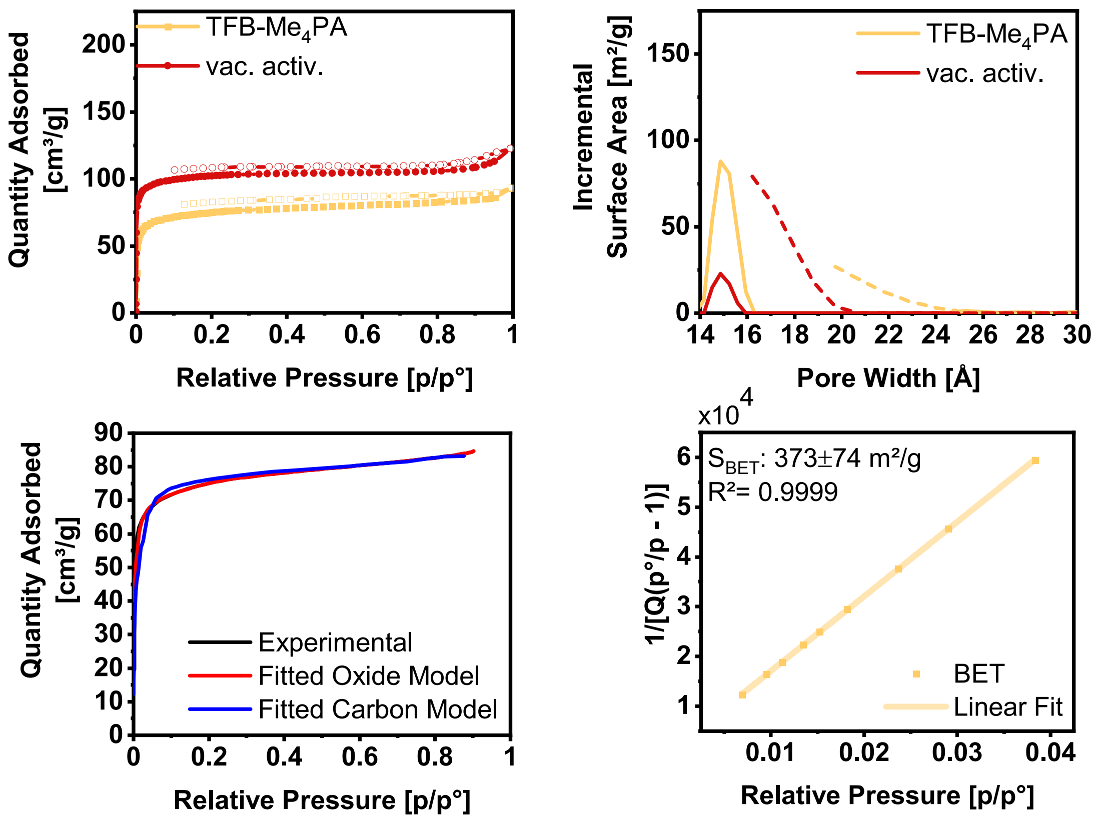

Figure S48. Top left: representative adsorption (solid symbols)-desorption (open symbols) isotherms for oven- and vacuum-oven activated COFs including their pore size distributions (top right). An NLDFT oxide surface model was used for the solid lines and a NLDFT carbon cylindrical pore model for the dashed lines. Bottom left: comparison of experimental adsorption isotherm with the theoretically modelled isotherms; bottom right: linear fit to calculate the BET surface area, including R^2.

TFB-Me$_2$BD

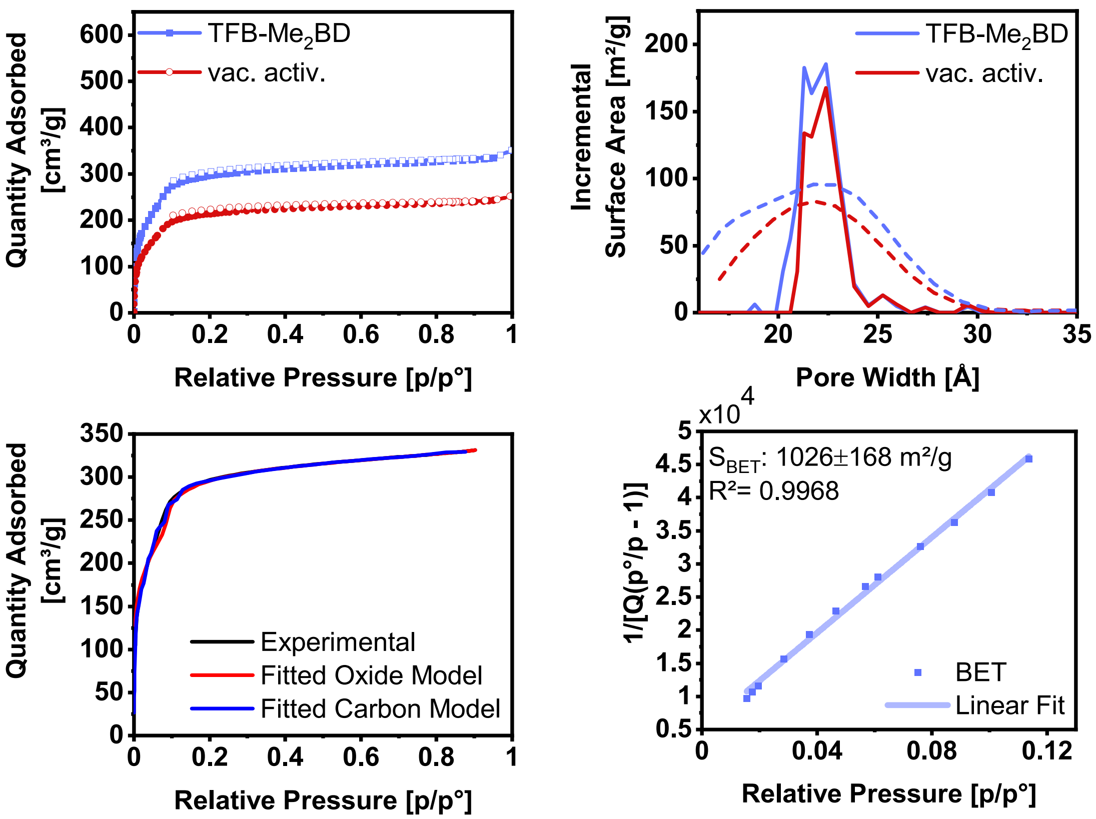

Figure S49. Top left: representative adsorption (solid symbols)-desorption (open symbols) isotherms for oven- and vacuum-oven activated COFs including their pore size distributions (top right). An NLDFT oxide surface model was used for the solid lines and a NLDFT carbon cylindrical pore model for the dashed lines. Bottom left: comparison of experimental adsorption isotherm with the theoretically modelled isotherms; bottom right: linear fit to calculate the BET surface area, including R^2.

TFB-Me$_4$BD

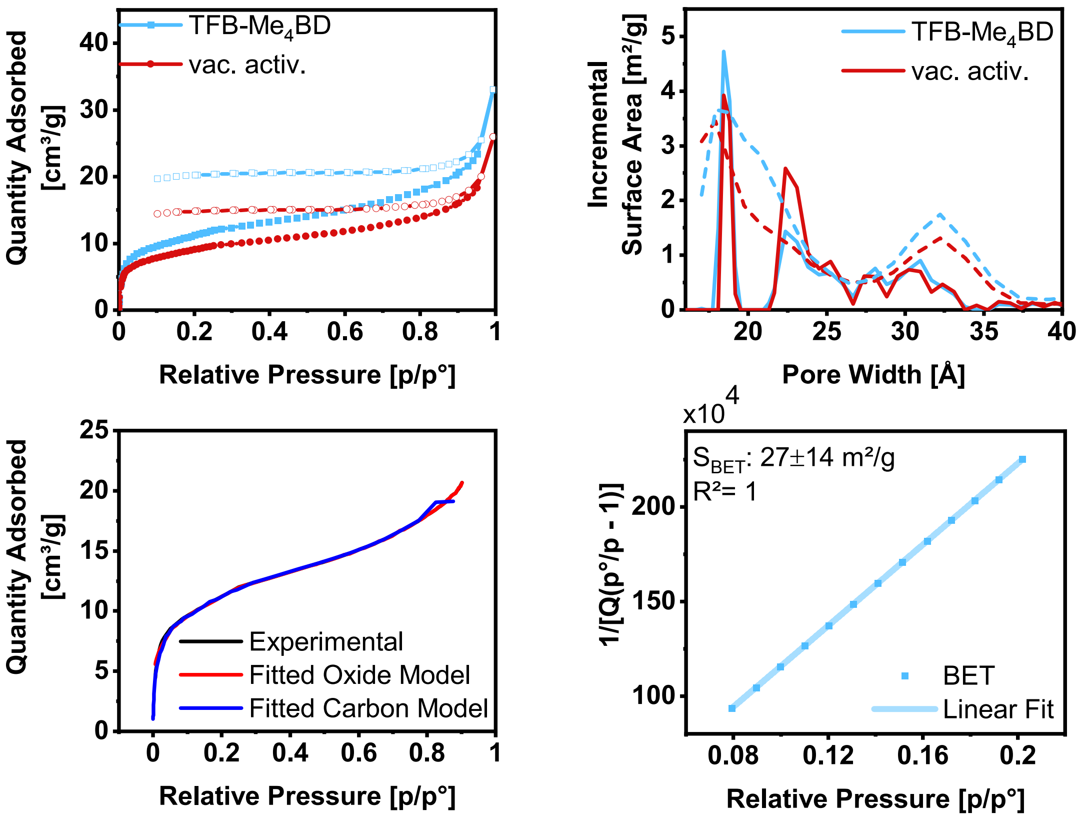

Figure S50. Top left: representative adsorption (solid symbols)-desorption (open symbols) isotherms for oven- and vacuum-oven activated COFs including their pore size distributions (top right). An NLDFT oxide surface model was used for the solid lines and a NLDFT carbon cylindrical pore model for the dashed lines. Bottom left: comparison of experimental adsorption isotherm with the theoretically modelled isotherms; bottom right: linear fit to calculate the BET surface area, including R^2.

F. Optical Properties

Samples were prepared by pressing the respective COF powder as a thin film between two glass plates. The samples were measured in an integrating sphere and the diffuse reflectance and diffuse transmission have been recorded. The incoming light beam (100%) gets partly reflected on the sample, partly absorbed by the sample and the rest of the light is transmitted. By detecting reflected and transmitted light, the absorbance can be calculated.[4]

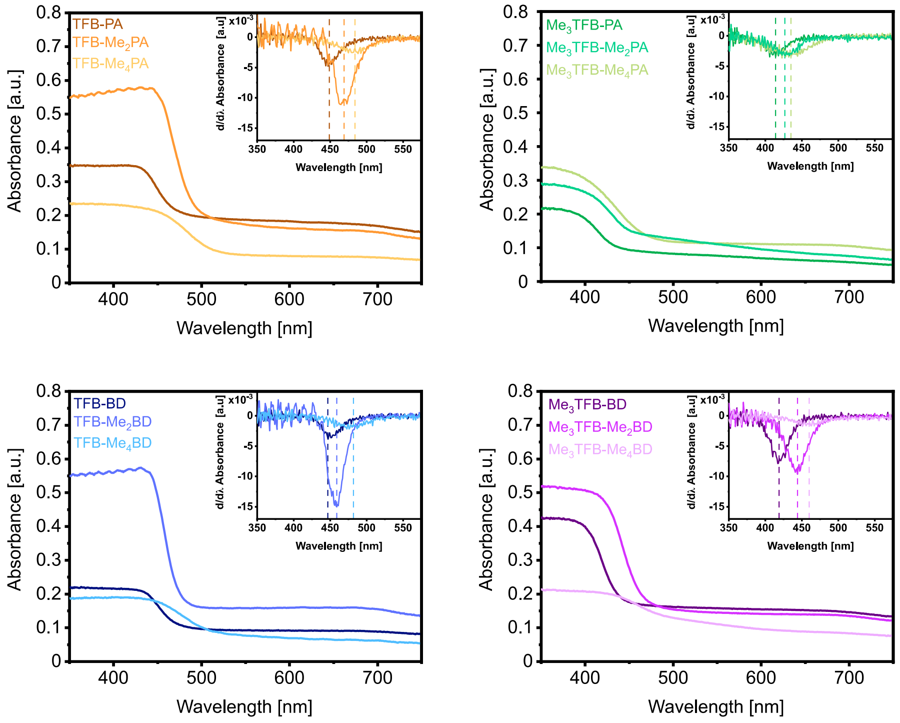

Figure S51: Absorbance spectra and their first derivative (inset) plotted for comparison of the number of methyl groups on TFB-Me$_x$PA (top right), Me$_3$TFB-Me$_x$PA (top left), TFB-Me$_x$BD (bottom right) and Me$_3$TFB-Me$_x$BD (bottom left).

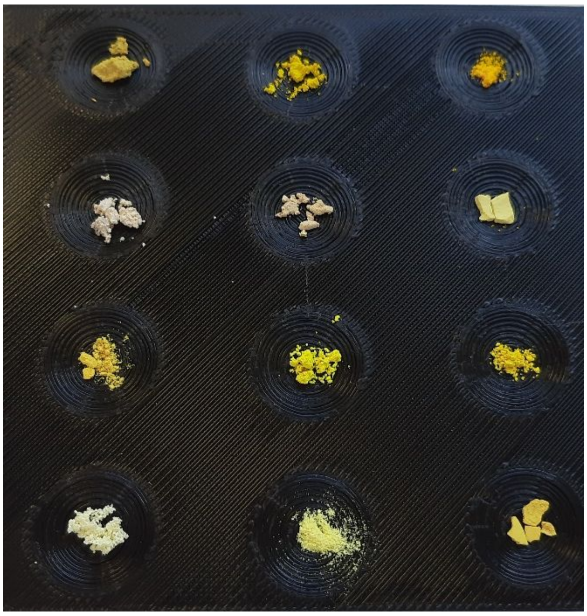

Figure S52: Optical photographs of TFB-Me_xPA (first row), Me_3TFB-Me_xPA (second row), TFB-Me_xBD (third row) and Me_3TFB-Me_xBd (fourth row). From left to right, the number of methyl groups increases.

G. Tauc plots

To obtain the optical bang gaps, the absorbance, as recorded in the measurement, was transformed into reflectance by neglecting the transmission contribution. The reflectance (R) is converted into the so-called Kubelka-Munk function (**Equation 2**) to calculate the optical band gap E_g. Kubelka and Munk[5] have developed a model for the appearance of paint films which is a continuous model using the two phenomenological parameters K (absorption coefficient) and S (scattering coefficient). Those can be derived from the reflectance by **Equation 3** and **Equation 4** assuming a direct allowed transition (n=1/2).[6,7] Out of these coefficients F(R) can be calculated by **Equation 5**.

$$(F(R)h\nu)^{\frac{1}{n}} = K\,(h\nu - E_g)\#(2)\ \#(3)$$

$$K = \ (1-R)^2\#(3)$$

$$S = 2 \times R\ \#(4)$$

$$F(R) = \frac{K}{S}\#(5)$$

For an adapted Tauc plot,[8] the wavelength is transformed into an energy in electron volt and plotted on the abscissa. The absorbance coefficient α from a normal Tauc plot is replaced by $(F(R)h\nu)^2$, where h is the Planck constant (6.63×10^{-34} J·s) and ν the frequency of the absorption in Hz, and plotted on the ordinate. The linear range is manually extrapolated to the abscissa and yields the optical band gap. The resulting optical band gaps rely on a single experiment.

Me_3TFB-PA

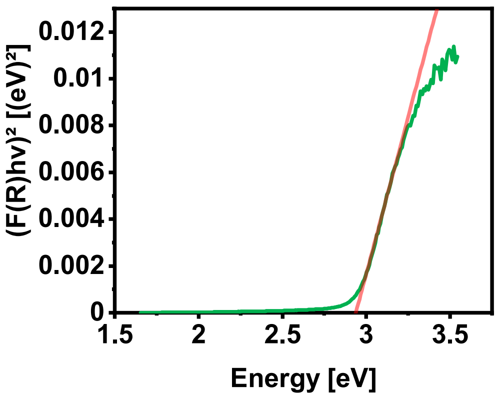

Figure S53. Kubelka-Munc function used for Tauc plot to determine the optical band gap.

Me_3TFB-Me_2PA

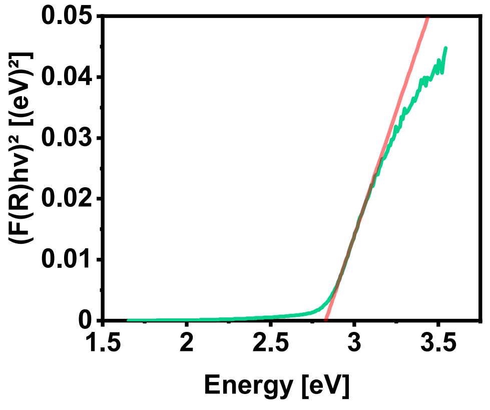

Figure S54. Kubelka-Munc function used for Tauc plot to determine the optical band gap.

Me_3TFB-Me_4PA

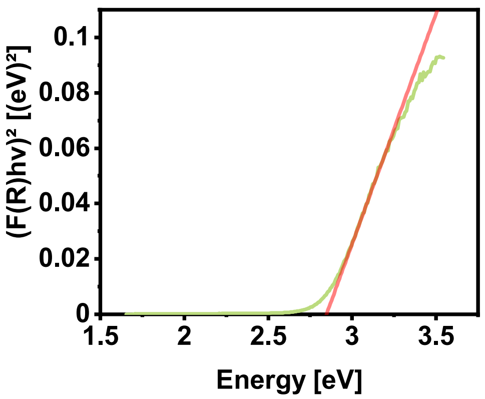

Figure S55. Kubelka-Munc function used for Tauc plot to determine the optical band gap.

Me_3TFB-BD

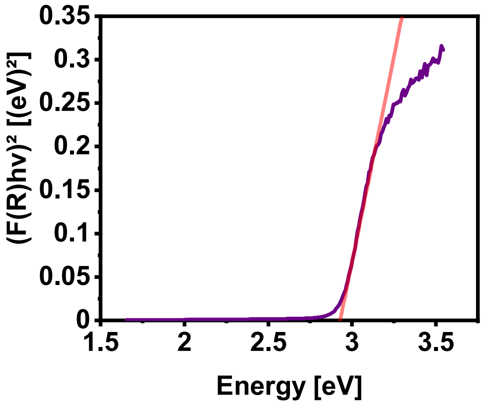

Figure S56. Kubelka-Munc function used for Tauc plot to determine the optical band gap.

Me_3TFB-Me_2BD

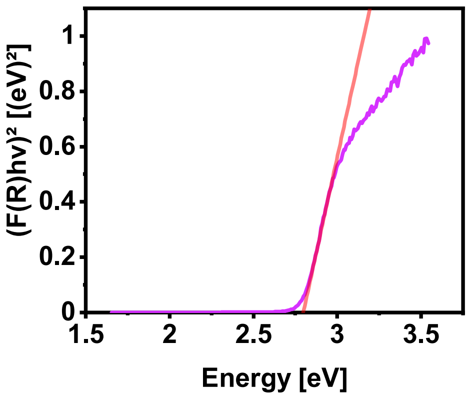

Figure S57. Kubelka-Munc function used for Tauc plot to determine the optical band gap.

Me_3TFB-Me_4BD

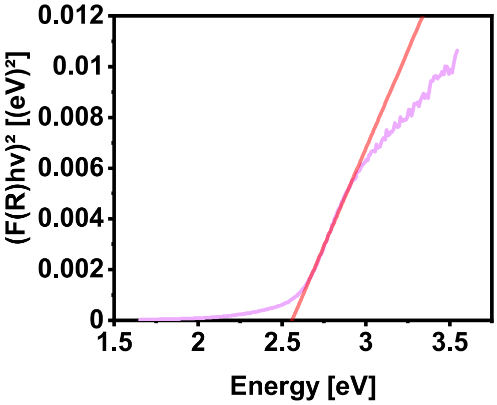

Figure S58. Kubelka-Munc function used for Tauc plot to determine the optical band gap.

TFB-PA

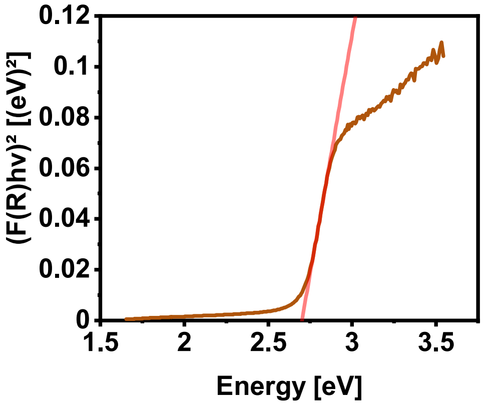

Figure S59. Kubelka-Munc function used for Tauc plot to determine the optical band gap.

TFB-Me_2PA

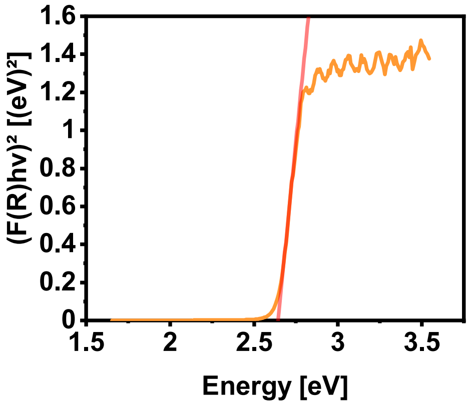

Figure S60. Kubelka-Munc function used for Tauc plot to determine the optical band gap.

TFB-Me$_4$PA

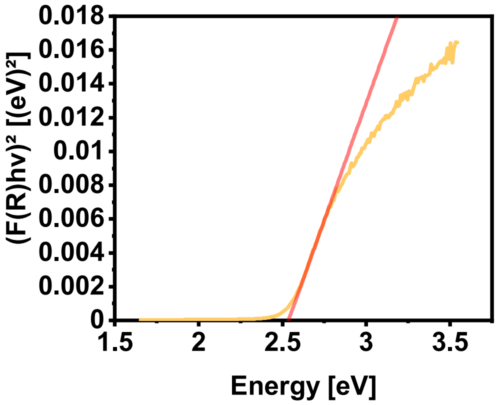

Figure S61. Kubelka-Munc function used for Tauc plot to determine the optical band gap.

TFB-BD

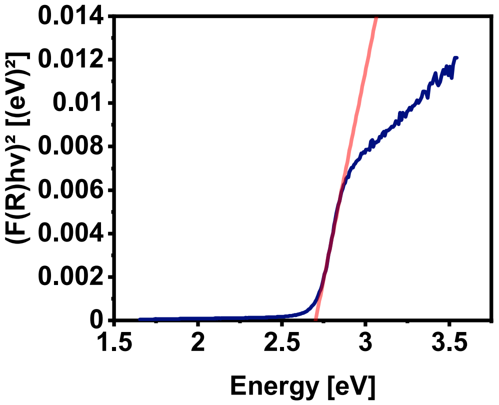

Figure S62. Kubelka-Munc function used for Tauc plot to determine the optical band gap.

TFB-Me$_2$BD

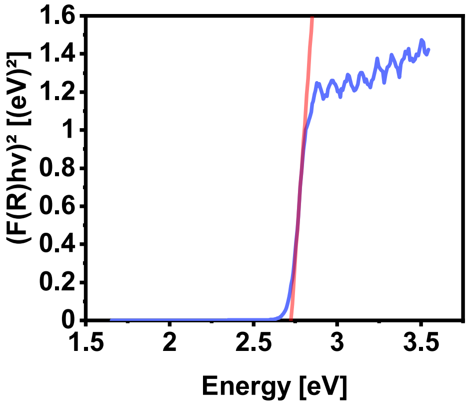

Figure S63. Kubelka-Munc function used for Tauc plot to determine the optical band gap.

TFB-Me$_4$BD

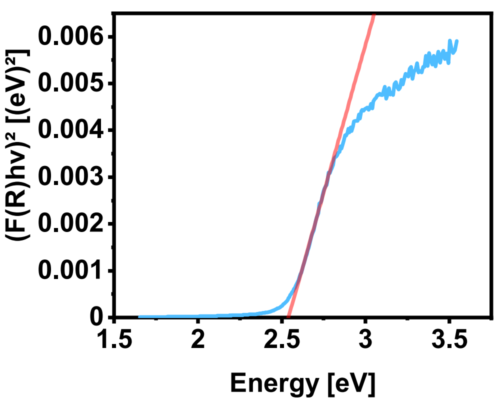

Figure S64. Kubelka-Munc function used for Tauc plot to determine the optical band gap.

H. Thermogravimetric analysis (TGA)

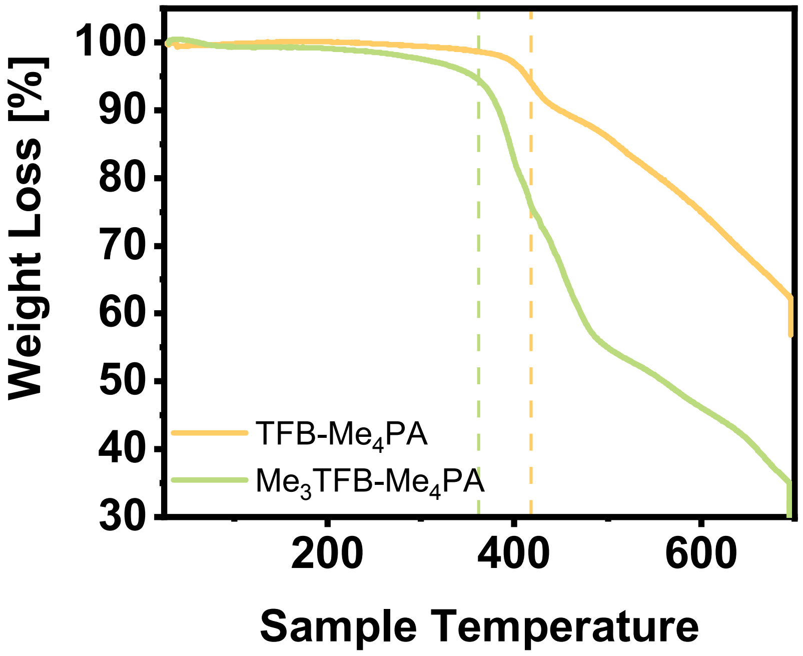

Figure S53. Thermogravimetric analysis of TFB-Me_4PA and Me_3TFB-Me_4PA. With a weight loss of only 5 %, the COFs are stable up to 412 °C and 363 °c, respectively.

I. DFT calculations

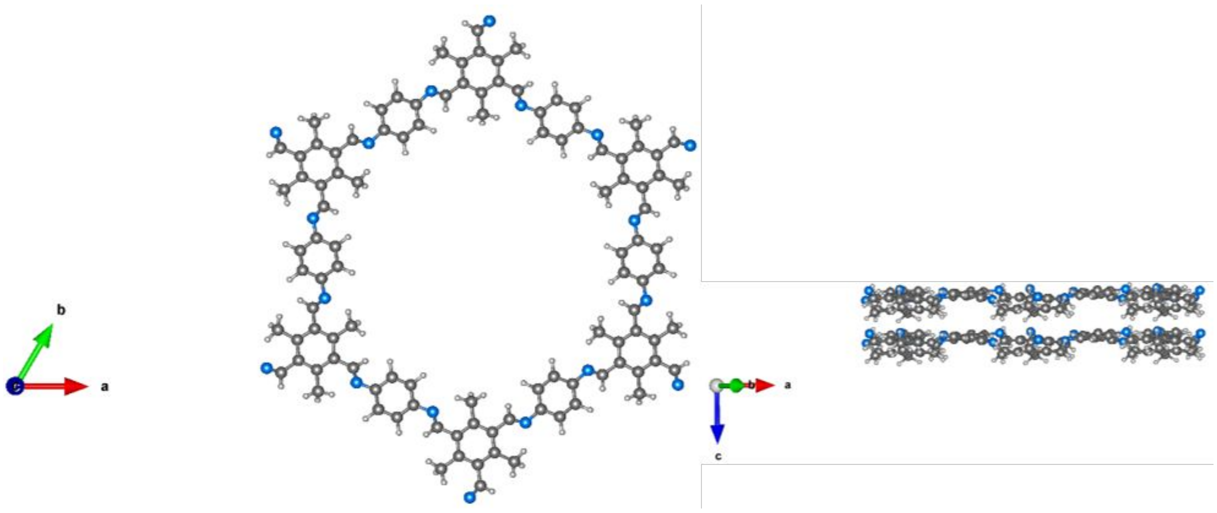

Figure S54. DFT-optimized crystal structure of Me_3TFB-PA from top view (left) and side view (right). Coordinates available as separate files.

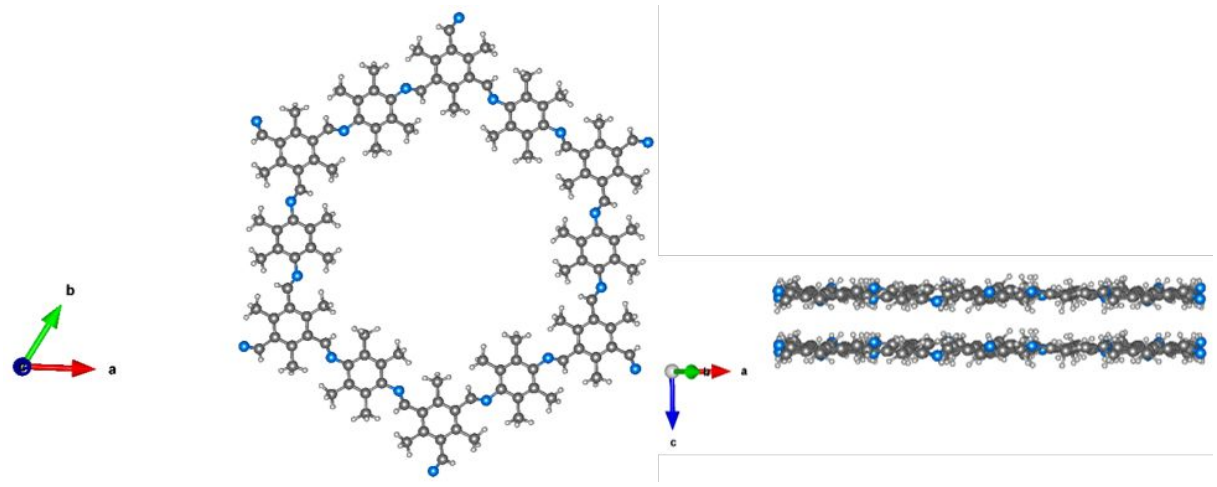

Figure S55. DFT-optimized crystal structure of Me_3TFB-Me_4PA from top view (left) and side view (right). Coordinates available as separate files.

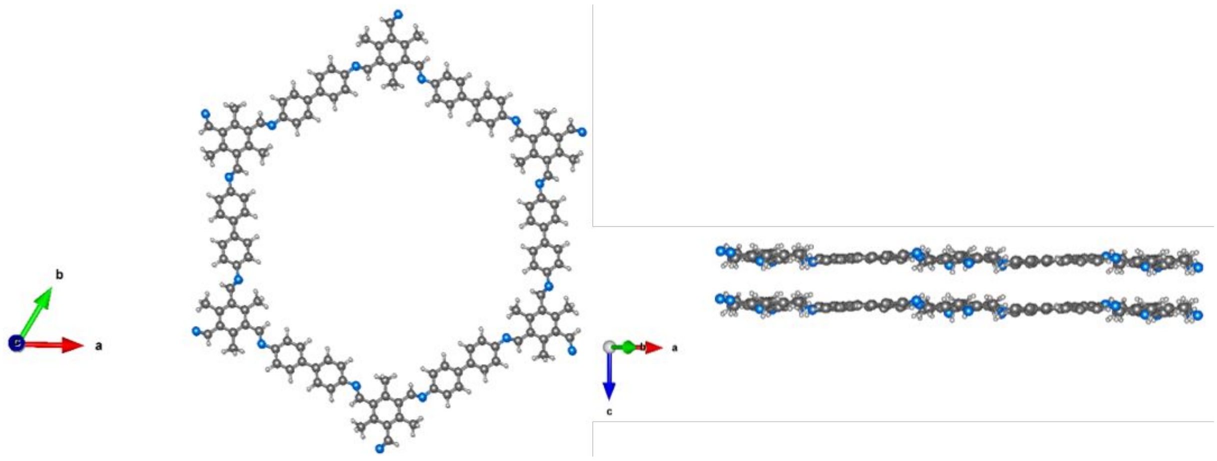

Figure S56. DFT-optimized crystal structure of Me_3TFB-BD from top view (left) and side view (right). Coordinates available as separate files.

J. References

(1) Dautzenberg, E.; Lam, M.; Li, G.; de Smet, L. C. P. M. Enhanced Surface Area and Reduced Pore Collapse of Methylated, Imine-Linked Covalent Organic Frameworks. *Nanoscale* **2021**, *13* (46), 19446–19452. https://doi.org/10.1039/d1nr05911d.

(2) van Meerten, S. G. J.; Franssen, W. M. J.; Kentgens, A. P. M. SsNake: A Cross-Platform Open-Source NMR Data Processing and Fitting Application. *J. Magn. Reson.* **2019**, *301*, 56–66. https://doi.org/10.1016/j.jmr.2019.02.006.

(3) Smith, B. J.; Overholts, A. C.; Hwang, N.; Dichtel, W. R. Insight into the Crystallization of Amorphous Imine-Linked Polymer Networks to 2D Covalent Organic Frameworks. *Chem. Commun.* **2016**, *52* (18), 3690–3693. https://doi.org/10.1039/c5cc10221a.

(4) Fest, E. Basic Radiometry for Stray Light Analysis. In *Stray Light Analysis and Control*; SPIE, 2013; Vol. PM229, pp 13–25. https://doi.org/10.1117/3.1000980.ch2.

(5) Kubelka, P.; Munk, F. An Article on Optics of Paint Layers. *Zeitschrift für Tech. Phys.* **1931**, *12*, 593–601.

(6) Kang, X.; Han, X.; Yuan, C.; Cheng, C.; Liu, Y.; Cui, Y. Reticular Synthesis of Tbo Topology Covalent Organic Frameworks. *J. Am. Chem. Soc.* **2020**, *142* (38), 16346–16356. https://doi.org/10.1021/jacs.0c06605.

(7) Viezbicke, B. D.; Patel, S.; Davis, B. E.; Birnie, D. P. Evaluation of the Tauc Method for Optical Absorption Edge Determination: ZnO Thin Films as a Model System. *Phys. Status Solidi* **2015**, *252* (8), 1700–1710. https://doi.org/10.1002/pssb.201552007.

(8) Tauc, J. Optical Properties and Electronic Structure of Amorphous Ge and Si. *Mater. Res. Bull.* **1968**, *3* (1), 37–46. https://doi.org/10.1016/0025-5408(68)90023-8.

(9) Kresse, G.; Furthmüller, J. Efficient Iterative Schemes for Ab Initio Total-Energy Calculations Using a Plane-Wave Basis Set. *Phys. Rev. B - Condens. Matter Mater. Phys.* **1996**, *54* (16), 11169–11186. https://doi.org/10.1103/PhysRevB.54.11169.

(10) Kresse G, Joubert, D. From Ultrasoft Pseudopotentials to the Projector Augmented-Wave Method. *Phys. Rev. B - Condens. Matter Mater. Phys.* **1999**, *59* (3), 1758–1775. https://doi.org/10.1103/PhysRevB.59.1758.

(11) Perdew, J. P.; Burke, K.; Ernzerhof, M. Generalized Gradient Approximation Made Simple. *Phys. Rev. Lett.* **1996**, *77* (18), 3865–3868. https://doi.org/10.1103/PhysRevLett.77.3865.

(12) Blöchl, P. E. Projector Augmented-Wave Method. *Phys. Rev. B* **1994**, *50* (24), 17953–17979. https://doi.org/10.1103/PhysRevB.50.17953.

(13) Grimme, S.; Ehrlich, S.; Goerigk, L. Effect of the Damping Function in Dispersion Corrected Density Functional Theory. *J. Comput. Chem.* **2011**, *32* (7), 1456–1465. https://doi.org/10.1002/jcc.21759.

(14) Momma, K.; Izumi, F. VESTA 3 for Three-Dimensional Visualization of Crystal, Volumetric and Morphology Data. *J. Appl. Crystallogr.* **2011**, *44* (6), 1272–1276. https://doi.org/10.1107/S0021889811038970.
